# Socio‐Economic Differences in the Oral Health of Irish Adolescents: The Potential Role of Behavioural, Material and Psychosocial Factors

**DOI:** 10.1111/cdoe.70043

**Published:** 2025-12-07

**Authors:** Vinay Sharma, Michael O'Sullivan, Lewis Winning, Oscar Cassetti, Aifric O'Sullivan, Bahman Honari, Michael Crowe

**Affiliations:** ^1^ Division of Restorative Dentistry and Periodontology Dublin Dental University Hospital, Trinity College Dublin Dublin 2 Ireland; ^2^ Institute of Food and Health Science Centre, South, UCD Dublin Ireland

**Keywords:** adolescents, cohort study, oral health, Republic of Ireland, self‐rated oral health, socio‐economic inequalities, young persons

## Abstract

**Background/Objectives:**

Socio‐economic inequalities in oral health are a universal phenomenon. This study investigated socio‐economic differences in Irish adolescents' oral health and the potential role of behaviour (oral health behaviours), material (structural, material and economic constraints) and psychosocial factors (parental stress and family structure) in these differences.

**Methods:**

Data analysed were from the first three waves of the Growing Up in Ireland child cohort survey on self‐ (self‐rated oral health (SROH)) and parent‐reported oral health outcomes (dental fillings) at age 17/18 years; socio‐economic status (SES) measures, behavioural, material and psychosocial factors at 13 years; and potential confounders at 9 years of age. Logistic regression was used to study associations between oral health outcomes and SES indicators and for mediation analysis.

**Results:**

Socio‐economic disadvantage was associated with poorer oral health outcomes, with gender‐specific patterns. Young males from the lowest educational and income groups had higher odds of suboptimal (fair/poor) self‐rated oral health (odds ratio (OR)_Education_: 2.31 (1.29; 4.13) and OR_Income_: 1.72 (1.16; 2.56)), and those in the lowest income quintile and with full medical cards had higher odds of dental fillings (ORs_Income_: 1.58–1.82 and ORs_Medical card_: 1.44–1.65) compared with higher socio‐economic groups. Young females showed significant associations between selected socio‐economic indicators (education, income, occupation and medical status) (ORs: 1.39–3.34) and dental fillings, with education demonstrating the strongest association (ORs_Education_: 1.91–3.34). For males, material, behavioural, and psychosocial factors mediated the SES–SROH relationship (97%–100%, 22%–69% and 5%–56% respectively), whereas for dental fillings, mediation was observed for material (11%–55%) and psychosocial (10%–37%) factors, with minimal mediation by behavioural factors (0%–2%). Among females, material factors were the primary mediators of the SES–dental fillings relationship (11%–55%), with smaller contributions from behavioural (0%–21%) and psychosocial (0%–26%) factors.

**Conclusion:**

Social disparities in oral health are common among Irish adolescents with gender‐specific patterns. Material factors were the primary pathway explaining these inequalities, though the strength and nature of these relationships vary by oral health outcome and gender.

**Abbreviations**
CES‐D: Centre for Epidemiological Studies Depression Scale
CSO: Central Statistics Office
CWF: Community Water Fluoridation
DCEDIY: Department of Children, Equality, Diversity, Integration and Youth
DEIS: Delivering Equality of Opportunity in Schools
DMFT: decayed, missing, and filled teeth (permanent tooth)
GUI: Growing Up in Ireland
PCG: Primary Care Giver
RoI: Republic of Ireland
SCG: Secondary Care Giver
SES: Socio‐Economic Status
SROH: Self‐Rated Oral Health
WHO: World Health Organization

## 1 Introduction

The World Health Organisation (WHO) recognises that oral health strongly correlates with socio‐economic status (SES) (income, educational level and occupation) across all countries [1]. While dental caries in children from high‐income countries have stabilised, rates continue to increase among lower income children, widening the socio‐economic gap in oral health outcomes [2].

Three main explanatory models (behavioural/cultural, psychosocial and materialistic) have been proposed to explain socio‐economic health inequalities [3, 4]. The behaviour hypothesis argues that people from lower social positions are more likely to participate in unfavourable/risky health behaviours than those from higher social positions; consequently, their health is worse than that of those from higher social positions [3]. A social gradient exists for oral health and oral health behaviours, and the dual relationship of oral health behaviours with oral health on one side and SES measures on the other suggests behaviours may play an important role in explaining social health inequalities in oral health [5, 6, 7]. The evidence suggested that oral health disparities could be eliminated or reduced by providing better access to preventive dental services and positive changes in individuals' health behaviours [8, 9, 10, 11]. However, others have argued that oral health disparities could not be improved solely by behavioural interventions [7, 12, 13].

The material explanation focuses on the role of structural, material and economic constraints in health inequalities [4]. It has been proposed that material factors, such as financial difficulties experienced by people in the lower SES groups, can restrict an individual's ability to afford the quality dental care and a healthy diet required for maintaining a healthy dentition [14, 15, 16]. It has been hypothesised that material factors could affect oral health either directly or indirectly via behavioural and psychosocial factors [14, 17]. When compared with higher SES groups, lower SES groups purchase higher amounts of foods and drinks rich in free sugars, a well‐known risk factor for dental caries [6, 14].

The psychosocial pathway emphasises the role of perceptions of control and social standing in influencing health, and hence, explaining health inequalities [4]. Those belonging to lower SES groups are hypothesised to experience higher levels of psychosocial stress and lower levels of social support than higher SES groups, which in turn have been shown to affect health directly or indirectly [16]. The proposed mechanisms through which psychosocial factors could impact oral health include their influence on oral health behaviours and impact on biological responses resulting in elevated inflammatory burden [16].

The life‐course approach offers another framework for understanding oral health inequalities. This theory posits that the oral health status reflects the cumulative impact of biological, environmental and social factors throughout an individual's life [18]. It has been suggested that these various explanatory pathways—behavioural, material, psychosocial, and life‐course—likely work in combination to create socio‐economic inequalities in oral health outcomes [19]. There is consistent evidence that social, behavioural and psychosocial processes relevant to health, including oral health, differ by sex during adolescence, influencing both exposure to and responses to health‐related risk factors [20, 21, 22].

To the best of the authors' knowledge, this is the first study to longitudinally investigate socio‐economic inequalities in adolescent oral health while comprehensively examining behavioural, material and psychosocial pathways. Using both self and parental‐reported data from Irish adolescents aged 17/18 years, this study aimed to: (1) examine the association between SES and oral health outcomes for the complete sample and by gender, and (2) assess the extent to which behavioural, material and psychosocial factors explain significant socio‐economic disparities identified within each gender group.

## 2 Methods

### 2.1 Study Population and Data Source

Growing Up in Ireland (GUI) is a multidomain, large‐scale and nationally representative longitudinal study carried out jointly by the Irish Department of Children, Equality, Diversity, Integration and Youth (DCEDIY) and the Central Statistics Office (CSO) [23]. The study commenced in 2006 and collected comprehensive data on a nationally representative sample of children. The study has two cohorts: The Infant and Child Cohort. The original GUI child cohort sample comprised *n* = 8568 children aged 9 years randomly selected from 910 primary schools in 2007. The study children were subsequently followed up at 13 (2011/12, *n* = 7525), 17/18 (2015/16, *n* = 6216) and 20 years (2018/19, *n* = 5190), and data were collected using face‐to‐face interviews.

This study has used data from the first three waves when the children in the GUI Child cohort were nine, 13, and 17/18 years of age. The present study included 6039 households that participated in all three waves. The GUI sample has been reweighted or statistically adjusted to ensure it is nationally representative of the age groups [24]. Further details of the GUI surveys are available at https://www.growingup.gov.ie/.

### 2.2 Study Measures

To establish temporal relationships between variables, data were collected across three waves: outcome measures from Wave 3, SES and behavioural/material/psychosocial factors from Wave 2, and potential confounding variables from Wave 1.

#### 2.2.1 Outcome Measures

Two primary outcome measures were assessed:

1. Self‐rated oral health (SROH): SROH is a validated indicator of oral health status that effectively captures oral health inequalities [8, 9, 12, 25]. At age 17/18, participants rated their oral health on a 5‐point Likert scale (Excellent, Very Good, Good, Fair, Poor). For analysis, responses were dichotomised into optimal (excellent/very good/good) and suboptimal (fair/poor) categories, consistent with previous research [15].
2. Number of Permanent Teeth with Dental Fillings: This measure serves as a quantitative indicator of cumulative dental caries experience [26, 27]. Primary caregivers reported the number of permanent teeth their child had filled, with response options of None, One, Two, or Three or more. This parent‐reported approach has been validated in multiple epidemiological studies [28, 29, 30, 31, 32, 33].

#### 2.2.2 Socio‐Economic Position Measures (Main Explanatory Variables)

Four key indicators were used to assess socio‐economic position:

##### 2.2.2.1 Primary Caregiver's Educational Level

Educational attainment was categorised into three levels: (1) None or primary education, (2) Secondary level, and (3) Tertiary level, following established conventions in health inequality research [15, 34].

##### 2.2.2.2 Family Income (Equivalised Income Quintiles)

Family income was assessed using equivalised household income quintiles, which account for household size and composition to enable meaningful comparisons. Households were ranked and divided into five equal groups, from the lowest to highest quintile [35].

##### 2.2.2.3 Occupation (Family Occupational Class)

Occupational class was determined using the higher social class category of either primary or secondary caregiver, following the dominance approach [36]. Classifications included: Professional/Managerial, Non‐manual/Skilled manual, and Semi‐skilled/Unskilled manual.

##### 2.2.2.4 Medical Card Status of Study Child

In the Republic of Ireland, medical card ownership has been used as an indicator of economic disadvantage [37, 38]. Regional Health Boards issue medical cards through a means‐tested system to individuals and their dependents who demonstrate financial inability to afford healthcare. Individuals with medical cards have access to primary, community and public hospital care [39].

#### 2.2.3 Behavioural Factors

Four behavioural factors were analysed and selected based on established literature [7, 12, 15, 40, 41]: Daily toothbrushing frequency (More than twice a day; Twice a day; Once a day; ‘Less than once a day’; Rarely/not at all), Dental visit patterns (At least a year; Once every 2 years; Once every 3 years; Only when there is a problem; Never/almost never) and consumption of sugary foods and sugary drinks (Once; More than Once; Not at all). Unfavourable health behaviours were defined as: brushing less than once daily, visiting dentists only for problems, and daily consumption of sugary foods or drinks [7, 15, 27, 42].

#### 2.2.4 Material Factors

The material factors assessed were financial difficulties (Yes; No), school disadvantage status (Delivering Equality of Opportunity in Schools (DEIS) school status) (Yes; No), private medical insurance (Yes; No) and home ownership (Owner; Renters) [15, 17, 40, 41, 43, 44].

#### 2.2.5 Psychosocial Factors

Psychosocial factors included based on published literature [14, 16, 43, 44, 45] were: (a) Family structure (single‐parent versus two‐parent households and the number of children) (Single‐parent family; Couple family); (b) PCG's depression status, assessed using the 8‐item short version of the Centre for Epidemiological Studies Depression Scale (CES‐D) scale (Depressed; Not depressed) [46]; (c) PCG's parental stress levels (measured using the Parental Stress Scale, higher scores indicate higher parental stress) [36]; (d) PCG's job‐related stress (Stressed; Not stressed).

#### 2.2.6 Covariates (Potential Confounders)

The analysis controlled for several potential confounding variables known to influence the relationship between SES measures and oral health outcomes. These confounders, measured during Wave 1 of the survey, included geographic area of residence (Urban; Rural), primary household language (English; Irish; Other), and the PCG's country of birth (used as a proxy indicator for ethnicity, Ireland; Outside Ireland) [15, 41, 47, 48].

**FIGURE 1:**
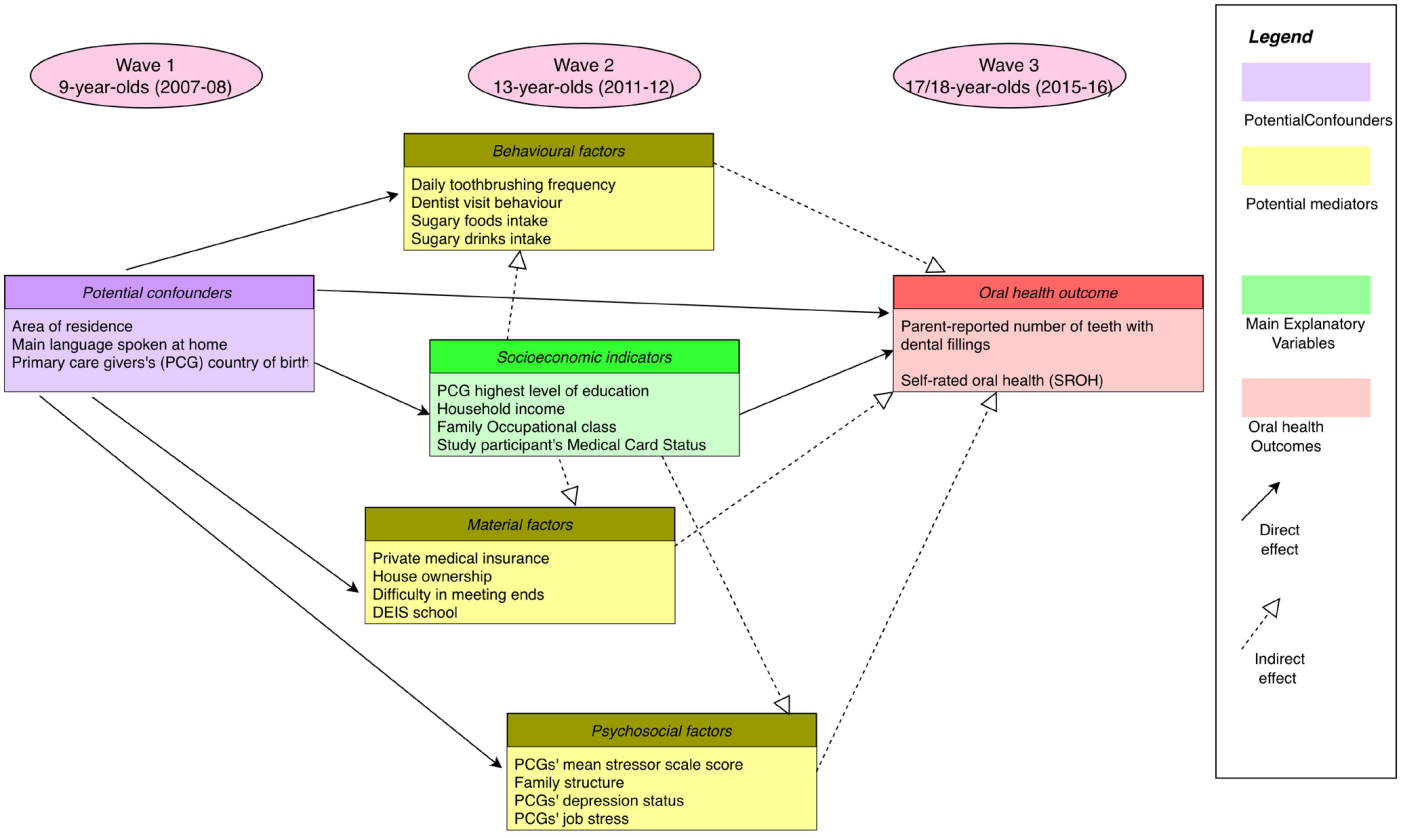
Directed acyclic graph describing the potential pathways in the relationship between socio‐economic measures at 13 years and young persons' oral health at 17/18 years of age.

Using the longitudinal design of the GUI survey, this study followed the temporal ordering of variables, as recommended in previous research [19, 41], to examine the proposed causal pathways between SES and oral health outcomes. The hypothesised relationships were visualised in a directed acyclic graph (DAG) (Figure 1), which maps the potential pathways linking socio‐economic measures at age 13 to oral health outcomes at age 17/18. Details of variable transformations performed prior to analysis are documented in Table S1 in File S1.

### 2.3 Statistical Analysis

Descriptive statistics were calculated for all variables, with categorical data presented as frequencies and percentages, and continuous data summarised using means and standard deviations. Gender differences in study variables were assessed using Chi‐square tests for categorical variables and independent *t*‐tests for continuous variables.

To investigate potential mediating pathways, we applied the Baron and Kenny conceptual framework [49], which has been previously validated in oral and general health inequality research [8, 12, 15, 22, 50].

This framework examined four key relationships:

- The association between SES (predictor) and oral health outcomes.
- The relationship between potential mediating factors and oral health outcomes.
- The relationship between SES (predictor) and mediating factors.
- The relationship between SES (predictor) and oral health outcomes after adjusting for mediating factors.

A series of logistic regression models was used to test these relationships systematically. Model 1 examined the relationship between oral health outcomes and SES while adjusting for key covariates (area of residence, primary household language, and PCG's country of birth). Subsequent models built upon this foundation, with Model 2 incorporating behavioural factors, Models 3 and 4 adding material and psychosocial factors respectively, and Model 5 examining all factors simultaneously.

All analyses were stratified by gender, with separate models estimated for males and females to examine potential gender‐specific associations between SES, mediating factors and oral health outcomes.

To quantify the mediating effects, the percentage change in odds ratios (PCOR) between models was calculated using the formula [17, 22]:

$$\begin{array}{cc} \text{PCOR}\;=\; & (\left({\text{OR}}_{\text{Reference model}}–{\text{OR}}_{\text{Reference}+\text{more}}\right)\\ & /\left({\text{OR}}_{\text{Reference model}}–1\right))\times 100 \end{array}$$

where OR_Reference model_ = OR obtained in the first model, OR_Reference + more_ = OR obtained after adding additional variables in the reference model and PCOR = Percentage change in the magnitude of the first OR.

When the odds ratio was < 1, the change was set at 100% to maintain consistent comparisons relative to the highest socio‐economic group. While this approach has limitations in comparing absolute changes across different odds ratios, it effectively estimates both direct and indirect effects of explanatory factors [17].

All analyses were conducted using R [51] and SPSS version 27.0, incorporating survey weights to ensure national representativeness. The analysis included only participants with complete data, and statistical significance was set at *p* < 0.05.

## 3 Results

A total of 6039 young persons participated in the first three waves of the GUI child cohort survey with a balanced gender distribution (49% male, 51% female) (Table 1). At age 17/18, ~16% of males and 18% of females had three or more permanent teeth with dental fillings, respectively. Approximately, 10% of males rated their oral health status as suboptimal (fair/poor) compared with 4% of females. Response rates varied across variables due to differences in the missing data.

**TABLE 1:** Distribution of study variables among young males and females (*N* = 6039).

Variable name	Variable category	Total *n* (%)	Males *n* (%)	Females *n* (%)	*p*
Socio‐demographic variables (covariates) Wave 1					
Region (*n* = 6025)	Rural	3375 (56.0)	1749 (56.9)	1626 (55.1)	0.178^a^
	Urban	2650 (44.0)	1327 (43.1)	1323 (44.9)	
Main language spoken at home (*n* = 6035)	Other	344 (5.7)	176 (5.7)	168 (5.7)	0.939^a^
	Irish	720 (11.9)	363 (11.8)	357 (12.1)	
	English	4971 (82.4)	2541 (82.5)	2430 (82.2)	
Citizenship of PCG (*n* = 6033)	Not Irish	361 (6.0)	183 (5.9)	178 (6.0)	0.893^a^
	Irish citizen	5672 (94.0)	2896 (94.1)	2776 (94.0)	
PCG's Country of birth (*n* = 6035)	Outside Ireland	898 (14.9)	457 (14.8)	441 (14.9)	0.925^a^
	Ireland	5137 (85.1)	2623 (85.2)	2514 (85.1)	
Socio‐economic variables (main explanatory variables) (Wave 2)					
Primary care giver's highest level of education (*n* = 6039)	None or primary	213 (3.5)	102 (3.3)	111 (3.8)	0.041^a^
	Secondary	4557 (75.5)	2294 (74.4)	2263 (76.5)	
	Tertiary	1269 (21.0)	686 (22.3)	583 (19.7)	
Family income—quintiles (*n* = 5586)	Lowest	1155 (20.7)	573 (20.1)	582 (21.3)	< 0.001^a^
	2nd	1125 (20.1)	562 (19.7)	563 (20.6)	
	3rd	1115 (20.0)	517 (18.1)	598 (21.9)	
	4th	1146 (20.5)	642 (22.5)	504 (18.4)	
	Highest	1045 (18.7)	558 (19.6)	487 (17.8)	
Family class (occupation) (*n* = 5481)	Semi‐skilled/unskilled manual	740 (13.5)	341 (12.1)	399 (15.0)	< 0.001^a^
	Other non‐manual/skilled manual	2035 (37.1)	994 (35.1)	1041 (39.3)	
	Professional managers	2706 (49.4)	1494 (52.8)	1212 (45.7)	
Medical card status (*n* = 6039)	Yes, full card	2096 (34.7)	1008 (32.7)	1088 (36.8)	0.001^a^
	Yes, doctor only card	141 (2.3)	66 (2.1)	75 (2.5)	
	Not covered	3802 (63.0)	2009 (65.2)	1793 (60.7)	
Behavioural factors (Wave 2)					
Toothbrushing frequency (*n* = 5962)	Less than once a day/rarely/not at all	327 (5.5)	223 (7.3)	104 (3.6)	< 0.001^a^
	Once a day	1260 (21.1)	779 (25.6)	481 (16.5)	
	Twice or more than twice a day	4375 (73.4)	2036 (67.0)	2339 (80.0)	
Dentist visit behaviour (*n* = 6037)	Problem visit/never/almost never	835 (13.8)	433 (14.0)	402 (13.6)	0.561^a^
	Occasional visit	1145 (19.0)	569 (18.5)	576 (19.5)	
	At least a year	4057 (67.2)	2081 (67.5)	1976 (66.9)	
Sugary drinks intake (soft drinks/minerals/cordial/squash (not diet)) (*n* = 5950)	More than once a day	1070 (18.0)	587 (19.4)	483 (16.6)	< 0.001^a^
	Once a day	1706 (28.7)	991 (32.7)	715 (24.5)	
	Not at all	3174 (53.3)	1454 (48.0)	1720 (58.9)	
Sugary foods intake (biscuits, doughnuts, cake, pie or chocolate) (*n* = 5957)	More than once a day	1180 (19.8)	615 (20.3)	565 (19.3)	0.351^a^
	Once a day	2968 (49.8)	1521 (50.1)	1447 (49.5)	
	Not at all	1809 (30.4)	897 (29.6)	912 (31.2)	
Material factors (Wave 2)					
Financial difficulties (difficulty in meeting ends) (*n* = 6036)	With difficulty	3631 (60.2)	1792 (58.2)	1839 (62.2)	0.001^a^
	Without difficulty	2405 (39.8)	1288 (41.8)	1117 (37.8)	
Private medical insurance (*n* = 6037)	No	3030 (50.2)	1451 (47.1)	1579 (53.4)	< 0.001^a^
	Yes	3007 (49.8)	1631 (52.9)	1376 (46.6)	
DEIS school (*n* = 5593)	Yes	1015 (18.1)	525 (18.3)	490 (18.0)	0.792^a^
	No	4578 (81.9)	2347 (81.7)	2231 (82.0)	
Homeownership (*n* = 6034)	Renters/rent free/others	1155 (19.1)	518 (16.8)	637 (21.6)	< 0.001^a^
	Owners	4879 (80.9)	2562 (83.2)	2317 (78.4)	
Psychosocial factors (Wave 2)					
PCG Parental Stressor scale score (*n* = 5975)	Mean (SD)	10.2 (4.17)	10.36 (4.29)	10.23 (4.04)	0.214^b^
Family structure (*n* = 6039)	Single‐parent family	1115 (18.5)	532 (17.3)	583 (19.7)	0.014^a^
	Couple family	4924 (81.5)	2551 (82.7)	2373 (80.3)	
Depression status (*n* = 5978)	Depressed	670 (11.1)	353 (11.6)	317 (10.8)	0.353^a^
	Not depressed	5308 (87.9)	2695 (88.4)	2612 (89.2)	
Job stress (*n* = 5680)	Stressed	3833 (67.5)	1918 (66.1)	1915 (68.9)	0.022^a^
	Not stressed	1847 (32.5)	984 (33.9)	863 (31.1)	
Young persons' oral health (outcome variables) (Wave 3)					
Parent‐reported oral health (Number of permanent teeth with fillings) (*n* = 5859)	None	2978 (50.8)	1591 (53.5)	1387 (48.1)	< 0.001^a^
	One	879 (15.0)	428 (14.4)	451 (15.6)	
	Two	1029 (17.6)	490 (16.5)	539 (18.7)	
	Three or more	973 (16.6)	467 (15.7)	506 (17.6)	
Self‐reported oral health (self‐rated oral health, SROH) (*n* = 6033)	Optimal oral health (excellent/very good/good)	5588 (92.6)	2767 (89.8)	2821 (95.6)	< 0.001^a^
	Suboptimal oral health (fair/poor)	445 (7.4)	315 (10.2)	130 (4.4)	

^a^ Chi‐square test.

^b^ Independent *t*‐test.

### 3.1 SES and Oral Health Outcomes

Bivariate logistic regression analysis showed that study participants from lower social groups generally had higher odds of having suboptimal (fair/poor) SROH and dental fillings (‘two teeth with dental fillings’ and ‘three or more teeth with dental fillings’) compared with those from higher social groups (Tables S2 and S3 in File S1). Stratification by gender showed that patterns differed between young males and young females (Tables S2 and S4 in File S1).

Young males from the lowest educational and income groups had higher odds of suboptimal SROH (odds ratio (OR)_Education_: 2.31 (1.29; 4.13) and OR_Income_: 1.72 (1.16; 2.56)) compared with higher socio‐economic groups (Table 2). Young males in the lowest income quintile and with full medical cards had higher odds of having dental fillings (ORs_Income_: 1.58–1.82 and ORs_Medical card_: 1.44–1.65) (Tables 3 and 4). Young females showed stronger associations with dental fillings across four selected socio‐economic indicators (education, income, occupation and medical status). For young females, the odds of having dental fillings were higher for those from the lowest educational, family income, occupational and ‘medical card’ groups compared with their higher social counterparts. The odds ratio ranged between 1.39 and 3.34, with education demonstrating the strongest association (ORs_Education_: 1.91–3.34) (Tables 3 and 4).

**TABLE 2:** Association between PCGs' highest educational level/family income and young males' self‐reported oral health (self‐rated oral health) adjusted for covariates (Wave 1), behavioural factors, material factors and psychosocial factors (logistic regression odds ratios (95% CI) for self‐rated suboptimal oral health).

	Model 1	Model 2	PCOR (%)	Model 3	PCOR (%)	Model 4	PCOR (%)	Model 5	PCOR (%)
PCG highest level of education									
None or primary	**2.31 (1.29; 4.13)**	**2.02 (1.10; 3.68)**	22.14	0.95 (0.46; 1.97)	100.00	1.57 (0.78; 3.15)	56.49	1.16 (0.56; 2.39)	87.79
Secondary	1.33 (0.98; 1.81)	1.14 (0.83; 1.58)	57.58	1.00 (0.72; 1.39)	100.00	1.29 (0.94; 1.78)	12.12	0.92 (0.65; 1.31)	100.00
Tertiary (reference)									
Family income quintiles									
Lowest	**1.72 (1.16; 2.56)**	1.22 (0.80; 1.86)	69.44	1.02 (0.64; 1.65)	97.22	**1.68 (1.11; 2.53)**	5.56	0.80 (0.49; 1.28)	100.00
2nd	**1.91 (1.29; 2.82)**	1.46 (0.97; 2.20)	49.45	1.18 (0.74; 1.88)	80.22	**1.65 (1.10; 2.50)**	28.57	0.90 (0.56; 1.43)	100.00
3rd	1.25 (0.82; 1.91)	0.94 (0.60; 1.47)	100.00	0.89 (0.55; 1.43)	100.00	1.27 (0.82; 1.97)	−8.00	0.67 (0.41; 1.09)	100.00
4th	1.08 (0.71; 1.63)	0.98 (0.64; 1.50)	100.00	0.85 (0.55; 1.33)	100.00	0.97 (0.63; 1.50)	100.00	0.75 (0.47; 1.19)	100.00
Highest (reference)									

*Note:* Model 2: Model 1 + behavioural factors; Model 3: Model 1 + material factors; Model 4: Model 1 + psychosocial factors and Model 5: Model 1 + behavioural factors + material factors + psychosocial factors. All models were adjusted for the ‘area of residence’, the ‘main language spoken at home’ and ‘PCGs' country of birth’. Bold = Significant ORs (95% CI does not include 1).

Abbreviations: OR, odds ratio; PCOR, percentage change in odds ratio.

**TABLE 3:** Association between PCGs' highest educational level/family income/family class/medical card status and young males'/females parent‐reported oral health adjusted for covariates (Wave 1), behavioural factors, material factors and psychosocial factors (logistic regression odds ratios (95% CI) for two teeth with dental fillings).

	Model 1	Model 2	PCOR	Model 3	PCOR	Model 4	PCOR	Model 5	PCOR
PCG education									
Females									
None or primary	**3.34 (1.99; 5.60)**	**3.41 (2.01; 5.78)**	−2.99	**2.92 (1.63; 5.24)**	17.95	**3.28 (1.87; 5.76)**	2.56	**3.88 (2.18; 6.89)**	−23.08
Secondary	**1.47 (1.12; 1.93)**	**1.48 (1.12; 1.95)**	−2.13	**1.52 (1.13; 2.04)**	−10.64	**1.49 (1.13; 1.97)**	−4.26	**1.68 (1.25; 2.25)**	−44.68
Tertiary (reference)									
Income quintiles									
Males									
Lowest	**1.82 (1.30; 2.55)**	**1.83 (1.29; 2.58)**	−1.22	**1.60 (1.07; 2.38)**	26.83	**1.58 (1.12; 2.24)**	29.27	**1.65 (1.12; 2.42)**	20.73
2nd	1.13 (0.80, 1.60)	1.13 (0.79; 1.62)	0.00	1.00 (0.66; 1.49)	100.00	0.94 (0.65; 1.36)	100.00	0.97 (0.65; 1.45)	100.00
3rd	1.34 (0.94; 1.90)	1.26 (0.88; 1.80)	23.53	1.18 (0.80; 1.74)	47.06	1.17 (0.82; 1.68)	50.00	1.12 (0.77; 1.63)	64.71
4th	1.25 (0.89; 1.76)	1.27 (0.90; 1.79)	−8.00	1.17 (0.82; 1.66)	32.00	1.12 (0.79; 1.59)	52.00	1.13 (0.79; 1.62)	48.00
Highest (reference)									
Females									
Lowest	**1.78 (1.26; 2.52)**	**1.88 (1.32; 2.69)**	−12.82	**1.87 (1.23; 2.84)**	−11.54	**1.76 (1.22; 2.53)**	2.56	**2.16 (1.45; 3.23)**	−48.72
2nd	**1.64 (1.17; 2.31)**	**1.69 (1.19; 2.40)**	−7.81	**1.73 (1.16; 2.59)**	−14.06	**1.63 (1.14; 2.33)**	1.56	**1.96 (1.32; 2.90)**	−50.00
3rd	**1.62 (1.16; 2.29)**	**1.70 (1.20; 2.42)**	−12.90	**1.79 (1.23; 2.62)**	−27.42	**1.60 (1.12; 2.27)**	3.23	**1.97 (1.35; 2.87)**	−56.45
4th	0.96 (0.66; 1.39)	0.97 (0.66; 1.41)	25.00	1.04 (0.70; 1.54)	100.00	0.95 (0.65; 1.40)	−25.00	1.07 (0.71; 1.59)	100.00
Highest (reference)									
Family class (occupation)									
Females									
Semi‐skilled/unskilled manual	**1.74 (1.29; 2.36)**	**1.77 (1.30; 2.41)**	−4.05	**1.51 (1.07; 2.12)**	31.08	**1.65 (1.21; 2.26)**	12.16	**1.56 (1.12; 2.18)**	24.32
Other non‐manual/skilled manual	1.19 (0.94; 1.50)	1.17 (0.92; 1.49)	10.53	1.14 (0.88; 1.49)	26.32	1.17 (0.91; 1.49)	10.53	1.17 (0.90; 1.52)	10.53
Professional managers (Reference)									
Medical card status									
Males									
Yes, full card	**1.65 (1.34; 2.05)**	**1.66 (1.34; 2.07)**	−1.54	**1.72 (1.29; 2.30)**	−10.77	**1.41 (1.11; 1.80)**	36.92	**1.47 (1.13; 1.93)**	27.69
Yes, doctor only card	0.60 (0.26; 1.40)	0.61 (0.26; 1.42)	2.50	0.61 (0.26; 1.43)	2.50	0.54 (0.23; 1.28)	−15.00	0.60 (0.25; 1.40)	0.00
Not covered (reference)									
Females									
Yes, full card	**1.41 (1.14; 1.74)**	**1.49 (1.20; 1.86)**	−19.51	1.26 (0.95; 1.67)	36.59	**1.41 (1.11; 1.78)**	0.00	**1.57 (1.23; 1.99)**	−39.02
Yes, doctor only card	1.43 (0.75; 2.73)	1.55 (0.80; 2.98)	−27.91	1.26 (0.64; 2.51)	39.53	1.37 (0.70; 2.68)	13.95	1.57 (0.79; 3.11)	−32.56
Not covered (reference)									

*Note:* Model 2: Model 1 + behavioural factors; Model 3: Model 1 + material factors; Model 4: Model 1 + psychosocial factors; and Model 5: Model 1 + behavioural factors + material factors + psychosocial factors. All models were adjusted for the ‘area of residence’, the ‘main language spoken at home’ and ‘PCGs' country of birth’. Bold = significant ORs (95% CI does not include 1).

Abbreviations: OR, odds ratio; PCOR, percentage change in odds ratio.

**TABLE 4:** Association between PCGs' highest educational level/family income/family class/medical card status and young males'/females parent‐reported oral health adjusted for covariates (Wave 1), behavioural factors, material factors and psychosocial factors (logistic regression odds ratios (95% CI) for three or more teeth with dental fillings).

	Model 1	Model 2	PCOR	Model 3	PCOR	Model 4	PCOR	Model 5	PCOR
PCG Education									
Females									
None or primary	**1.91 (1.07; 3.43)**	**1.87 (1.03; 3.40)**	4.40	1.81 (0.97; 3.38)	10.99	**2.11 (1.14; 3.89)**	−21.98	**1.93 (1.03; 3.60)**	−2.20
Secondary	1.28 (0.98; 1.67)	1.23 (0.94; 1.62)	17.86	1.15 (0.86; 1.53)	46.43	1.22 (0.93; 1.61)	21.43	1.21 (0.91; 1.61)	25.00
Tertiary (reference)									
Income quintiles									
Males									
Lowest	**1.58 (1.11; 2.25)**	**1.60 (1.11; 2.30)**	−3.45	1.26 (0.83; 1.92)	55.17	**1.52 (1.05; 2.21)**	10.34	1.25 (0.83; 1.88)	56.90
2nd	1.04 (0.72; 1.50)	1.07 (0.73; 1.56)	−75.00	0.88 (0.57; 1.34)	100.00	1.00 (0.68; 1.47)	100.00	0.89 (0.58; 1.35)	100.00
3rd	1.31 (0.91; 1.87)	1.32 (0.91; 1.90)	−3.23	1.22 (0.82; 1.82)	29.03	1.29 (0.88; 1.88)	6.45	1.18 (0.80; 1.75)	41.94
4th	1.37 (0.97; 1.94)	1.28 (0.89; 1.82)	24.32	1.32 (0.91; 1.90)	13.51	1.42 (0.99; 2.03)	−13.51	1.16 (0.80; 1.69)	56.76
Highest (reference)									
Females									
Lowest	**1.89 (1.34; 2.66)**	**1.70 (1.20; 2.43)**	21.35	1.40 (0.93; 2.13)	55.06	**1.66 (1.15; 2.39)**	25.84	1.50 (1.00; 2.23)	43.82
2nd	0.97 (0.68; 1.41)	0.96 (0.66; 1.40)	−33.33	0.76 (0.49; 1.17)	100.00	0.93 (0.63; 1.37)	100.00	0.84 (0.55; 1.29)	100.00
3rd	**1.42 (1.01; 2.01)**	**1.46 (1.02; 2.08)**	−9.52	1.24 (0.85; 1.82)	42.86	1.42 (0.99; 2.03)	0.00	**1.50 (1.02; 2.20)**	−19.05
4th	1.09 (0.76; 1.56)	1.04 (0.72; 1.51)	55.56	0.94 (0.64; 1.38)	100.00	1.00 (0.69; 1.47)	100.00	0.96 (0.64; 1.42)	100.00
Highest (reference)									
Family class (occupation)									
Females									
Semi‐skilled/unskilled manual	**1.70 (1.24; 2.33)**	**1.61 (1.16; 2.24)**	12.86	1.35 (0.94; 1.95)	50.00	**1.56 (1.11; 2.19)**	20.00	1.29 (0.90; 1.86)	58.57
Other non‐manual/skilled manual	**1.39 (1.10; 1.77)**	**1.31 (1.02; 1.67)**	20.51	1.29 (0.99; 1.70)	25.64	**1.32 (1.03; 1.70)**	17.95	1.19 (0.91; 1.55)	51.28
Professional managers (reference)									
Medical card status									
Males									
Yes, full card	**1.44 (1.16; 1.79)**	**1.43 (1.14; 1.80)**	2.27	1.39 (1.04; 0.85)	11.36	**1.34 (1.05; 1.71)**	22.73	1.30 (1.00; 1.70)	31.82
Yes, doctor only card	0.48 (0.19; 1.21)	0.51 (0.20; 1.28)	5.77	0.44 (0.17; 1.10)	−7.69	0.46 (0.18; 1.16)	−3.85	0.47 (0.18; 1.17)	−1.92
Not covered (Reference)									
Females									
Yes, full card	**1.62 (1.31; 2.00)**	**1.59 (1.27; 1.97)**	4.84	1.31 (0.98; 1.75)	50.00	**1.47 (1.16; 1.87)**	24.19	**1.41 (1.10; 1.81)**	33.87
Yes, doctor only card	1.26 (0.62; 2.54)	1.35 (0.66; 0.75)	−34.62	1.12 (0.53; 2.33)	53.85	0.87 (0.38; 1.99)	100.00	0.84 (0.36; 1.98)	100.00
Not covered (reference)									

*Note:* Model 2: Model 1 + behavioural factors; Model 3: Model 1 + material factors; Model 4: Model 1 + psychosocial factors; and Model 5: Model 1 + behavioural factors + material factors + psychosocial factors. All models were adjusted for the ‘area of residence’, the ‘main language spoken at home’ and ‘PCGs' country of birth’. Bold = significant ORs (95% CI does not include 1).

Abbreviations: OR, odds ratio; PCOR, percentage change in odds ratio.

### 3.2 Potential Mediators and Oral Health Outcomes; SES and Potential Mediators

The odds of having suboptimal SROH and dental fillings were generally higher among individuals in the unfavourable behavioural, material, or psychosocial groups than those in the favourable groups (Tables S5–S13 in File S1). The odds of having unfavourable behavioural, material and psychosocial outcomes were higher for the lower social groups when compared with the highest social groups (Tables S14–S25 in File S1).

### 3.3 Mediation Analysis

The significant predictor–outcome relationships identified among young males and females (refer to Section 3.1) were further investigated for mediation effects.

#### 3.3.1 Self‐Reported Oral Health

Among young males, adjustment of each set of behavioural, material and psychosocial factors reduced the odds of having suboptimal SROH for the lowest educational and income groups (97%–100%, 22%–69%, and 5%–56% respectively) (Table 2 and Tables S26 and S27 in File S1).

#### 3.3.2 Parent‐Reported Oral Health

For the lowest income and full medical card group males, the odds of having dental fillings were reduced after adjusting model 1 for behavioural factors (0%–2%), material (11%–55%) and psychosocial (10%–37%) factors (Tables 3 and 4 and Tables S30, S31, S34 and S35 in File S1). For young females in the lowest educational, income, occupational and full medical card groups, the odds of having dental fillings were reduced after adjusting for behavioural (0%–21%), material (11%–55%) and psychosocial (0%–26%) factors (Tables 3 and 4 and Tables S28–S35 in File S1).

Mediation analysis indicated that behavioural, material and psychosocial factors mediated the relationship between SES and oral health outcomes. Material factors emerged as the primary mediators, contributing most substantially to socio‐economic disparities in both SROH and parent‐reported number of dental fillings.

## 4 Discussion

This study presents the first comprehensive longitudinal analysis of socio‐economic oral health inequalities among Irish adolescents using nationally representative longitudinal data. The findings demonstrate significant socio‐economic disparities in both SROH and parent‐reported oral health outcomes (number of teeth with dental fillings). Adolescents from lower socio‐economic backgrounds at age 13 exhibited poorer oral health outcomes at age 17 compared with their more advantaged peers, consistent with previous research across various age groups and populations [9, 12, 15, 33, 41, 50, 52, 53, 54, 55, 56, 57]. These results align with earlier national children's oral health surveys in Ireland, which identified oral health inequalities associated with family occupational class and medical card status [37, 38].

The present analysis demonstrated significant gender‐specific socio‐economic inequalities in adolescent oral health, with different mediating pathways operating for different outcomes. Gender disparity in self‐perceived oral health and associated behaviours appears to be developmentally linked; as females age, they typically demonstrate greater initiative in seeking oral health information and adopting health‐promoting practices, factors that collectively contribute to enhanced oral health outcomes [42, 58, 59].

The current research findings provide supporting evidence that behavioural, material and psychosocial factors are important factors contributing to educational and income‐related SROH disparities in young males. While material factors contributed most to the explanation of social disparities in parent‐reported dental filling outcomes in both males and females, behavioural and psychosocial factors played a limited or no role in accounting for these disparities. The interpretation of these results in the context of existing literature presents challenges, as this study represents the first longitudinal investigation examining the concurrent role of behavioural, material and psychosocial factors in explaining both self and parent‐reported oral health inequalities among adolescents. Recent studies across different regions have demonstrated limited success in explaining oral health disparities through traditional factors. Research from California demonstrated that socio‐economic disparities in adolescent oral health could only be partially explained by health behaviours, genetic factors and access to dental care [53]. However, research in Pennsylvania demonstrated that selected oral health behaviours and preventive interventions did not account for socio‐economic disparities in caries experience in adolescents [13]. Likewise, research involving Iranian adolescents concluded that behavioural and psychological factors explained only a small portion of the relationships between SES indicators and self‐reported oral health outcomes among Iranian adolescents [50]. Evidence from comparable studies in adults is inconclusive; some studies reported little or no role for behavioural factors [7, 12, 40], while others suggested a substantial role in explaining socio‐economic oral health inequalities [8, 9, 11, 15, 44, 54, 60]. Evidence from general health research suggested that the material factors substantially explained socio‐economic differences in self‐rated general health among children, adolescents and adults [17, 22, 43, 61].

It is important to note that different oral health measures capture distinct aspects of oral well‐being [15, 58]. Self‐reported oral health provides a subjective assessment of current status, often reflecting immediate concerns such as pain or functional limitations [58]. In contrast, parent‐reported dental fillings indicate historical dental issues and may be influenced by various factors, including parental beliefs and healthcare accessibility [33]. This distinction helps explain why explanatory factors contribute differently to socio‐economic disparities depending on the specific oral health outcome being studied.

The study analysis revealed that while behavioural factors significantly predicted oral health status, they played a limited role in explaining socio‐economic inequalities. Instead, material circumstances emerged as the primary driver of these disparities. This finding has substantial implications for Ireland's national oral health policy, suggesting that while behavioural interventions remain valuable, addressing material inequalities should be prioritised to achieve meaningful improvements across socio‐economic groups.

The current research benefits from several methodological strengths. The large, nationally representative sample provided robust statistical power for gender‐specific analyses. The longitudinal design enabled the examination of temporal relationships between socio‐economic factors and oral health outcomes. Additionally, the comprehensive range of factors investigated allowed for a thorough exploration of potential pathways explaining oral health inequalities. The study findings should be interpreted cautiously as there were some methodological considerations and limitations. The SROH outcome variable and most material and psychosocial factors were dichotomised to facilitate analysis and interpretation. While the cut‐points were based on previously published literature [15, 17], consistent with common practice in oral epidemiological research [62], this approach has several limitations [63, 64]. Dichotomisation may result in a considerable loss of information and potentially underestimate between‐group variations [63, 65]. This analytical strategy also reduces statistical power and may obscure non‐linear relationships or gradient effects that could provide more nuanced insights into the associations examined [63, 64]. The number of dental fillings may be influenced by various factors, including parental beliefs, dental service accessibility and affordability [33]. The material deprivation measures used in the study should be interpreted in the context of the timing of ‘The Great Recession’. Wave 2 data were collected between August 2011 and March 2012, when the children were 13 years old, corresponding to the recession's deepest point [66]. Furthermore, the use of a single time‐point data for time‐varying factors, such as sugar consumption, income and fluoride exposure, rather than repeated measures, may not fully capture the dynamic nature of these influences. A significant limitation of the present analysis relates to the assessment of fluoride exposure through community water fluoridation (CWF). While the authors used the area of residence as a proxy for CWF status, with urban areas typically receiving fluoridated water and rural areas remaining non‐fluoridated, this approach may not fully capture individual fluoride exposure levels [67]. Although authors could account for the widespread use of fluoridated toothpaste in Ireland (with over 95% market penetration since 2002), the variability in lifetime exposure to fluoridated water likely influences the number of dental fillings observed among participants [38]. Furthermore, the persistence of oral health inequalities after adjustment of behavioural, material and psychosocial factors suggests other explanatory factors that have not been measured. These may include significant life events, individual oral health beliefs, dental anxiety, cultural values and access to health‐related support networks [18]. The temporal relationship between these explanatory factors and oral health outcomes also presents a methodological challenge, as the exact timing of exposures and outcomes could not be definitively established in the present study design. An important methodological limitation was related to the mediation framework used in this study. The primary mediation analysis used the Baron and Kenny framework [49] to enable comparison with previously published literature [13, 50]. However, this approach has limitations, including the inability to rigorously adjust for mediator‐outcome confounding and the lack of formal inference for indirect effects [68, 69, 70]. Supplementary analyses using bootstrap and causal mediation methods [70] (Tables S1–S4 in File S2) revealed substantially weaker effects, suggesting that the current study estimates may have overestimated mediation due to unmeasured confounding and should be interpreted as upper bounds rather than precise causal effects. The comparisons of these mediation approaches with the primary approach used in this study have been outlined in (Tables S5–S7 in File S2). The methodological comparisons highlighted the sensitivity of mediation findings to analytic choices and the importance of cautious interpretation. Finally, the validity of self‐reported measures used in the GUI surveys was unclear and may be prone to recall, reporting and social desirability bias [66].

In agreement with previous publications [14, 15, 16, 19, 25, 50, 57], the present study findings suggest several priorities for future research. These include examining the interaction between area‐based and individual socio‐economic measures, exploring structural health determinants through longitudinal studies, and investigating barriers to healthy oral health behaviours. Additionally, linking administrative data with longitudinal surveys could provide valuable insights for developing more effective public health interventions. These research directions, combined with the study findings on the importance of material circumstances in driving oral health inequalities, suggest that future interventions should focus on enhancing the material circumstances of families with lower SES while maintaining existing behavioural intervention programmes. This dual approach acknowledges both the complex nature of oral health outcomes and the multifaceted interventions required to address them effectively [8, 15].

The present investigation demonstrated socio‐economic disparities in oral health among Irish adolescents. Males demonstrated associations between SES and both SROH and dental fillings, while females showed associations between SES and dental fillings. Behavioural, material and psychosocial factors contributed to explaining these oral health disparities, and material factors emerged as the primary pathway explaining these inequalities. While CWF has proven effective in reducing oral health inequalities in Ireland, the study findings suggest that additional improvements could be achieved by implementing interventions targeting material deprivation, combined with gender‐specific approaches.

## Author Contributions

Conceptualisation: Vinay Sharma, Michael O'Sullivan, Michael Crowe, Aifric O'Sullivan, Oscar Cassetti and Lewis Winning. Methodology: Vinay Sharma, Michael O'Sullivan, Michael Crowe, Bahman Honari. Data acquisition and statistical analysis: Vinay Sharma, Michael O'Sullivan, Michael Crowe and Bahman Honari. Interpretation of findings: Vinay Sharma, Michael O'Sullivan, Michael Crowe and Lewis Winning. Drafting and critically reviewing the manuscript: Vinay Sharma, Michael O'Sullivan, Michael Crowe, Aifric O'Sullivan, Oscar Cassetti, Bahman Honari and Lewis Winning. Project administration: Michael O'Sullivan. All authors have read and approved the final manuscript.

## Funding

This study was completed as a part of a Doctor of Philosophy (PhD) degree by VS supported by the Dublin Dental University Hospital and Trinity College Dublin, Ireland (1252 scholarship).

## Ethics Statement

The authors have nothing to report.

## Consent

The authors have nothing to report.

## Conflicts of Interest

The authors declare no conflicts of interest.

## Supporting information


**Table S1:** Transformations of study variables' response categories and their value labels.
**Table S2:** Crude (unadjusted) associations between socio‐economic measures (SES) measures (PCGs' highest educational level, family income, family occupational class and study participants' medical card status) at 13 years of age and young persons' self‐reported oral health (self‐rated oral health, SROH) at 17/18 years for the complete sample and by gender.
**Table S3:** Crude (unadjusted) associations between socio‐economic status (SES) measures (PCGs' highest educational level, family income, family occupational class and study participants' medical card status) at 13 years of age and young persons' parent‐reported oral health (number of permanent teeth with dental fillings) at 17/18 years of age.
**Table S4:** Crude (unadjusted) associations between socio‐economic status (SES) measures (PCGs' highest educational level, family income, family occupational class and study participants' medical card status) at 13 years of age and young persons' parent‐reported oral health (number of permanent teeth with dental fillings) at 17/18 years of age for young males and females.
**Table S5:** Crude (unadjusted) associations between behavioural factors (daily tooth brushing frequency, dentist visit behaviour, sugary foods intake and sugary drinks intake) at 13 years of age and young persons' parent‐reported oral health (number of teeth with dental fillings) at 17/18 years of age.
**Table S6:** Crude (unadjusted) associations between behavioural factors (daily tooth brushing frequency, dentist visit behaviour, sugary foods intake and sugary drinks intake) at 13 years of age and young persons' parent‐reported oral health (number of teeth with dental fillings) at 17/18 years of age and by gender.
**Table S7:** Crude (unadjusted) associations between behavioural factors (daily tooth brushing frequency, dentist visit behaviour, sugary foods intake and sugary drinks intake) at 13 years of age and young persons' self‐reported oral health (self‐rated oral health, SROH) at 17/18 years of age and by gender.
**Table S8:** Crude (unadjusted) associations between material factors (financial difficulties, DEIS school status, house ownership and private medical insurance cover) at 13 years of age and young persons' parent‐reported oral health (number of teeth with dental fillings) at 17/18 years of age.
**Table S9:** Crude (unadjusted) associations between material factors (financial difficulties, DEIS school status, house ownership and private medical insurance cover) at 13 years of age and young persons' parent‐reported oral health (number of teeth with dental fillings) at 17/18 years of age and by gender.
**Table S10:** Crude (unadjusted) associations between material factors (financial difficulties, DEIS school status, house ownership and private medical insurance cover) at 13 years of age and young persons' self‐reported oral health (self‐reported oral health) at 17/18 years of age and by gender.
**Table S11:** Crude (unadjusted) associations between psychosocial factors (PCG depression status, family structure, job stress, and PCG mean parental stress scale score) at 13 years of age and young persons' parent‐reported oral health (number of teeth with dental fillings) at 17/18 years of age.
**Table S12:** Crude (unadjusted) associations between psychosocial factors (PCG depression status, family structure, job stress and PCG mean parental stress scale score) at 13 years of age and young persons' parent‐reported oral health (number of teeth with dental fillings) at 17/18 years of age and by gender.
**Table S13:** Crude (unadjusted) associations between psychosocial factors (PCG depression status, family structure, job stress and PCG mean parental stress scale score) at 13 years of age and young persons' parent‐reported oral health (number of teeth with dental fillings) at 17/18 years of age and by gender.
**Table S14:** Association between SES components and toothbrushing.
**Table S15:** Association between SES components and dentist visit behaviour.
**Table S16:** Association between SES components and sugary foods intake.
**Table S17:** Association between SES components and sugary drinks intake.
**Table S18:** Association between SES components and DEIS school status.
**Table S19:** Association between SES components and financial difficulties.
**Table S20:** Association between SES components and private medical insurance.
**Table S21:** Association between SES components and private house ownership.
**Table S22:** Association between SES components and family composition.
**Table S23:** Association between SES components and job stress.
**Table S24:** Association between SES components and depression status.
**Table S25:** Association between SES components and parental stress scale.
**Table S26:** Association between PCGs' highest educational level and young males' self‐reported oral health (self‐rated oral health) adjusted for covariates (Wave 1), behavioural factors, material factors and psychosocial factors (logistic regression odds ratios (95% CI) for self‐rated suboptimal oral health). Model 2: Model 1 + behavioural factors; Model 3: Model 1 + material factors; Model 4: Model 1 + psychosocial factors and Model 5: Model 1 + behavioural factors + material factors + psychosocial factors. All models were adjusted for the ‘area of residence’, the ‘main language spoken at home’ and ‘PCGs' country of birth’. OR: Odds ratio; NA (not applicable) refers to a variable that was excluded from the model using stepwise backward selection.
**Table S27:** Association between household/family income and young males' self‐reported oral health (self‐rated oral health) adjusted for covariates (Wave 1), behavioural factors, material factors and psychosocial factors (logistic regression odds ratios (95% CI) for self‐rated suboptimal oral health). Model 2: Model 1 + behavioural factors; Model 3: Model 1 + material factors; Model 4: Model 1 + psychosocial factors and Model 5: Model 1 + behavioural factors + material factors + psychosocial factors. All models were adjusted for the ‘area of residence’, the ‘main language spoken at home’ and ‘PCGs' country of birth’. OR, odds ratio; NA (not applicable) refers to a variable that was excluded from the model using stepwise backward selection.
**Table S28:** Association between PCGs' highest educational level and young females' parent‐reported oral health (number of teeth with dental fillings) adjusted for covariates (Wave 1), behavioural factors, material factors and psychosocial factors (logistic regression odds ratios (95% CI) for having two teeth with dental fillings). Model 2: Model 1 + behavioural factors; Model 3: Model 1 + material factors; Model 4: Model 1 + psychosocial factors and Model 5: Model 1 + behavioural factors + material factors + psychosocial factors. All models were adjusted for the ‘area of residence’, the ‘main language spoken at home’ and ‘PCGs' country of birth’. OR, odds ratio; NA (not applicable) refers to a variable that was excluded from the model using stepwise backward selection.
**Table S29:** Association between PCGs' highest educational level and young females' parent‐reported oral health (number of teeth with dental fillings) adjusted for covariates (Wave 1), behavioural factors, material factors and psychosocial factors (logistic regression odds ratios (95% CI) for having three or more teeth with dental fillings). Model 2: Model 1 + behavioural factors; Model 3: Model 1 + material factors; Model 4: Model 1 + psychosocial factors and Model 5: Model 1 + behavioural factors + material factors + psychosocial factors. All models were adjusted for the ‘area of residence’, the ‘main language spoken at home’ and ‘PCGs' country of birth’. OR, odds ratio; NA (not applicable) refers to a variable that was excluded from the model using stepwise backward selection.
**Table S30:** Association between household/family income and young persons' parent‐reported oral health (number of teeth with dental fillings) adjusted for covariates (Wave 1), behavioural factors, material factors and psychosocial factors (logistic regression odds ratios (95% CI) for having two teeth with dental fillings). Model 2: Model 1 + behavioural factors; Model 3: Model 1 + material factors; Model 4: Model 1 + psychosocial factors and Model 5: Model 1 + behavioural factors + material factors + psychosocial factors. All models were adjusted for the ‘area of residence’, the ‘main language spoken at home’ and ‘PCGs' country of birth’. OR, odds ratio; NA (not applicable) refers to a variable that was excluded from the model using stepwise backward selection.
**Table S31:** Association between household/family income and young persons' parent‐reported oral health (number of teeth with dental fillings) adjusted for covariates (Wave 1), behavioural factors, material factors and psychosocial factors (logistic regression odds ratios (95% CI) for having three or more teeth with dental fillings). Model 2: Model 1 + behavioural factors; Model 3: Model 1 + material factors; Model 4: Model 1 + psychosocial factors and Model 5: Model 1 + behavioural factors + material factors + psychosocial factors. All models were adjusted for the ‘area of residence’, the ‘main language spoken at home’ and ‘PCGs' country of birth’. OR, odds ratio; NA (not applicable) refers to a variable that was excluded from the model using stepwise backward selection.
**Table S32:** Association between family occupational class and young females' parent‐reported oral health (number of teeth with dental fillings) adjusted for covariates (Wave 1), behavioural factors, material factors and psychosocial factors (logistic regression odds ratios (95% CI) for having two teeth with dental fillings). Model 2: Model 1 + behavioural factors; Model 3: Model 1 + material factors; Model 4: Model 1 + Psychosocial factors and Model 5: Model 1 + behavioural factors + material factors + psychosocial factors. All models were adjusted for the ‘area of residence’, the ‘main language spoken at home’ and ‘PCGs' country of birth’. OR, odds ratio; NA (not applicable) refers to a variable that was excluded from the model using stepwise backward selection.
**Table S33:** Association between family occupational class and young females' parent‐reported oral health (number of teeth with dental fillings) adjusted for covariates (Wave 1), behavioural factors, material factors and psychosocial factors (logistic regression odds ratios (95% CI) for having three teeth with dental fillings). Model 2: Model 1 + behavioural factors; Model 3: Model 1 + material factors; Model 4: Model 1 + psychosocial factors and Model 5: Model 1 + behavioural factors + material factors + psychosocial factors. All models were adjusted for the ‘area of residence’, the ‘main language spoken at home’ and ‘PCGs' country of birth’. OR, odds ratio; NA (not applicable) refers to a variable that was excluded from the model using stepwise backward selection.
**Table S34:** Association between family medical card status and young persons' parent‐reported oral health (number of teeth with dental fillings) adjusted for covariates (Wave 1), behavioural factors, material factors and psychosocial factors (logistic regression odds ratios (95% CI) for having two teeth with dental fillings). Model 2: Model 1 + behavioural factors; Model 3: Model 1 + material factors; Model 4: Model 1 + psychosocial factors and Model 5: Model 1 + behavioural factors + material factors + psychosocial factors. All models were adjusted for the ‘area of residence’, the ‘main language spoken at home’ and ‘PCGs' country of birth’. OR, odds ratio; NA (not applicable) refers to a variable that was excluded from the model using stepwise backward selection.
**Table S35:** Association between family medical card status and young persons' parent‐reported oral health (number of teeth with dental fillings) adjusted for covariates (Wave 1), behavioural factors, material factors and psychosocial factors (logistic regression odds ratios (95% CI) for having three or more teeth with dental fillings). Model 2: Model 1 + behavioural factors; Model 3: Model 1 + material factors; Model 4: Model 1 + psychosocial factors and Model 5: Model 1 + behavioural factors + material factors + psychosocial factors. All models were adjusted for the ‘area of residence’, the ‘main language spoken at home’ and ‘PCGs' country of birth’. OR, odds ratio; NA (not applicable) refers to a variable that was excluded from the model using stepwise backward selection.

**Table S1:** Association between PCGs' highest educational level/family income and young males' self‐reported oral health (self‐rated oral health) adjusted for covariates (Wave 1), behavioural factors, material factors and psychosocial factors (logistic regression odds ratios for self‐rated suboptimal oral health). Model 2: Model 1 + behavioural factors; Model 3: Model 1 + material factors; Model 4: Model 1 + psychosocial factors and Model 5: Model 1 + behavioural factors + material factors + psychosocial factors. All models were adjusted for the ‘area of residence’, the ‘main language spoken at home’ and ‘PCGs' country of birth’. CI, confidence intervals; direct effect, outcome ~ exposure + mediators + covariates; Indirect effect, total effect–direct effect; OR, odds ratio; PCOR, percentage change in odds ratio; Percentage mediated, indirect effect/total effect; total effect: outcome ~ exposure + covariates.
**Table S2:** Association between PCGs' highest educational level/family income/family class/medical card status and young males'/females parent‐reported oral health adjusted for covariates (Wave 1), behavioural factors, material factors and psychosocial factors (logistic regression odds ratios for two teeth with dental fillings and three or more teeth with dental fillings outcomes). Model 2: Model 1 + behavioural factors; Model 3: Model 1 + material factors; Model 4: Model 1 + psychosocial factors; and Model 5: Model 1 + behavioural factors + material factors + psychosocial factors. All models were adjusted for the ‘area of residence’, the ‘main language spoken at home’ and ‘PCGs' country of birth’. CI, confidence intervals; Direct effect, outcome ~ exposure + mediators + covariates; Indirect effect, total effect–direct effect; OR, odds ratio; PCOR, percentage change in odds ratio; percentage mediated, indirect effect/total effect; total effect, Outcome ~ exposure + covariates.
**Table S3:** Association between PCGs' highest educational level/family income and young males' self‐reported oral health (self‐rated oral health) adjusted for covariates (Wave 1), behavioural factors, material factors and psychosocial factors (logistic regression odds ratios for self‐rated suboptimal oral health) (Causal mediation approach, simulations = 1000). Model 2: Model 1 + behavioural factors; Model 3: Model 1 + material factors; Model 4: Model 1 + Psychosocial factors and Model 5: Model 1 + behavioural factors + material factors + Psychosocial factors. All models were adjusted for the ‘area of residence’, the ‘main language spoken at home’ and ‘PCGs' country of birth’. CI, confidence intervals; direct effect, outcome ~ exposure + mediators + covariates; indirect effect, total effect–direct effect; NDE, natural direct effect; NIE, natural indirect effect; OR, odds ratio; PCOR, percentage change in odds ratio; percentage mediated, indirect effect/total effect; total effect, outcome ~ exposure + covariates.
**Table S4:** Association between PCGs' highest educational level/family income/family class/medical card status and young males'/females parent‐reported oral health adjusted for covariates (Wave 1), behavioural factors, material factors and psychosocial factors (logistic regression odds ratios for two teeth with dental fillings and three or more teeth with dental fillings outcomes (Causal mediation approach, simulations = 1000)). Model 2: Model 1 + behavioural factors; Model 3: Model 1 + material factors; Model 4: Model 1 + psychosocial factors; and Model 5: Model 1 + behavioural factors + material factors + psychosocial factors. All models were adjusted for the ‘area of residence’, the ‘main language spoken at home’ and ‘PCGs' country of birth’. CI, confidence intervals; direct effect, outcome ~ exposure + mediators + covariates; indirect effect: total effect–direct effect; OR, odds ratio; PCOR, percentage change in odds ratio; percentage mediated, indirect effect/total effect; total effect, Outcome ~ exposure + covariates.
**Table S5:** Comparison of mediation estimates across three analytic approaches for potential mediation of behavioural/material/psychosocial factors in the relationship between self‐reported oral health and PCG's highest educational level and Family income quintiles. PCOR: Percentage change in odds ratio.
**Table S6:** Comparison of mediation estimates across three analytic approaches for potential mediation of behavioural/material/psychosocial factors in the relationship between parent‐reported oral health (Outcome: Two teeth with dental fillings) and PCG's highest educational level/Family income quintiles/Family occupation/Medical card status among males and females. PCOR: Percentage change in odds ratio.
**Table S7:** Comparison of mediation estimates across three analytic approaches for potential mediation of behavioural/material/psychosocial factors in the relationship between parent‐reported oral health (Outcome: Three or more teeth with dental fillings) and PCG's highest educational level/Family income quintiles/Family occupation/Medical card status among males and females. PCOR, percentage change in odds ratio.

## Data Availability

The data that support the findings of this study are openly available in the Irish Social Science Data Archive (ISSDA) at https://www.ucd.ie/issda/accessdata/issdadatasets/.
