# Supplementary material for: Socio‐Economic Differences in the Oral Health of Irish Adolescents: The Potential Role of Behavioural, Material and Psychosocial Factors
Source: Community Dent Oral Epidemiol. 2025 Dec 7;54(3):316–32. doi: 10.1111/cdoe.70043 (PMC13146151; doi:10.1111/cdoe.70043)
Supplement: Supplementary file 1 — Table S1: Transformations of study variables' response categories and their value labels. Table S2: Crude (unadjusted) associations between socio‐economic measures (SES) measures (PCGs' highest educational level, family income, family occupational class and study participants' medical card status) at 13 years of age and young persons' self‐reported oral health (self‐rated oral health, SROH) at 17/18 years for the complete sample and by gender. Table S3: Crude (unadjusted) associations between socio‐economic status (SES) measures (PCGs' highest educational level, family income, family occupational class and study participants' medical card status) at 13 years of age and young persons' parent‐reported oral health (number of permanent teeth with dental fillings) at 17/18 years of age. Table S4: Crude (unadjusted) associations between socio‐economic status (SES) measures (PCGs' highest educational level, family income, family occupational class and study participants' medical card status) at 13 years of age and young persons' parent‐reported oral health (number of permanent teeth with dental fillings) at 17/18 years of age for young males and females. Table S5: Crude (unadjusted) associations between behavioural factors (daily tooth brushing frequency, dentist visit behaviour, sugary foods intake and sugary drinks intake) at 13 years of age and young persons' parent‐reported oral health (number of teeth with dental fillings) at 17/18 years of age. Table S6: Crude (unadjusted) associations between behavioural factors (daily tooth brushing frequency, dentist visit behaviour, sugary foods intake and sugary drinks intake) at 13 years of age and young persons' parent‐reported oral health (number of teeth with dental fillings) at 17/18 years of age and by gender. Table S7: Crude (unadjusted) associations between behavioural factors (daily tooth brushing frequency, dentist visit behaviour, sugary foods intake and sugary drinks intake) at 13 years of age and young persons' self [file CDOE-54-316-s002.docx]

Table S1 Transformations of study variables’ response categories and their value labels.

| Variable | Original | | Transformed |
| --- | --- | --- | --- |
| Covariates, Wave 1 | | | |
|  | **Value** | **Response levels** | **Response levels (value)** |
| Region | 1.00 | Urban | Urban (2.00) |
|  | 2.00 | Rural | Rural (1.00) |
| Gender | 1.00 | Male | Male (1.00) |
|  | 2.00 | Female | Female (2.00) |
|  | 8.00 | Refusal | Missing (9.00) |
|  | 9.00 | Don’t know |  |
| Main language spoken at home | 1.00 | English | English (3.00) |
|  | 2.00 | Irish | Irish (2.00) |
|  | 3.00 | Other | Other (1.00) |
| PCG born in Ireland | 1.00 | Yes | Yes (2.00) |
|  | 2.00 | No | No (1.00) |
|  | 8.00 | Refusal | Missing (999.00) |
|  | 9.00 | Don’t know |  |
| Socioeconomic variables, Wave 2 | | | |
| Family occupational class | 1.00 | Professional Managers | Professional managers (3.00) |
|  | 2.00 | Managerial and Technical |  |
|  | 3.00 | Non-manual | Other non-manual/skilled manual (2.00) |
|  | 4.00 | Skilled manual |  |
|  | 5.00 | Semi-skilled | Semi-skilled/unskilled manual (1.00) |
|  | 6.00 | Unskilled |  |
|  | 7.00 | All other gainfully occupied and unknown | Missing (999.00) |
|  | 666.00 | Validity no social class |  |
|  | 777.00 | Mum and dad not resident |  |
|  | 888.00 | Dad figure not resident |  |
| Equivalised household annual income-Quintiles | 1.00 | Lowest | Lowest (1.00) |
|  | 2.00 | 2^nd^ | 2^nd^ (2.00) |
|  | 3.00 | 3^rd^ | 3^rd^ (3.00) |
|  | 4.00 | 4^th^ | 4^th^ (4.00) |
|  | 5.00 | Highest | Highest (5.00) |

Table S1 Continued….

| Variable | Original | | Transformed |
| --- | --- | --- | --- |
|  | **Value** | **Response levels** | **Response levels (value)** |
| PCG’s highest educational qualification level | 1.00 | None or primary | Primary or none (1.00) |
|  | 2.00 | Lower secondary | Secondary (2.00) |
|  | 3.00 | Higher secondary |  |
|  | 4.00 | TechVoc/UpperSec +Tec/Voc |  |
|  | 5.00 | Non Degree |  |
|  | 6.00 | Primary degree | Tertiary (3.00) |
|  | 7.00 | Postgrad degree |  |
|  | 98.00 | Refusal | Missing (999.00) |
|  | 99.00 | Don’t know |  |
| Study participants’ medical card status | 1.00 | Yes, full card | Yes, full card (1.00) |
|  | 2.00 | Yes, doctor only card | Yes, doctor only card (2.00) |
|  | 3.00 | Not covered | Not covered (3.00) |
|  | 8.00 | Refusal | Missing (999.00) |
|  | 9.00 | Don’t know |  |
| Oral Health Behaviours, Wave 2 | | | |
| Study child brushing their teeth at least once daily | 1.00 | More than twice a day | Twice or more than twice a day (2.00) |
|  | 2.00 | Twice a day |  |
|  | 3.00 | Once a day | Once a day (1.00) |
|  | 4.00 | Less than once a day | Less than once a day/Rarely/Not at all (0.00) |
|  | 5.00 | Rarely/Not at all |  |
|  | 6.00 | Not at all |  |
|  | 8.00 | Refusal | Missing (999.00) |
|  | 9.00 | Don’t know |  |
| Study child’s dentist visit behaviour | 1.00 | At least once a year | At least once a year (3.00) |
|  | 2.00 | Once every two years | Occasional visit (2.00) |
|  | 3.00 | Once every three years |  |
|  | 4.00 | Only when there is a problem | Problem visit/Never/Almost Never (1.00) |
|  | 5.00 | Never/almost never |  |
|  | 8.00 | Refusal | Missing (999.00) |
|  | 9.00 | Don’t know |  |
| Study child’s frequency of consumption of sugary foods (Biscuits, doughnuts, cake, pie or chocolate) | 1.00 | Once | Once (2.00) |
|  | 2.00 | More than once | More than once (1.00) |
|  | 3.00 | Not at all | Not at all (3.00) |
|  | 8.00 | Refusal | Missing (9.00) |
|  | 9.00 | Don’t know |  |

Table S1 Continued….

| Variable | Original | | Transformed |
| --- | --- | --- | --- |
|  | **Value** | **Response levels** | **Response levels (value)** |
| Study child’s frequency of consumption of sugary drinks (Soft drinks/minerals/cordial/squash (non-diet) | 1.00 | Once | Once (2.00) |
|  | 2.00 | More than once | More than once (1.00) |
|  | 3.00 | Not at all | Not at all (3.00) |
|  | 8.00 | Refusal | Missing (9.00) |
|  | 9.00 | Don’t know |  |
| Material circumstances, Wave 2 | | | |
| Degree of ease or difficulty of making ends meet | 1.00 | With great difficulty | With difficulty (1.00) |
|  | 2.00 | With difficulty |  |
|  | 3.00 | With some difficulty |  |
|  | 4.00 | Fairly easily | No difficulty (0.00) |
|  | 5.00 | Easily |  |
|  | 6.00 | Very easily |  |
|  | 8.00 | Refusal | Missing (999.00) |
|  | 9.00 | Don’t know |  |
| Private medical insurance | 1.00 | Yes | Yes (2.00) |
|  | 2.00 | No | No (1.00) |
|  | 8.00 | Refusal | Missing (999.00) |
|  | 9.00 | Don’t know |  |
| House ownership | 1.00 | Owner occupied (with/without mortgage) | Owner (2.00) |
|  | 2.00 | Being purchased from a local authority |  |
|  | 3.00 | Rented from a local authority | Renters/Others/Rent free (1.00) |
|  | 4.00 | Rented from a voluntary body |  |
|  | 5.00 | Rented from a private landlord |  |
|  | 6.00 | Living with and paying rent to parents |  |
|  | 7.00 | Occupied rent free-Parents or Job |  |
|  | 8.00 | Occupied rent free of rent from job |  |
|  | 9.00 | Other |  |
|  | 98.00 | Refusal | Missing (999.00) |
|  | 99.00 | Don’t know |  |

Table S1 Continued….

| Variable | Original | | Transformed |
| --- | --- | --- | --- |
|  | **Value** | **Response levels** | **Response levels (value)** |
| DEIS school status | 1.00 | Yes | Yes (1.00) |
|  | 2.00 | No | No (2.00) |
|  | 8.00 | Refusal | Missing (999.00) |
|  | 9.00 | Don’t know |  |
| Psychosocial factors, Wave 2 | | | |
| Family structure | 1.00 | Single parent 1 or 2 children | Single parent family (1.00) |
|  | 2.00 | Single parent 3 or more children | Single parent family (1.00) |
|  | 3.00 | Couple 1 or 2 children | Couple family (2.00) |
|  | 4.00 | Couple 3 or more children | Couple family (2.00) |
| PCG depression status | 1.00 | Depressed | Depressed (1.00) |
|  | 0.00 | Not depressed | Not depressed (2.00) |
| PCG Job stress | 1.00 | Very | Stressed (1.00) |
|  | 2.00 | Fairly |  |
|  | 3.00 | Not very | Not stressed (2.00) |
|  | 4.00 | Not at all |  |
|  | 8.00 | Refusal | Missing (999.00) |
|  | 9.00 | Don’t know |  |
| Oral health outcomes, Wave 3 | | | |
| Young persons’ self-reported oral health (Self-rated oral health, SROH) | 1.00 | Excellent | Optimal oral health (1.00) |
|  | 2.00 | Very good |  |
|  | 3.00 | Good |  |
|  | 4.00 | Fair | Sub-optimal health (2.00) |
|  | 5.00 | Poor |  |
|  | 8.00 | Refusal | Missing (999.00) |
|  | 9.00 | Don’t know |  |
| Parent-reported young persons’ oral health (Number of permanent teeth with dental fillings) | 0.00 | None | None (0.00) |
|  | 1.00 | One | One (1.00) |
|  | 2.00 | Two | Two (2.00) |
|  | 3.00 | Three or more | Three or more (3.00) |
|  | 8.00 | Refusal | Missing (999.00) |
|  | 9.00 | Don’t know |  |

Table S2 Crude (unadjusted) associations between socioeconomic measures (SES) measures (PCGs’ highest educational level, family income, family occupational class and study participants’ medical card status) at 13 years of age and young persons’ self-reported oral health (self-rated oral health, SROH) at 17/18 years for the complete sample and by gender.

|  |  | **Complete sample**  **Self-rated oral health** | | **Males**  **Self-rated oral health** | | **Females**  **Self-rated oral health** | |
| --- | --- | --- | --- | --- | --- | --- | --- |
|  |  | **Optimal**  **n (%)** | **Suboptimal**  **OR (95% CI)**  **n (%)** | **Optimal**  **n (%)** | **Suboptimal**  **OR (95% CI)**  **n (%)** | **Optimal**  **n (%)** | **Suboptimal**  **OR (95% CI)**  **n (%)** |
| Primary care giver’s highest level of education  (n = 6033  M = 3081  F = 2951) | None or primary | 184 (86.4) | **2.16 (1.38; 3.38)*****  29 (13.6) | 84 (82.4) | **2.52 (1.41; 4.49)****  18 (17.6) | 100 (90.1) | 1.86 (0.89; 3.87)  11 (9.9) |
|  | Secondary | 4222 (92.8) | 1.07 (0.84; 1.38)  330 (7.2) | 2052 (89.5) | 1.35 (0.99; 1.84)  241 (10.5) | 2170 (96.1) | 0.72 (0.47; 1.10)  88 (3.9) |
|  | Tertiary  (Reference) | 1182 (93.2) | 86 (6.8) | 631 (92.0) | 55 (8.0) | 551 (94.7) | 31 (5.3) |
| Family income – quintiles  (n = 6033  M = 2852  F = 2730) | Lowest | 1053 (91.6) | **1.55 (1.11; 2.176)***  97 (8.4) | 502 (87.6) | **1.59 (1.07; 2.35)***  71 (12.4) | 551 (95.5) | 1.74 (0.88; 3.43)  26 (4.5) |
|  | 2^nd^ | 1021 (90.8) | **1.71 (1.23; 2.39)****  104 (9.2) | 484 (86.3) | **1.79 (1.21; 2.64)****  77 (13.7) | 536 (95.2) | 1.83 (0.93; 3.60)  27 (4.8) |
|  | 3^rd^ | 1043 (93.5) | 1.18 (0.83; 1.69)  73 (6.5) | 468 (90.5) | 1.18 (0.77; 1.80)  49 (9.5) | 575 (96.0) | 1.52 0.76; 3.02)  24 (4.0) |
|  | 4^th^ | 1064 (92.9) | 1.29 (0.91; 1.82)  81 (7.1) | 587 (91.4) | 1.05 (0.69; 1.58)  55 (8.6) | 477 (94.8) | 2.03 (1.03; 3.99)  26 (5.2) |
|  | Highest  (Reference) | 987 (94.4) | 58 (5.6) | 513 (91.8) | 46 (8.2) | 475 (97.3) | 13 (2.7) |
| Family class (Occupation)  (n = 6033  M = 2828  F = 2646) | Semi-skilled/ Unskilled manual | 687 (92.8) | 1.06 (0.77; 1.46)  53 (7.2) | 302 (88.6) | 1.31 (0.90; 1.91)  39 (11.4) | 385 (96.5) | 0.86 (0.47; 1.58)  14 (3.5) |
|  | Other non-manual/ Skilled manual | 1891 (93.2) | 1.00 (0.80; 1.27)  138 (6.8) | 903 (90.8) | 1.02 (0.77; 1.35)  91 (9.2) | 989 (95.5) | 1.14 (0.76; 1.72)  47 (4.5) |
|  | Professional managers (Reference) | 2522 (93.2) | 183 (6.8) | 1359 (91.0) | 134 (9.0) | 1163 (96.0) | 48 (4.0) |
| Medical card status  (n = 6031  M = 3080  F = 2950) | Yes, full card | 1921 (91.9) | 1.17 (0.96; 1.43)  169 (8.1) | 889 (88.3) | 1.27 (0.99; 1.62)  118 (11.7) | 1032 (95.3) | 1.13 (0.78; 1.63)  51 (4.7) |
|  | Yes, doctor only card | 132 (93.6) | 0.94 (0.47; 1.84)  9 (6.4) | 60 (90.9) | 0.92 (0.39; 2.17)  6 (9.1) | 72 (96.0) | 1.11 (0.37; 3.34)  3 (4.0) |
|  | Not covered (Reference) | 3535 (93.0) | 265 (7.0) | 1817 (90.5) | 190 (9.5) | 1717 (95.6) | 129 (4.4) |

** p < 0.05, ** p <0.01, *** p <0.001*

Table S3 Crude (unadjusted) associations between socioeconomic status (SES) measures (PCGs’ highest educational level, family income, family occupational class and study participants’ medical card status) at 13 years of age and young persons’ parent-reported oral health (number of permanent teeth with dental fillings) at 17/18 years of age.

|  | **Number of teeth with dental fillings** | | | | |
| --- | --- | --- | --- | --- | --- |
| **Socioeconomic measures/indicators** |  | **None**  **n (%)** | **One**  **OR (95% CI)**  **n (%)** | **Two**  **OR (95% CI)**  **n (%)** | **Three or More**  **OR (95% CI)**  **n (%)** |
| Primary care giver’s highest level of education  (n = 5857) | None or primary | 84 (43.1) | 1.19 (0.75; 1.91)  26 (13.3) | **2.37 (1.63; 3.455)*****  56 (28.7) | 1.31 (0.83; 2.06)  29 (14.9) |
|  | Secondary | 2217 (49.9) | 1.16 (0.96; 1.39)  675 (15.2) | **1.26 (1.05; 1.51)***  783 (17.6) | **1.31 (1.09; 1.57)****  765 (17.2) |
|  | Tertiary (Reference) | 677 (55.4) | 178 (14.6) | 189 (15.5) | 178 (14.6) |
| Family income – quintiles  (n = 5421) | Lowest | 484 (44.2) | 1.13 (0.88; 1.45)  162 (14.8) | **1.75 (1.37; 2.22)*****  228 (20.8) | **1.77 (1.39; 2.26)*****  221 (20.2) |
|  | 2^nd^ | 571 (52.3) | 0.94 (0.74; 1.21)  160 (14.7) | **1.37 (1.07; 1.73)***  210 (19.2) | 1.02 (0.79; 1.32)  151 (13.8) |
|  | 3^rd^ | 536 (48.7) | 0.99 (0.77; 1.27)  157 (14.3) | **1.48 (1.16; 1.88)****  213 (19.3) | **1.41 (1.11; 1.81)****  195 (17.7) |
|  | 4^th^ | 585 (52.3) | 1.00 (0.79; 1.28)  175 (15.7) | 1.10 (0.85; 1.39)  171 (15.3) | 1.24 (0.97; 1.59)  187 (16.7) |
|  | Highest (Reference) | 557 (54.9) | 165 (16.3) | 150 (14.8) | 143 (14.1) |
| Family class (Occupation)  (n = 5335) | Semi-skilled/ Unskilled manual | 349 (48.7) | **0.75 (0.57; 0.97)***  81 (11.3) | **1.36 (1.09; 1.170)****  144 (20.1) | **1.46 (1.16; 1.82)****  142 (19.8) |
|  | Other non-manual/ Skilled manual | 1010 (50.8) | 0.90 (0.76; 1.07)  285 (14.3) | 1.13 (0.96; 1.33)  346 (17.4) | **1.22 (1.03; 1.44)***  346 (17.4) |
|  | Professional managers (Reference) | 1388 (52.7) | 433 (16.5) | 422 (16.0) | 389 (14.6) |
| Medical card status  (n = 5858) | Yes, full card | 915 (45.3) | 1.14 (0.97; 1.34)  295 (14.6) | **1.52 (1.31; 1.76)*****  415 (20.5) | **1.53 (1.32; 1.78)*****  396 (19.6) |
|  | Yes, doctor only card | 71 (55.0) | 1.04 (0.63; 1.71)  21 (16.3) | 0.98 (0.59; 1.60)  21 (16.3) | 0.82 (0.47; 1.41)  16 (12.4) |
|  | Not covered (Reference) | 1991 (53.7) | 563 (15.2) | 593 (16.0) | 561 (15.1) |

** p < 0.05, ** p <0.01, *** p <0.001*

Table S4 Crude (unadjusted) associations between socioeconomic status (SES) measures (PCGs’ highest educational level, family income, family occupational class and study participants’ medical card status) at 13 years of age and young persons’ parent-reported oral health (number of permanent teeth with dental fillings) at 17/18 years of age for young males and females.

|  |  | **Males (M)**  **Number of teeth with dental fillings** | | | | **Females (F)**  **Number of teeth with dental fillings** | | | |
| --- | --- | --- | --- | --- | --- | --- | --- | --- | --- |
| **Socioeconomic measures** |  | **None**  **n (%)** | **One**  **OR (95% CI)**  **n (%)** | **Two**  **OR (95% CI)**  **n (%)** | **Three or More**  **OR (95% CI)**  **n (%)** | **None**  **n (%)** | **One**  **OR (95% CI)**  **n (%)** | **Two**  **OR (95% CI)**  **n (%)** | **Three or More**  **OR (95% CI)**  **n (%)** |
| Primary care giver’s highest level of education  (M = 2977  F = 2882) | None or primary | 45 (52.9) | 1.13 (0.58; 2.20)  13 (15.3) | 1.46 (0.82; 2.59)  19 (22.4) | 0.70 (0.32; 1.55)  8 (9.4) | 39 (35.1) | 1.26 (0.65; 2.42)  14 (12.6) | **3.56 (2.14; 5.95)*****  **37 (33.3)** | **1.91 (1.07; 3.41)***  **21 (18.9)** |
|  | Secondary | 1179 (52.7) | 1.12 (0.86; 1.45)  325 (14.5) | 1.05 (0.82; 1.34)  363 (16.2) | 1.28 (0.99; 1.66)  369 (16.5) | 1038 (47.1) | 1.19 (0.92; 1.56)  350 (15.9) | **1.54 (1.18; 2.02)****  **420 (19.1)** | **1.34 (1.03; 1.74)***  **396 (18.0)** |
|  | Tertiary  (Reference) | 367 (55.9) | 91 (13.9) | 108 (16.5) | 90 (13.7) | 310 (54.7) | 87 (15.3) | 82 (14.5) | 88 (15.5) |
| Family income – quintiles  (M = 2758  F = 2664) | Lowest | 260 (48.2) | 0.99 (0.69; 1.41)  72 (13.4) | **1.72 (1.24; 2.40)*****  113 (21.0) | **1.58 (1.11; 2.24)***  94 (17.4) | 225 (40.5) | 1.27 (0.89; 1.80)  90 (16.2) | **1.76 (1.24; 2.48)****  114 (20.5) | **1.94 (1.38; 2.72)*****  127 (22.8) |
|  | 2^nd^ | 306 (55.7) | 0.97 (0.69; 1.36)  83 (15.1) | 1.11 (0.79; 1.57)  86 (15.7) | 1.05 (0.73; 1.51)  74 (13.5) | 265 (48.8) | 0.92 (0.64; 1.31)  77 (14.2) | **1.62 (1.15; 2.27)****  124 (22.8) | 0.99 (0.69; 1.43)  77 (14.2) |
|  | 3^rd^ | 277 (54.0) | 0.817 (0.57; 1.17)  64 (12.5) | 1.27 (0.90; 1.80)  89 (17.3) | 1.31 (0.92; 1.88)  83 (16.2) | 259 (44.0) | 1.12 (0.82; 1.62)  93 (15.8) | **1.66 (1.19; 2.33)****  124 (21.1) | **1.48 (1.05; 2.08)***  112 (19.0) |
|  | 4^th^ | 322 (52.0) | 1.05 (0.75; 1.46)  95 (15.3) | 1.20 (0.86; 1.69)  99 (16.0) | 1.39 (0.98; 1.959  103 (16.6) | 262 (52.5) | 0.96 (0.67; 1.37)  79 (15.8) | 0.96 (0.66; 1.38)  73 (14.6) | 1.11 (0.77; 1.58)  85 (17.0) |
|  | Highest  (Reference) | 305 (56.7) | 86 (16.0) | 77 (14.3) | 70 (13.0) | 252 (52.7) | 79 (16.5) | 73 (15.3) | 74 (15.5) |
| Family class (Occupation)  (M = 2743  F = 2594) | Semi-skilled/ Unskilled manual | 184 (56.1) | **0.58 (0.38; 0.87)****  31 (9.5) | 0.95 (0.68; 1.135)  50 (15.2) | 1.21 (0.87; 1.67)  63 (19.2) | 165 (42.4) | 0.89 (0.63; 1.28)  50 (12.9) | **1.75 (1.29; 2.36)*****  94 (24.2) | **1.73 (1.26; 2.37)*****  80 (20.6) |
|  | Other non-manual/ Skilled manual | 524 (54.3) | 0.87 (0.68; 1.11)  134 (13.9) | 1.02 (0.80; 1.28)  153 (15.9) | 1.04 (0.83; 1.32)  154 (16.0) | 486 (47.6) | 0.93 (0.73; 1.18)  151 (14.8) | 1.22 (0.97; 1.54)  193 (18.9) | **1.42 (1.12; 1.80)****  192 (18.8) |
|  | Professional managers  (Reference) | 778 (53.7) | 229 (15.8) | 224 (15.4) | 219 (15.1) | 610 (51.6) | 205 (17.3) | 198 (16.7) | 170 (14.4) |
| Medical card status  (M = 2975  F = 2882) | Yes, full card | 468 (48.5) | 0.93 (0.73; 1.18)  120 (12.4) | **1.65 (1.33; 2.03)*****  201 (20.9) | **1.42 (1.13; 1.76)****  175 (18.2) | 447 (42.3) | **1.35 (1.08; 1.68)****  176 (16.7) | **1.40 (1.14; 1.72)****  214 ( | **1.635 (1.32; 2.02)***** |
|  | Yes, doctor only card | 42 (65.6) | 0.92 (0.46; 1.82)  11 (17.2) | 0.58 (0.25; 1.36)  6 (9.4) | 0.48 (0.19; 1.19)  5 (7.8) | 30 (46.2) | 1.20 (0.59; 2.48)  10 (15.4) | 1.42 (0.75; 2.71)  14 (21.5) | 1.24 (0.61; 2.51)  11 (16.9) |
|  | Not covered  (Reference) | 1081 (55.5) | 298 (15.3)) | 282 (14.5) | 286 (14.7) | 910 (51.7) | 265 (15.1) | 311 (17.7) | 274 (15.6) |

** p < 0.05, ** p <0.01, *** p <0.001*

Table S5 Crude (unadjusted) associations between behavioural factors (daily tooth brushing frequency, dentist visit behaviour, sugary foods intake and sugary drinks intake) at 13 years of age and young persons’ parent-reported oral health (number of teeth with dental fillings) at 17/18 years of age.

|  |  | **Number of teeth with dental fillings** | | | |
| --- | --- | --- | --- | --- | --- |
| **Behavioural factors** |  | **None**  **n (%)** | **One**  **OR (95% CI)**  **n (%)** | **Two**  **OR (95% CI)**  **n (%)** | **Three or More**  **OR (95% CI)**  **n (%)** |
| Toothbrushing frequency  (n = 5782) | Less than once a day/Rarely/Not at all | 154 (49) | 0.82 (0.57; 1.19)  37 (11.8) | 1.00 (0.72; 1.38)  52 (16.6) | **1.60 (1.19; 2.15)****  71 (22.6) |
|  | Once a day | 587 (48) | 1.02 (0.84; 1.23)  177 (14.5) | 1.12 (0.94; 1.33)  221 (18.1) | **1.39 (1.17; 1.66)*****  237 (19.4) |
|  | Twice or more than twice a day  (Reference) | 2208 (52) | 654 (15.4) | 745 (17.5) | 639 (15.0) |
| Dentist visit behaviour  (n = 5855) | Problem visit/Never/Almost never | 455 (56.9) | 0.99 (0.80; 1.22)  139 (17.4) | **0.62 (0.49; 0.77)*****  107 (13.4) | **0.59 (0.47; 0.75)*****  98 (12.3) |
|  | Occasional visit | 598 (53.7) | **0.78 (0.64; 0.96)***  145 (13.0) | 0.83 (0.69; 1.00)  190 (17.1) | 0.83 (0.69; 1.00)  181 (16.2) |
|  | At least a year  (Reference) | 1923 (48.8) | 594 (15.1) | 731 (18.5) | 694 (17.6) |
| Sugary drinks intake  (Soft drinks/minerals/cordial/squash (not diet)  (n = 5771) | More than once a day | 523 (51.1) | 0.82 (0.66; 1.02)  133 (13.0) | 1.16 (0.95; 1.40)  186 (18.2) | 1.19 (0.98; 1.45)  182 (17.8) |
|  | Once a day | 799 (48.2) | 0.96 (0.80; 1.14)  236 (14.2) | **1.36 (1.16; 1.60)*****  333 (20.1) | **1.25 (1.05; 1.47)***  291 (17.5) |
|  | Not at all  (Reference) | 1619 (52.4) | 499 (16.2) | 497 (16.1) | 473 (15.3) |
| Sugary foods intake  (Biscuits, doughnuts, cake, pie or chocolate)  (n = 5776) | More than once a day | 543 (47.1) | 1.07 (0.86; 1.33)  167 (14.5) | **1.32 (1.07; 1.62)****  221 (19.2) | **1.40 (1.14; 1.72)****  223 (19.3) |
|  | Once a day | 1485 (51.4) | 1.02 (0.86; 1.22)  438 (15.2) | 1.12 (0.94; 1.32)  513 (17.8) | 1.04 (0.87; 1.23)  453 (15.7) |
|  | Not at all  (Reference) | 917 (52.9) | 263 (15.2) | 283 (16.3) | 270 (15.6) |

** p < 0.05, ** p <0.01, *** p <0.001*

Table S6 Crude (unadjusted) associations between behavioural factors (daily tooth brushing frequency, dentist visit behaviour, sugary foods intake and sugary drinks intake) at 13 years of age and young persons’ parent-reported oral health (number of teeth with dental fillings) at 17/18 years of age and by gender.

|  |  | **Males (M)**  **Number of teeth with dental fillings** | | | | **Females (F)**  **Number of teeth with dental fillings** | | | |
| --- | --- | --- | --- | --- | --- | --- | --- | --- | --- |
| **Behavioural factors** |  | **None**  **n (%)** | **One**  **OR (95% CI)**  **n (%)** | **Two**  **OR (95% CI)**  **n (%)** | **Three or More**  **OR (95% CI)**  **n (%)** | **None**  **n (%)** | **One**  **OR (95% CI)**  **n (%)** | **Two**  **OR (95% CI)**  **n (%)** | **Three or More**  **OR (95% CI)**  **n (%)** |
| Toothbrushing frequency  (M = 2931  F = 2851) | Less than once a day/Rarely/Not at all | 114 (52.5) | 1.02 (0.68; 1.54)  33 (15.2) | 0.98 (0.65; 1.48)  32 (14.7) | 1.31 (0.89; 1.93)  38 (17.5) | 39 (40.6) | 0.39 (0.15; 1.01)  5 (5.2) | 1.29 (0.74; 2.25)  19 (19.8) | **2.61 (1.62; 4.21)*****  33 (34.4) |
|  | Once a day | 387 (51.0) | 0.84 (0.65; 1.09)  91 (12.0) | **1.28 (1.02; 1.61)***  144 (19.0) | **1.37 (1.08; 1.74)****  137 (18.1) | 200 (43.1) | **1.38 (1.04; 1.82)***  86 (18.5) | 1.01 (0.76; 1.35)  78 (16.8) | **1.57 (1.20; 2.05)*****  100 (21.6) |
|  | Twice or more than twice a day  (Reference) | 1072 (54.8) | 298 (15.2) | 310 (15.9) | 275 (14.1) | 1136 (49.6) | 356 (15.5) | 435 (19.0) | 364 (15.9) |
| Dentist visit behaviour  (M = 2974  F = 2880) | Problem visit/Never/Almost never | 243 (59.6) | 0.89 (0.65; 1.21)  61 (15.0) | **0.69 (0.51; 0.95)***  57 (14.0) | **0.61 (0.43; 0.85)****  47 (11.5) | 211 (54.1) | 1.09 (0.81; 1.46)  78 (20.0) | **0.55 (0.39; 0.76)*****  50 (12.8) | **0.59 (0.42; 0.82)****  51 (13.1) |
|  | Occasional visit | 310 (55.9) | 0.84 (0.63; 1.11)  73 (13.2) | 0.83 (0.64; 1.09)  86 (15.5) | 0.86 (0.66; 1.13)  86 (15.5) | 288 (51.6) | **0.74 (0.55; 0.98)***  72 (12.9) | 0.83 (0.65; 1.07)  104 (18.6) | 0.80 (0.62; 1.04)  94 (16.8) |
|  | At least a year  (Reference) | 1037 (51.6) | 294 (14.6) | 347 (17.3) | 333 (16.6) | 886 (45.9) | 301 (15.6) | 384 (19.9) | 361 (18.7) |

** p < 0.05, ** p <0.01, *** p <0.001*

Table S6 Continued….

|  |  | **None**  **n (%)** | **One**  **OR (95% CI)**  **n (%)** | **Two**  **OR (95% CI)**  **n (%)** | **Three or More**  **OR (95% CI)**  **n (%)** | **None**  **n (%)** | **One**  **OR (95% CI)**  **n (%)** | **Two**  **OR (95% CI)**  **n (%)** | **Three or More**  **OR (95% CI)**  **n (%)** |
| --- | --- | --- | --- | --- | --- | --- | --- | --- | --- |
| Sugary drinks intake  (M = 2926  F = 2844) | More than once a day | 309 (55.2) | **0.65 (0.48; 0.89)****  63 (11.3) | 1.20 (0.92; 1.58)  102 (18.2) | 1.09 (0.82; 1.46)  86 (15.4) | 214 (46.2) | 1.07 (0.79; 1.45)  70 (15.1) | 1.16 (0.87; 1.54)  83 (17.9) | **1.38 (1.05; 1.82)***  96 (20.7) |
|  | Once a day | 497 (51.9) | **0.76 (0.59; 0.98)***  119 (12.4) | 1.26 (1.00; 1.59)  173 (18.1) | **1.33 (1.05; 1.68)***  168 (17.6) | 302 (43.0) | 1.27 (0.98; 1.64)  117 (16.7) | **1.58 (1.25; 2.00)*****  161 (22.9) | 1.24 (0.97; 1.59)  122 (17.4) |
|  | Not at all  (Reference) | 765 (54.3) | 239 (17.0) | 210 (14.9) | 195 (13.8) | 854 (50.9) | 260 (15.5) | 287 (17.1) | 278 (16.6) |
| Sugary foods intake  (M = 2927  F = 2851) | More than once a day | 288 (48.2) | 1.18 (0.86; 1.63)  80 (13.4) | **1.58 (1.19; 2.09)*****  132 (22.1) | 1.13 (0.84; 1.53)  97 (16.2) | 255 (45.8) | 0.99 (0.73; 1.35)  86 (15.4) | 1.07 (0.79; 1.45)  90 (16.2) | **1.69 (1.27; 2.25)*****  126 (22.6) |
|  | Once a day | 818 (55.1) | 1.19 (0.93; 1.55)  232 (15.6) | 0.93 (0.73; 1.19)  220 (14.8) | 0.89 (0.70; 1.14)  215 (14.5) | 667 (47.5) | 0.91 (0.72; 1.16)  207 (14.7) | **1.34 (1.06; 1.68)***  293 (20.9) | 1.21 (0.95; 1.55)  238 (16.9) |
|  | Not at all  (Reference) | 464 (54.9) | 110 (13.0) | 134 (15.9) | 137 (16.2) | 453 (51.0) | 174 (17.3) | 149 (16.8) | 133 (15.0) |

** p < 0.05, ** p <0.01, *** p <0.001*

Table S7 Crude (unadjusted) associations between behavioural factors (daily tooth brushing frequency, dentist visit behaviour, sugary foods intake and sugary drinks intake) at 13 years of age and young persons’ self-reported oral health (self-rated oral health, SROH) at 17/18 years of age and by gender.

|  |  | **Total**  **Self-reported oral health** | | **Males**  **Self-reported oral health** | | **Females**  **Self-reported oral health** | |
| --- | --- | --- | --- | --- | --- | --- | --- |
|  |  | **Optimal oral health**  **n (%)** | **Suboptimal oral health**  **OR (95% CI)**  **n (%)** | **Optimal oral health**  **n (%)** | **Suboptimal oral health**  **OR (95% CI)**  **n (%)** | **Optimal oral health**  **n (%)** | **Suboptimal oral health**  **OR (95% CI)**  **n (%)** |
| Toothbrushing frequency  (n = 5960  M = 3036  F = 2923) | Less than once a day/Rarely/Not at all | 253 (77.6) | **5.15 (3.85; 6.90)*****  73 (22.4) | 163 (73.1) | **4.95 (3.51; 6.07)*****  60 (26.9) | 90 (87.4) | **3.65 (1.99; 6.72)*****  13 (12.6) |
|  | Once a day | 1132 (89.9) | **2.00 (1.60; 2.51)*****  127 (10.1) | 673 (86.5) | **2.11 (1.61; 2.75)*****  105 (13.5) | 459 (95.4) | 1.18 (0.73; 1.89)  22 (4.6) |
|  | Twice or more than twice a day  (Reference) | 4143 (94.7) | 232 (5.3) | 1895 (93.1) | 140 (6.9) | 2247 (96.1) | 92 (3.9) |
| Dentist visit behaviour  (n = 6031  M = 3082  F = 2949) | Problem visit/Never/Almost never | 740 (88.6) | **1.70 (1.33; 2.17)*****  95 (11.4) | 354 (81.8) | **2.16 (1.62; 2.87)*****  79 (18.2) | 386 (96.0) | 0.87 (0.50; 1.49)  16 (4.0) |
|  | Occasional visit | 1079 (94.2) | 0.81 (0.62; 1.07)  66 (5.8) | 527 (92.6) | 0.77 (0.54; 1.09)  42 (7.4) | 552 (95.8) | 0.91 (0.58; 1.45)  24 (4.2) |
|  | At least a year  (Reference) | 3767 (93.0) | 284 (7.0) | 1886 (90.7) | 194 (9.3) | 1881 (95.4) | 90 (4.6) |

** p < 0.05, ** p <0.01, *** p <0.001*

Table S7 Continued….

|  |  | **Optimal oral health**  **n (%)** | **Suboptimal oral health**  **OR (95% CI)**  **n (%)** | **Optimal oral health**  **n (%)** | **Suboptimal oral health**  **OR (95% CI)**  **n (%)** | **Optimal oral health**  **n (%)** | **Suboptimal oral health**  **OR (95% CI)**  **n (%)** |
| --- | --- | --- | --- | --- | --- | --- | --- |
| Sugary drinks intake  (Soft drinks/minerals/cordial/squash (not diet)  (n = 5950  M = 3032  F = 2919) | More than once a day | 956 (89.3) | **1.99 (1.56; 2.55)*****  114 (10.7) | 508 (86.5) | **1.63 (1.21; 2.20)****  79 (13.5) | 448 (92.6) | **2.49 (1.61; 3.85)*****  36 (7.4) |
|  | Once a day | 1569 (92.0) | **1.46 (1.16; 1.84)****  137 (8.0) | 892 (90.0) | 1.17 (0.88; 1.54)  99 (10.0) | 677 (94.6) | **1.79 (1.17; 2.74)****  39 (5.4) |
|  | Not at all  (Reference) | 2995 (94.4) | 179 (5.6) | 1328 (91.3) | 126 (8.7) | 1666 (96.9) | 53 (3.1) |
| Sugary foods intake  (Biscuits, doughnuts, cake, pie or chocolate)  (n = 5954  M = 3033  F = 2923) | More than once a day | 1049 (88.9) | **1.71 (1.31; 2.21)*****  131 (11.1) | 522 (84.9) | **1.59 (1.17; 2.18)****  93 (15.1) | 526 (93.3) | **1.89 (1.18; 3.05)****  38 (6.7) |
|  | Once a day | 2791 (94.1) | 0.86 (0.68; 1.09)  175 (5.9) | 1401 (92.1) | 0.77 (0.58; 1.02)  120 (7.9) | 1390 (96.1) | 1.05 (0.68; 1.62)  56 (3.9) |
|  | Not at all  (Reference) | 1685 (93.2) | 123 (6.8) | 807 (90.0) | 90 (10.0) | 879 (96.3) | 34 (3.7) |

** p < 0.05, ** p <0.01, *** p <0.001*

Table S8 Crude (Unadjusted) associations between material factors (financial difficulties, DEIS school status, house ownership and private medical insurance cover) at 13 years of age and young persons’ parent-reported oral health (number of teeth with dental fillings) at 17/18 years of age.

|  |  | **Number of teeth with dental fillings** | | | |
| --- | --- | --- | --- | --- | --- |
|  |  | **None**  **n (%)** | **One**  **OR (95% CI)**  **n (%)** | **Two**  **OR (95% CI)**  **n (%)** | **Three or More**  **OR (95% CI)**  **n (%)** |
| Financial difficulties (Difficulty in meeting ends)  (n = 5856) | With difficulty | 1753 (49.9) | 0.90 (0.77; 1.05)  496 (14.1) | 1.04 (0.90; 1.20)  616 (17.5) | **1.41 (1.21; 1.65)*****  651 (18.5) |
|  | No difficulty  (Reference) | 1223 (52.3) | 383 (16.4) | 413 (17.6) | 321 (13.7) |
| DEIS school  (n = 5435) | Yes | 488 (49.6) | **0.79 (0.64; 0.98)***  119 (12.1) | **1.25 (1.04; 1.50)***  203 (20.6) | 1.12 (0.92; 1.35)  174 (17.7) |
|  | No  (Reference) | 2274 (51.1) | 696 (15.6) | 755 (17.0) | 726 (16.3) |
| Homeownership  (n = 5854) | Renters/Rent free/Others | 522 (46.8) | 0.87 (0.71; 1.07)  138 (12.4) | **1.46 (1.23; 1.73)*****  243 (21.8) | **1.31 (1.09; 1.57)****  212 (19.0) |
|  | Owner  (Reference) | 2453 (51.8) | 742 (15.7) | 783 (16.5) | 761 (16.1) |
| Private medical insurance  (n = 5859) | No | 1432 (48.9) | 0.94 (0.81; 1.09)  409 (14.0) | **1.28 (1.11; 1.47)*****  558 (19.1) | **1.28 (1.11; 1.48)*****  529 (18.1) |
|  | Yes  (Reference) | 1545 (52.7) | 471 (16.1) | 471 (16.1) | 444 (15.1) |

** p < 0.05, ** p <0.01, *** p <0.001*

Table S9 Crude (Unadjusted) associations between material factors (financial difficulties, DEIS school status, house ownership and private medical insurance cover) at 13 years of age and young persons’ parent-reported oral health (number of teeth with dental fillings) at 17/18 years of age and by gender.

|  |  | **Males (M)**  **Number of teeth with dental fillings** | | | | **Females (F)**  **Number of teeth with dental fillings** | | | |
| --- | --- | --- | --- | --- | --- | --- | --- | --- | --- |
|  |  | **None**  **n (%)** | **One**  **OR (95% CI)**  **n (%)** | **Two**  **OR (95% CI)**  **n (%)** | **Three or More**  **OR (95% CI)**  **n (%)** | **None**  **n (%)** | **One**  **OR (95% CI)**  **n (%)** | **Two**  **OR (95% CI)**  **n (%)** | **Three or More**  **OR (95% CI)**  **n (%)** |
| Financial difficulties (Difficulty in meeting ends)  (M = 2974  F = 2883) | With difficulty | 924 (53.5) | 0.87 (0.70; 1.08)  235 (13.6) | 0.89 (0.73; 1.09)  271 (15.7) | **1.27 (1.03; 1.58)***  298 (17.2) | 829 (46.4) | 0.93 (0.75; 1.15)  261 (14.6) | 1.19 (0.98; 1.47)  345 (19.3) | **1.56 (1.25; 1.93)*****  353 (19.7) |
|  | No difficulty  (Reference) | 665 (53.4) | 194 (15.6) | 219 (17.6) | 168 (13.5) | 558 (51.0) | 190 (17.4) | 194 (17.7) | 153 (14.0) |
| DEIS school  M = 2782  F = 2656) | Yes | 258 (51.1) | **0.67 (0.48; 0.93)***  50 (9.9) | **1.35 (1.04; 1.75)***  102 (20.2) | 1.28 (0.98; 1.66)  95 (18.8) | 230 (47.9) | 0.91 (0.68; 1.23)  69 (14.4) | 1.16 (0.89; 1.50)  101 (21.0) | 0.97 (0.73; 1.28)  80 (16.7) |
|  | No  (Reference) | 1220 (53.6) | 350 (15.4) | 357 (15.7) | 350 (15.4) | 1054 (48.4) | 347 (15.9) | 398 (18.3) | 377 (17.3) |
| Homeownership  (M = 2974  F = 2880) | Renters/Rent free/Others | 251 (50.5) | **0.61 (0.43; 0.85)****  44 (8.9) | **1.58 (1.23; 2.03)*****  112 (22.5) | 1.27 (0.97; 1.65)  90 (18.1) | 272 (43.9) | 1.08 (0.83; 1.41)  94 (15.2) | **1.33 (1.05; 1.68)***  231 (21.1) | **1.31 (1.03; 1.67)***  123 (19.8) |
|  | Owner  (Reference) | 1337 (54.0) | 385 (15.5) | 378 (15.3) | 377 (15.2) | 1115 (49.3) | 357 (15.8) | 405 (17.9) | 383 (16.9) |
| Private medical insurance  (M = 2977  F = 2879) | No | 732 (52.5) | 0.82 (0.66; 1.02)  177 (12.7) | **1.27 (1.03; 1.55)***  254 (18.2) | 1.16 (0.94; 1.42)  232 (16.6) | 700 (45.7) | 1.04 (0.84; 1.28)  232 (15.2) | **1.26 (1.03; 1.54)***  303 (19.8) | **1.37 (1.13; 1.70)****  296 (19.3) |
|  | Yes (Reference) | 859 (54.3) | 252 (15.9) | 236 (14.9) | 235 (14.9) | 685 (50.8) | 219 (16.2) | 235 (17.4) | 209 (15.5) |

** p < 0.05, ** p <0.01, *** p <0.001*

Table S10 Crude (Unadjusted) associations between material factors (financial difficulties, DEIS school status, house ownership and private medical insurance cover) at 13 years of age and young persons’ self-reported oral health (self-reported oral health) at 17/18 years of age and by gender.

|  |  | **Total**  **Self-reported oral health** | | **Males**  **Self-reported oral health** | | **Females**  **Self-reported oral health** | |
| --- | --- | --- | --- | --- | --- | --- | --- |
|  |  | **Optimal oral health**  **n (%)** | **Suboptimal oral health**  **OR (95% CI)**  **n (%)** | **Optimal oral health**  **n (%)** | **Suboptimal oral health**  **OR (95% CI)**  **n (%)** | **Optimal oral health**  **n (%)** | **Suboptimal oral health**  **OR (95% CI)**  **n (%)** |
| Financial difficulties (Difficulty in meeting ends)  (n = 6030  M = 3080  F = 2951) | With difficulty | 3337 (92.1) | **1.25 (1.02; 1.53)***  288 (7.9) | 1589 (88.7) | **1.34 (1.05; 1.70)***  203 (11.3) | 1748 (95.3) | 1.19 (0.82; 1.74)  86 (4.7) |
|  | No difficulty  (Reference) | 2249 (93.5) | 156 (6.5) | 1176 (91.3) | 112 (8.7) | 1073 (96.1) | 44 (3.9) |
| DEIS school  (n = 5591  M = 2870  F = 2720) | Yes | 916 (90.3) | **1.48 (1.17; 1.88)****  98 (9.7) | 463 (88.4) | 1.26 (0.94; 1.71)  61 (11.6) | 453 (92.4) | **2.01 (1.35; 3.00)*****  37 (7.6) |
|  | No  (Reference) | 4268 (93.2) | 309 (6.8) | 2124 (90.5) | 222 (9.5) | 2144 (96.1) | 86 (3.9) |
| Homeownership  (n = 6026  M = 3078  F = 2949) | Renters/Rent free/Others | 1042 (90.3) | **1.48 (1.18; 1.85)*****  112 (9.7) | 439 (84.7) | **1.79 (1.36; 2.35)*****  79 (15.3) | 604 (94.8) | 1.26 (0.84; 1.88)  33 (5.2) |
|  | Owners  (Reference) | 4540 (93.2) | 332 (6.8) | 2325 (90.8) | 235 (9.2) | 2215 (95.8) | 97 (4.2) |
| Private medical insurance  (n = 6032  M = 3081  F = 2949) | No | 2745 (90.7) | **1.76 (1.45; 2.17)*****  281 (9.3) | 1250 (86.1) | **2.16 (1.69; 2.75)*****  201 (13.9) | 1495 (95.0) | 1.39 (0.97; 1.99)  79 (5.0) |
|  | Yes  (Reference) | 2842 (94.5) | 164 (5.5) | 1517 (93.1) | 113 (6.9) | 1325 (96.4) | 50 (3.6) |

** p < 0.05, ** p <0.01, *** p <0.001*

Table S11 Crude (Unadjusted) associations between psychosocial factors (PCG depression status, family structure, job stress, and PCG mean parental stress scale score) at 13 years of age and young persons’ parent-reported oral health (number of teeth with dental fillings) at 17/18 years of age.

|  |  | **Number of dental fillings** | | | |
| --- | --- | --- | --- | --- | --- |
|  |  | **None**  **n (%)** | **One**  **OR (95% CI)**  **n (%)** | **Two**  **OR (95% CI)**  **n (%)** | **Three or More**  **OR (95% CI)**  **n (%)** |
| PCG depression status  (n = 5802) | Depressed | 329 (50.5) | 0.80 (0.62; 1.04)  81 (12.4) | 0.99 (0.79; 1.240  113 (17.4) | 1.20 (0.97; 1.49)  128 (19.7) |
|  | Not depressed  (Reference) | 2612 (50.7) | 793 (15.4) | 905 (17.6) | 841 (16.3) |
| Family structure  (n = 5859) | Single parent family | 480 (44.6) | 1.21 (0.99; 1.47)  166 (15.4) | **1.51 (1.27; 1.80)*****  231 (21.5) | **1.34 (1.11; 1.61)****  199 (18.5) |
|  | Couple family  (Reference) | 2498 (52.2) | 714 (14.9) | 797 (16.7) | 774 (16.2) |
| Job stress  (n = 5522) | Stressed | 1951 (52.3) | **0.69 (0.55; 0.87)****  525 (14.1) | 0.99 (0.79; 1.24)  661 (17.7) | 0.93 (0.74; 1.17)  590 (15.8) |
|  | Not stressed  (Reference) | 862 (48.0) | 302 (16.8) | 314 (17.5) | 317 (17.7) |
| PCG Parental Stressor scale score  (n = 5799) |  | 2942 (50.73) | 10.01 (0.99; 1.03)  874 (15.07) | **1.03 (1.01, 1.05)*****  1018 (17.55) | **1.03 (1.01, 1.05)*****  965 (16.64) |

** p < 0.05, ** p <0.01, *** p <0.001*

Table S12 Crude (Unadjusted) associations between psychosocial factors (PCG depression status, family structure, job stress and PCG mean parental stress scale score) at 13 years of age and young persons’ parent-reported oral health (number of teeth with dental fillings) at 17/18 years of age and by gender.

|  |  | **Males (M)**  **Number of dental fillings** | | | | **Females (F)**  **Number of dental fillings** | | | |
| --- | --- | --- | --- | --- | --- | --- | --- | --- | --- |
|  |  | **None**  **n (%)** | **One**  **OR (95% CI)**  **n (%)** | **Two**  **OR (95% CI)**  **n (%)** | **Three or More**  **OR (95% CI)**  **n (%)** | **None**  **n (%)** | **One**  **OR (95% CI)**  **n (%)** | **Two**  **OR (95% CI)**  **n (%)** | **Three or More**  **OR (95% CI)**  **n (%)** |
| PCG depression status  (M = 2946  F = 2856) | Depressed | 188 (55.5) | **0.67 (0.46; 0.98)***  36 (10.6) | 0.96 (0.69; 1.31)  56 (16.5) | 1.07 (0.78; 1.46)  59 (17.4) | 141 (45.3) | 0.97 (0.68; 1.38)  45 (14.5) | 1.05 (0.76; 1.45)  57 (18.3) | 1.37 (1.00; 1.86)  68 (21.9) |
|  | Not depressed  (Reference) | 1382 (53.0) | 389 (14.9) | 429 (16.5) | 407 (15.6) | 1230 (48.3) | 404 (15.9) | 476 (18.7) | 435 (17.1) |
| Family structure  (M = 2975  F = 2883) | Single parent family | 235 (45.8) | 1.02 (0.76; 1.38)  64 (12.5) | **1.80 (1.40; 2.31)*****  116 (22.6) | **1.53 (1.18; 1.99)****  98 (19.1) | 245 (43.6) | **1.35 (1.04; 1.75)***  101 (18.0) | 1.27 (0.99; 1.62)  115 (20.5) | 1.16 (0.90; 1.51)  101 (18.0) |
|  | Couple family  (Reference) | 1356 (55.1) | 364 (14.8) | 373 (15.2) | 369 (15.0) | 1142 (49.2) | 350 (15.1) | 424 (18.3) | 405 (17.4) |
| Job stress  (M = 2818  F = 2704) | Stressed | 1025 (55.1) | 0.84 (0.67; 1.06)  259 (13.9) | 0.86 (0.69; 1.07)  302 (16.2) | **0.72 (0.58; 0.90)****  274 (14.7) | 926 (49.6) | **0.69 (0.55; 0.87)****  266 (14.2) | 0.99 (0.79; 1.24)  359 (19.2) | 0.93 (0.74; 1.17)  317 (17.0) |
|  | Not stressed  (Reference) | 477 (49.8) | 143 (14.9) | 163 (17.0) | 175 (18.3) | 385 (46.1) | 159 (19.0) | 151 (18.1) | 141 (16.9) |
| PCG Parental Stressor scale score  (M = 2943; F = 2856) |  | 1571 (53.4) | 1.01 (0.98; 1.04)  425 (14.4) | 1.01 (0.98; 1.04)  485 (16.5) | **1.04 (1.02; 1.07) *****  462 (15.7) | 1371 (48.0) | 1.01 (0.98; 1.03)  449 (15.7) | **1.05 (1.02; 1.07) *****  533 (18.7) | 1.02 (0.99; 1.04)  503 (17.6) |

** p < 0.05, ** p <0.01, *** p <0.001*

Table S13 Crude (Unadjusted) associations between psychosocial factors (PCG depression status, family structure, job stress and PCG mean parental stress scale score) at 13 years of age and young persons’ parent-reported oral health (number of teeth with dental fillings) at 17/18 years of age and by gender.

|  |  | **Total**  **Self-reported oral health** | | **Males**  **Self-reported oral health** | | **Females**  **Self-reported oral health** | |
| --- | --- | --- | --- | --- | --- | --- | --- |
|  |  | **Optimal oral health**  **n (%)** | **Suboptimal oral health**  **OR (95% CI)**  **n (%)** | **Optimal oral health**  **n (%)** | **Suboptimal oral health**  **OR (95% CI)**  **n (%)** | **Optimal oral health**  **n (%)** | **Suboptimal oral health**  **OR (95% CI)**  **n (%)** |
| PCG depression status  (n = 5972  M = 3047  F = 2925) | Depressed | 617 (92.1) | 1.11 (0.78; 1.58)  53 (7.9) | 314 (89.0) | 1.11 (0.77; 1.58)  39 (11.0) | 304 (95.6) | 0.97 (0.55; 1.73)  14 (4.4) |
|  | Not depressed  (Reference) | 4912 (92.6) | 390 (7.4) | 2419 (89.8) | 275 (10.2) | 2492 (95.6) | 115 (4.4) |
| Family structure  (n = 6032  M = 3082  F = 2951) | Single parent family | 1014 (91.0) | **1.31 (1.04; 1.66)***  100 (9.0) | 462 (87.0) | **1.39 (1.05; 1.85)***  69 (13.0) | 552 (94.7) | 1.32 (0.87; 1.99)  31 (5.3) |
|  | Couple family  (Reference) | 4574 (93.0) | 344 (7.0) | 2305 (90.4) | 246 (9.6) | 2270 (95.9) | 98 (4.1) |
| Job stress  (n = 5679  M = 2901  F = 2777) | Stressed | 3554 (92.7) | 0.96 (0.78; 1.19)  278 (7.3) | 1725 (90.0) | 0.99 (0.77; 1.28)  192 (10.0) | 1828 (95.5) | 0.98 (0.66; 1.44)  86 (4.5) |
|  | Not stressed  (Reference) | 1708 (92.5) | 139 (7.5) | 885 (89.9) | 99 (10.1) | 823 (95.4) | 40 (4.6) |
| PCG Parental Stressor scale score  (n = 5968  M = 3044  F = 2924) |  | 5527 (92.6) | **1.04 (1.02; 1.06)*****  442 (7.4) | 2731 (89.7) | **1.05 (1.03; 1.08)*****  313 (10.3) | 2796 (95.6) | 1.00 (0.96; 1.05)  128 (4.4) |

** p < 0.05, ** p <0.01, *** p <0.001*

Table S14 Association between SES components and toothbrushing

|  |  | **Total** |  | **Males** |  | **Females** |  |
| --- | --- | --- | --- | --- | --- | --- | --- |
|  | **Toothbrushing frequency** | **Once a day** | **Less than once a day/Rarely/Not at all** | **Once a day** | **Less than once a day/Rarely/Not at all** | **Once a day** | **Less than once a day/Rarely/Not at all** |
|  |  | **OR**  **(95% CI)**  ***(p-value)*** | **OR**  **(95% CI)**  ***(p-value)*** | **OR**  **(95% CI)**  ***(p-value)*** | **OR**  **(95% CI)**  ***(p-value)*** | **OR**  **(95% CI)**  ***(p-value)*** | **OR**  **(95% CI)**  ***(p-value)*** |
| Primary care giver’s highest level of education  (n = 6039) | None or primary | 1.38 (0.98; 1.94)  (p = 0.064) | 0.87 (0.36; 2.11)  (p = 0.770) | 1.57 (1.00; 2.45)  (p = 0.049) | 0.42 (0.10; 1.77)  (p = 0.240) | 1.27 (0.73; 2.22)  (p = 0.392) | 3.06 (0.86; 10.82)  (p = 0.083) |
|  | Secondary | **1.18 (1.00; 1.38)**  **(p = 0.039)** | **1.95 (1.40; 2.71)**  **(p < 0.001)** | 1.13 (0.92; 1.38)  (p = 0.235) | **1.66 (1.14; 2.42)**  **(p = 0.008)** | **1.35 (1.04; 1.75)**  **(p = 0.025)** | **3.72 (1.71; 8.07)**  **(p < 0.001)** |
|  | Tertiary | **Reference** |  | **Reference** |  | **Reference** |  |
| Family income – quintiles  (n = 5586) | Lowest | **1.76 (1.43; 2.17)**  **(p < 0.001)** | **3.00 (1.92; 4.68)**  **(p < 0.001)** | **1.34 (1.02; 1.76)**  **(p = 0.033)** | **3.27 (1.87; 5.71)**  (p < 0.001) | **2.99 (2.09; 4.29)**  **(p < 0.001)** | **2.65 (1.25; 5.61)**  **(p = 0.011)** |
|  | 2^nd^ | **1.45 (1.17; 1.80)**  **(p < 0.001)** | **3.47 (2.24; 5.38)**  **(p < 0.001)** | 1.30 (0.99; 1.71)  (p = 0.060) | **3.72 (2.14; 6.45)**  **(p < 0.001)** | **1.99 (1.37; 2.91)**  **(p < 0.001)** | **3.27 (1.58; 6.76)**  **(p = 0.001)** |
|  | 3^rd^ | **1.48 (1.19; 1.84)**  **(p < 0.001)** | **2.09 (1.31; 3.33)**  **(p = 0.002)** | **1.48 (1.12; 1.95)**  **(p = 0.005)** | **2.51 (1.39; 4.52)**  **(p = 0.002)** | **1.91 (1.31; 2.77)**  **(p < 0.001)** | 1.81 (0.83; 3.93)  (p = 0.136) |
|  | 4^th^ | 1.11 (0.89; 1.38)  (p = 0.369) | **2.13 (1.34; 3.37)**  **(p = 0.001)** | 0.82 (0.62; 1.08)  (p = 0.159) | **2.29 (1.30; 4.03)**  **(p = 0.004)** | **1.86 (1.27; 2.74)**  **(p = 0.002)** | 1.54 (0.67; 3.51)  (p = 0.309) |
|  | Highest | **Reference** |  | **Reference** |  | **Reference** |  |
| Family class (Occupation)  (n = 5481) | Semi-skilled/ Unskilled manual | **1.52 (1.25; 1.84)**  **(p < 0.001)** | **1.73 (1.18; 2.53)**  **(p = 0.005)** | **1.36 (1.04; 1.77)**  **(p = 0.026)** | **2.24 (1.44; 3.48)**  **(p < 0.001)** | **2.14 (1.60; 2.85)**  **(p < 0.001)** | 1.23 (0.55; 2.75)  (p = 0.617) |
|  | Other non-manual/ Skilled manual | **1.16 (1.01; 1.34)**  **(p = 0.039)** | **1.92 (1.47; 2.52)**  **(p < 0.001)** | 1.07 (0.89; 1.29)  (p = 0.458) | **1.79 (1.29; 2.49)**  **(p < 0.001)** | **1.50 (1.18; 1.89)**  **(p < 0.001)** | **2.63 (1.59; 4.36)**  **(p < 0.001)** |
|  | Professional managers | **Reference** |  | **Reference** |  | **Reference** |  |
| Medical card status  (n = 6039) | Yes, full card | **1.29 (1.13; 1.47)**  **(p < 0.001)** | **2.15 (1.72; 2.70)**  **(p < 0.001)** | 1.15 (0.96; 1.37)  (p = 0.133) | **1.97 (1.49; 2.61)**  **(p < 0.001)** | **1.65 (1.35; 2.01)**  **(p < 0.001)** | **2.95 (1.96; 4.44)**  **(p < 0.001)** |
|  | Yes, doctor only card | **1.64 (1.13; 2.38)**  **(p = 0.009)** | 0.62 (0.20; 1.88)  (p = 0.397) | **1.93 (1.16; 3.10)**  **(p = 0.011)** | 0.13 (0.01; 2.59)  (p = 0.184) | 1.48 (0.82; 2.67)  (p = 0.198) | 1.85 (0.54; 6.35)  (p = 0.330) |
|  | Not covered | **Reference** |  | **Reference** |  | **Reference** |  |

Table S15 Association between SES components and dentist visit behaviour.

|  |  | **Total** | |  | | **Males** | |  | | **Females** | |  |
| --- | --- | --- | --- | --- | --- | --- | --- | --- | --- | --- | --- | --- |
|  | **Dentist visit behaviour** | **Problem visit/Never/**  **Almost never** | | **Occasional visit** | | **Problem visit/Never/**  **Almost never** | | **Occasional visit** | | **Problem visit/Never/**  **Almost never** | | **Occasional visit** |
|  |  | **OR**  **(95% CI)**  ***(p-value)*** | | **OR**  **(95% CI)**  ***(p-value)*** | | **OR**  **(95% CI)**  ***(p-value)*** | | **OR**  **(95% CI)**  ***(p-value)*** | | **OR**  **(95% CI)**  ***(p-value)*** | | **OR**  **(95% CI)**  ***(p-value)*** |
| Primary care giver’s highest level of education  (n = 6039) | None or primary | **3.82 (2.67; 5.48)**  **(p < 0.001)** | | 0.95 (0.62; 1.45)  (p = 0.946) | | **5.13 (3.13; 8.39)**  **(p < 0.001)** | | 0.55 (0.25; 1.19)  (p = 0.129) | | **2.74 (1.61; 4.67)**  **(p < 0.001)** | | 1.26 (0.74; 2.15)  (p = 0.389) |
|  | Secondary | **1.62 (1.31; 1.99)**  **(p < 0.001)** | | 1.14 (0.97; 1.34)  (p = 0.119) | | **1.79 (1.34; 2.39)**  **(p < 0.001)** | | 1.17 (0.94; 1.46)  (p = 0.166) | | **1.44 (1.07; 1.94)**  **(p = 0.016)** | | 1.10 (0.87; 1.39)  (p = 0.442) |
|  | Tertiary | **Reference** | |  | | **Reference** | |  | | **Reference** | |  |
| Family income – quintiles  (n = 5586) | Lowest | **2.93 (2.19; 3.92)**  **(p < 0.001)** | | 1.24 (0.99; 1.54)  (p = 0.054) | | **2.78 (1.89; 4.07)**  **(p < 0.001)** | | 1.41 (0.84; 1.55)  (p = 0.399) | | **3.20 (2.05; 5.00)**  **(p < 0.001)** | | 1.35 (0.99; 1.85)  (p = 0.059) |
|  | 2^nd^ | **3.36 (2.52; 4.48)**  **(p < 0.001)** | | **1.33 (1.07; 1.65)**  **(p = 0.011)** | | **3.09 (2.11; 4.54)**  **(p < 0.001)** | | 1.27 (0.94; 1.72)  (p = 0.127) | | **3.78 (2.43; 5.88)**  **(p < 0.001)** | | **1.39 (1.02; 1.92)**  **(p = 0.038)** |
|  | 3^rd^ | **2.34 (1.74; 3.16)**  **(p < 0.001)** | | 1.13 (0.91; 1.42)  (p = 0.260) | | **1.92 (1.28; 2.88)**  **(p = 0.002)** | | 1.00 (0.73; 1.37)  (p = 0.990) | | **2.95 (1.89; 4.61)**  **(p < 0.001)** | | 1.28 (0.94; 1.75)  (p = 0.125) |
|  | 4^th^ | **2.02 (1.50; 2.74)**  **(p < 0.001)** | | 1.18 (0.95; 1.47)  (p = 0.136) | | **1.90 (1.29; 2.82)**  **(p = 0.001)** | | 1.09 (0.81; 1.46)  (p = 0.563) | | **2.18 (1.36; 3.51)**  **(p = 0.001)** | | 1.29 (0.94; 1.78)  (p = 0.116) |
|  | Highest | **Reference** | |  | | **Reference** | |  | | **Reference** | |  |
| Family class (Occupation)  (n = 5481) | Semi-skilled/ Unskilled manual | **1.68 (1.32; 2.14)**  **(p < 0.001)** | **1.25 (1.02; 1.54)**  **(p = 0.033)** | | 1.41 (0.99; 2.02)  (p = 0.056) | | 0.89 (0.64; 1.23)  (p = 0.481) | | **1.97 (1.40; 2.77)**  **(p < 0.001)** | | **1.63 (1.24; 2.16)**  **(p < 0.001)** | |
|  | Other non-manual/ Skilled manual | **1.81 (1.52; 2.16)**  **(p < 0.001)** | **1.27 (1.10; 1.48)**  **(p = 0.001)** | | **1.96 (1.54; 2.49)**  **(p < 0.001)** | | **1.25 (1.02; 1.54)**  **(p = 0.035)** | | **1.68 (1.29; 2.19)**  **(p < 0.001)** | | **1.30 (1.05; 1.62)**  **(p = 0.015)** | |
|  | Professional managers | **Reference** |  | | **Reference** | |  | | **Reference** | |  | |
| Medical card status  (n = 6039) | Yes, full card | **2.10 (1.80; 2.45)**  **(p < 0.001)** | 1.10 (0.96; 1.27)  (p = 0.175) | | **2.04 (1.65; 2.52)**  **(p < 0.001)** | | 0.96 (0.78; 1.18)  (p = 0.717) | | **2.18 (1.75; 2.72)**  **(p < 0.001)** | | **1.24 (1.02; 1.51)**  **(p = 0.030)** | |
|  | Yes, doctor only card | **2.89 (1.89; 4.40)**  **(p < 0.001)** | 1.49 (0.98; 2.28)  (p = 0.063) | | **3.46 (1.91; 6.27)**  **(p < 0.001)** | | 1.71 (0.92; 3.15)  (p = 0.088) | | **2.46 (1.35; 4.49)**  **(p = 0.003)** | | 1.34 (0.74; 2.41)  (p = 0.332) | |
|  | Not covered | **Reference** |  | | **Reference** | |  | | **Reference** | |  | |

Table S16 Association between SES components and sugary foods intake

|  |  | **Total** |  | **Males** |  | **Females** |  |
| --- | --- | --- | --- | --- | --- | --- | --- |
|  | **Sugary foods intake** | **More than once a day** | **Once a day** | **More than once a day** | **Once a day** | **More than once a day** | **Once a day** |
|  |  | **OR**  **(95% CI)**  ***(p-value)*** | **OR**  **(95% CI)**  ***(p-value)*** | **OR**  **(95% CI)**  ***(p-value)*** | **OR**  **(95% CI)**  ***(p-value)*** | **OR**  **(95% CI)**  ***(p-value)*** | **OR**  **(95% CI)**  ***(p-value)*** |
| Primary care giver’s highest level of education  (n = 6039) | None or primary | **1.69 (1.09; 2.62)**  **(p = 0.019)** | 1.43 (1.00; 2.03)  (p = 0.048) | 1.15 (0.61; 2.14)  (p = 0.666) | 1.26 (0.78; 2.03)  (p = 0.349) | **2.53 (1.34; 4.78)**  **(p = 0.004)** | 1.63 (0.96; 2.78)  (p = 0.070) |
|  | Secondary | **1.30 (1.08; 1.57)**  **(p = 0.006)** | 0.96 (0.83; 1.11)  (p = 0.599) | **1.38 (1.07; 1.78)**  **(p = 0.012)** | 1.21 (0.99; 1.47)  (p = 0.052) | 1.24 (0.93; 1.65)  (p = 0.139) | **0.75 (0.61; 0.92)**  **(p = 0.007)** |
|  | Tertiary | **Reference** |  | **Reference** |  | **Reference** |  |
| Family income – quintiles  (n = 5586) | Lowest | **1.49 (1.17; 1.89)**  **(p = 0.001)** | 0.96 (0.79; 1.16)  (p = 0.673) | **1.74 (1.24; 2.43)**  **(p = 0.001)** | 1.21 (0.92; 1.59)  (p = 0.164) | 1.29 (0.91; 1.82)  (p = 0.147) | 0.76 (0.58; 1.00)  (p = 0.053) |
|  | 2^nd^ | 1.28 (0.99; 1.64)  (p = 0.051) | 1.04 (0.86; 1.26)  (p = 0.689) | **1.59 (1.12; 2.24)**  **(p = 0.009)** | 1.26 (0.96; 1.66)  (p = 0.093) | 1.03 (0.72; 1.47)  (p = 0.863) | 0.86 (0.65; 1.13)  (p = 0.282) |
|  | 3^rd^ | 1.17 (0.91; 1.51)  (p = 0.205) | 1.05 (0.86; 1.27)  (p = 0.642) | 1.42 (1.00; 2.02)  (p = 0.048) | 1.12 (0.85; 1.48)  (p = 0.413) | 0.97 (0.68; 1.40)  (p = 0.904) | 0.97 (0.74; 1.28) (p = 0.855) |
|  | 4^th^ | 1.16 (0.91; 1.50) (p = 0.233) | 1.16 (0.95; 1.41)  (p = 0.134) | 1.09 (0.78; 1.54)  (p = 0.590) | 1.13 (0.87; 1.46)  (p = 0.366) | 1.26 (0.87; 1.84)  (p = 0.218) | 1.21 (0.90; 1.62)  (p = 0.192) |
|  | Highest | **Reference** |  | **Reference** |  | **Reference** |  |
| Family class (Occupation)  (n = 5481) | Semi-skilled/ Unskilled manual | 1.12 (0.89; 1.41)  (p = 0.337) | **0.71 (0.59; 0.85)**  **(p < 0.001)** | 1.24 (0.89; 1.72)  (p = 0.205) | 0.78 (0.59; 1.03)  (p = 0.089) | 1.06 (0.77; 1.46)  (p = 0.727) | **0.65 (0.51; 0.84)**  **(p = 0.001)** |
|  | Other non-manual/ Skilled manual | **1.37 (1.15; 1.62)**  **(p < 0.001)** | 0.99 (0.86; 1.13)  (p = 0.858) | **1.32 (1.04; 1.66)**  **(p = 0.021)** | 0.94 (0.78; 1.14)  (p = 0.540) | **1.44 (1.13; 1.85)**  **(p = 0.004)** | 1.04 (0.85; 1.26)  (p = 0.718) |
|  | Professional managers | **Reference** |  | **Reference** |  | **Reference** |  |
| Medical card status  (n = 6039) | Yes, full card | **1.28 (1.09; 1.49)**  **(p = 0.002)** | 0.92 (0.81; 1.04)  (p = 0.177) | 1.20 (0.97; 1.49)  (p = 0.096) | 0.99 (0.83; 1.18) (p = 0.909) | **1.38 (1.11; 1.71)**  **(p = 0.004)** | 0.86 (0.72; 1.02)  (p = 0.082) |
|  | Yes, doctor only card | 1.22 (0.74; 2.01)  (p = 0.424) | 1.16 (0.78; 1.72)  (p = 0.471) | 1.56 (0.72; 3.39)  (p = 0.263) | 1.82 (0.97; 3.43)  (p = 0.062) | 1.08 (0.56; 2.08)  (p = 0.815) | 0.81 (0.48; 1.37)  (p = 0.437) |
|  | Not covered | **Reference** |  | **Reference** |  | **Reference** |  |

Table S17 Association between SES components and sugary drinks intake

|  |  | **Total** |  | **Males** |  | **Females** |  |
| --- | --- | --- | --- | --- | --- | --- | --- |
|  | **Sugary drinks intake** | **More than once a day** | **Once a day** | **More than once a day** | **Once a day** | **More than once a day** | **Once a day** |
|  |  | **OR**  **(95% CI)**  ***(p-value)*** | **OR**  **(95% CI)**  ***(p-value)*** | **OR**  **(95% CI)**  ***(p-value)*** | **OR**  **(95% CI)**  ***(p-value)*** | **OR**  **(95% CI)**  ***(p-value)*** | **OR**  **(95% CI)**  ***(p-value)*** |
| Primary care giver’s highest level of education  (n = 6039) | None or primary | **3.84 (2.61; 5.65)**  **(p < 0.001)** | **3.06 (2.17; 4.30)**  **(p < 0.001)** | **2.94 (1.64; 5.28)**  **(p < 0.001)** | **3.21 (1.95; 5.27)**  **(p < 0.001)** | **5.79 (3.39; 9.88)**  **(p < 0.001)** | **3.33 (2.03; 5.46)**  **(p < 0.001)** |
|  | Secondary | **1.73 (1.44; 2.09)**  **(p < 0.001)** | **1.44 (1.24; 1.67)**  **(p < 0.001)** | **1.38 (1.09; 1.76)**  **(p = 0.008)** | 1.21 (0.99; 1.47)  (p = 0.059) | **2.43 (1.79; 3.29)**  **(p < 0.001)** | **1.91 (1.51; 2.43)**  **(p < 0.001)** |
|  | Tertiary | **Reference** |  | **Reference** |  | **Reference** |  |
| Family income – quintiles  (n = 5586) | Lowest | **3.24 (2.53; 4.14)**  **(p < 0.001)** | **1.51 (1.24; 1.84)**  **(p < 0.001)** | **3.27 (2.35; 4.55)**  **(p < 0.001)** | 1.29 (0.99; 1.69)  (p = 0.062) | **3.33 (2.29; 4.85)**  **(p < 0.001)** | **2.03 (1.49; 2.76)**  **(p < 0.001)** |
|  | 2^nd^ | **2.39 (1.86; 3.08)**  **(p < 0.001)** | **1.52 (1.25; 1.85)**  **(p < 0.001)** | **1.78 (1.26; 2.52)**  **(p = 0.001)** | 1.24 (0.95; 1.61)  (p = 0.111) | **3.33 (2.28; 4.85)**  **(p < 0.001)** | **2.09 (1.54; 2.86)**  **(p < 0.001)** |
|  | 3^rd^ | **1.94 (1.50; 2.51)**  **(p < 0.001)** | **1.37 (1.13; 1.67)**  **(p = 0.002)** | **1.79 (1.26; 2.54)**  **(p = 0.001)** | 1.05 (0.79; 1.38)  (p = 0.734) | **2.22 (1.51; 3.27)**  **(p < 0.001)** | **2.09 (1.55; 2.83)**  **(p < 0.001)** |
|  | 4^th^ | **1.49 (1.14; 1.94)**  **(p = 0.003)** | **1.34 (1.10; 1.63)**  **(p = 0.003)** | 1.34 (0.94; 1.89)  (p = 0.104) | 1.20 (0.93; 1.55)  (p = 0.152) | **1.67 (1.11; 2.52)**  **(p = 0.014)** | **1.52 (1.10; 2.09)**  **(p = 0.010)** |
|  | Highest | **Reference** |  | **Reference** |  | **Reference** |  |
| Family class (Occupation)  (n = 5481) | Semi-skilled/ Unskilled manual | **2.76 (2.22; 3.43)**  **(p < 0.001)** | **1.74 (1.44; 2.10)**  **(p < 0.001)** | **3.42 (2.49; 4.71)**  **(p < 0.001)** | **2.16 (1.63; 2.87)**  **(p < 0.001)** | **2.64 (1.93; 3.61)**  **(p < 0.001)** | **1.70 (1.29; 2.22)**  **(p < 0.001)** |
|  | Other non-manual/ Skilled manual | **1.77 (1.50; 2.09)**  **(p < 0.001)** | **1.16 (1.01; 1.32)**  **(p = 0.033)** | **1.67 (1.34; 2.09)**  **(p < 0.001)** | 1.05 (0.87; 1.26)  (p = 0.601) | **2.00 (1.56; 2.56)**  **(p < 0.001)** | **1.40 (1.15; 1.72)**  **(p = 0.001)** |
|  | Professional managers | **Reference** |  | **Reference** |  | **Reference** |  |
| Medical card status  (n = 6039) | Yes, full card | **2.55 (2.21; 2.95)**  **(p < 0.001)** | **1.59 (1.40; 1.80)**  **(p < 0.001)** | **2.11 (1.72; 2.58)**  **(p < 0.001)** | **1.43 (1.20; 1.71)**  **(p < 0.001)** | **3.30 (2.68; 4.08)**  **(p < 0.001)** | **1.87 (1.56; 2.24)**  **(p < 0.001)** |
|  | Yes, doctor only card | 1.38 (0.87; 2.17)  (p = 0.169) | 0.99 (0.67; 1.48)  (p = 0.985) | 0.99 (0.50; 1.94)  (p = 0.967) | 0.77 (0.43; 1.37)  (p = 0.376) | **2.01 (1.08; 3.75)**  **(p = 0.027)** | 1.36 (0.78; 2.36)  (p = 0.278) |
|  | Not covered | **Reference** |  | **Reference** |  | **Reference** |  |

TABLE S18 Association between SES components and DEIS school status

|  |  | **Total** | **Males** | **Females** |
| --- | --- | --- | --- | --- |
|  | **DEIS School (YES)** | **OR**  **(95% CI)**  ***(p-value)*** | **OR**  **(95% CI)**  ***(p-value)*** | **OR**  **(95% CI)**  ***(p-value)*** |
| Primary care giver’s highest level of education  (n = 6039) | None or primary | **17.13 11.93; 24.59)**  **(p < 0.001)** | **19.81 (11.85; 33.11)**  **(p < 0.001)** | **17.43 (10.14; 29.98)**  **(p < 0.001)** |
|  | Secondary | **3.13 (2.48; 3.95)**  **(p < 0.001)** | **2.45 (1.83; 3.27)**  **(p < 0.001)** | **4.45 (3.01; 6.58)**  **(p < 0.001)** |
|  | Tertiary | **Reference** | **Reference** | **Reference** |
| Family income – quintiles  (n = 5586) | Lowest | **4.85 (3.65; 6.46)**  **(p < 0.001)** | **5.67 (3.72; 8.63)**  **(p < 0.001)** | **4.18 (2.83; 6.18)**  **(p < 0.001)** |
|  | 2^nd^ | **4.72 (3.55; 6.29)**  **(p < 0.001)** | **6.53 (4.29; 5.94)**  **(p < 0.001)** | **3.40 (2.29; 5.06)**  **(p = 0.033)** |
|  | 3^rd^ | **2.86 (2.12; 3.85)**  **(p < 0.001)** | **3.40 (2.18; 5.31)**  **(p < 0.001)** | **2.40 (1.61; 3.59)**  **(p < 0.001)** |
|  | 4^th^ | **2.45 (1.81; 3.31)**  **(p < 0.001)** | **3.58 (2.33; 5.49)**  **(p < 0.001)** | 1.53 (0.98; 2.38)  (p = 0.060) |
|  | Highest | **Reference** | **Reference** | **Reference** |
| Family class (Occupation)  (n = 5481) | Semi-skilled/ Unskilled manual | **5.10 (4.13; 6.29)**  **(p < 0.001)** | **4.35 (3.23; 5.85)**  **(p < 0.001)** | **6.20 (4.56; 4.45)**  **(p < 0.001)** |
|  | Other non-manual/ Skilled manual | **3.00 (2.52; 3.58)**  **(p < 0.001)** | **2.78 (2.20; 3.52)**  **(p < 0.001)** | **3.39 (2.59; 4.45)**  **(p < 0.001)** |
|  | Professional managers | **Reference** | **Reference** | **Reference** |
| Medical card status  (n = 6039) | Yes, full card | **3.19 (2.77; 3.68)**  **(p < 0.001)** | **3.13 (2.57; 3.81)**  **(p < 0.001)** | **3.27 (2.67; 4.02)**  **(p < 0.001)** |
|  | Yes, doctor only card | **2.78 (1.86; 4.15)**  **(p < 0.001)** | **3.29 (1.91; 5.68)**  **(p < 0.001)** | **2.32 (1.28; 4.21)**  **(p = 0.006)** |
|  | Not covered | **Reference** | **Reference** | **Reference** |

Table S19 Association between SES components and financial difficulties

|  |  | **Total** | **Males** | **Females** |
| --- | --- | --- | --- | --- |
|  | **Financial Difficulties (YES)** | **OR (95% CI)**  ***(p-value)*** | **OR (95% CI)**  ***(p-value)*** | **OR (95% CI)**  ***(p-value)*** |
| Primary care giver’s highest level of education  (n = 6039) | None or primary | **6.30 (4.28; 9.28)**  **(p < 0.001)** | **5.73 (3.35; 9.81)**  **(p < 0.001)** | **6.91 (3.95; 12.09)**  **(p < 0.001)** |
|  | Secondary | **1.95 (1.72; 2.21)**  **(p < 0.001)** | **1.82 (1.53; 2.16)**  **(p < 0.001)** | **2.09 (1.74; 2.52)**  **(p < 0.001)** |
|  | Tertiary | **Reference** | **Reference** | **Reference** |
| Family income – quintiles  (n = 5586) | Lowest | **13.00 (10.58; 15.98)**  **(p < 0.001)** | **13.34 (10.01; 17.78)**  **(p < 0.001)** | **12.59 (9.37; 16.92)**  **(p < 0.001)** |
|  | 2^nd^ | **9.98 (8.18; 12.16)**  **(p < 0.001)** | **10.28 (7.80; 13.55)**  **(p < 0.001)** | **9.62 (7.24; 12.78)**  **(p < 0.001)** |
|  | 3^rd^ | **4.25 (3.54; 5.09)**  **(p < 0.001)** | **3.80 (2.94; 4.91)**  **(p < 0.001)** | **4.63 (3.58; 5.99)**  **(p < 0.001)** |
|  | 4^th^ | **2.17 (1.82; 2.59)**  **(p < 0.001)** | **2.58 (2.03; 3.29)**  **(p < 0.001)** | **1.76 (1.35; 2.29)**  **(p < 0.001)** |
|  | Highest | **Reference** | **Reference** | **Reference** |
| Family class (Occupation)  (n = 5481) | Semi-skilled/ Unskilled manual | **3.15 (2.64; 3.77)**  **(p < 0.001)** | **3.34 (2.58; 4.34)**  **(p < 0.001)** | **2.94 (2.29; 3.76)**  **(p < 0.001)** |
|  | Other non-manual/ Skilled manual | **2.34 (2.11; 2.68)**  **(p < 0.001)** | **2.41 (2.04; 2.85)**  **(p < 0.001)** | **2.31 (1.95; 2.75)**  **(p < 0.001)** |
|  | Professional managers | **Reference** | **Reference** | **Reference** |
| Medical card status  (n = 6039) | Yes, full card | **5.08 (4.47; 5.78)**  **(p < 0.001)** | **4.43 (3.71; 5.28)**  **(p < 0.001)** | **5.84 (4.83; 7.05)**  **(p < 0.001)** |
|  | Yes, doctor only card | **3.24 (2.21; 4.75)**  **(p < 0.001)** | **3.79 (2.13; 6.77)**  **(p < 0.001)** | **2.81 (1.68; 4.70)**  **(p < 0.001)** |
|  | Not covered | **Reference** | **Reference** | **Reference** |

Table S20 Association between SES components and private medical insurance.

|  |  | **Total** | **Males** | **Females** |
| --- | --- | --- | --- | --- |
|  | **Private medical insurance (YES)** | **OR**  **(95% CI)**  ***(p-value)*** | **OR**  **(95% CI)**  ***(p-value)*** | **OR**  **(95% CI)**  ***(p-value)*** |
| Primary care giver’s highest level of education  (n = 6039) | None or primary | **26.12 (16.16; 42.21)**  **(p < 0.001)** | **17.59 (9.82; 31.53)**  **(p < 0.001)** | **48.36 (19.84; 117.88)**  **(p < 0.001)** |
|  | Secondary | **3.21 (2.79; 3.68)**  **(p < 0.001)** | **3.09 (2.56; 3.74)**  **(p < 0.001)** | **3.30 (2.71; 4.09)**  **(p < 0.001)** |
|  | Tertiary | **Reference** | **Reference** | **Reference** |
| Family income – quintiles  (n = 5586) | Lowest | **23.01 (18.40; 28.87)**  **(p < 0.001)** | **22.45 (16.40; 30.73)**  **(p < 0.001)** | **23.61 (17.07; 32.65)**  **(p < 0.001)** |
|  | 2^nd^ | **14.89 (11.98; 18.52)**  **(p < 0.001)** | **12.62 (9.34; 17.05)**  **(p < 0.001)** | **17.85 (12.99; 24.51)**  **(p < 0.001)** |
|  | 3^rd^ | **7.69 (6.22; 9.50)**  **(p < 0.001)** | **7.51 (5.56; 10.14)**  **(p < 0.001)** | **7.76 (5.75; 10.48)**  **(p < 0.001)** |
|  | 4^th^ | **3.25 (2.62; 4.02)**  **(p < 0.001)** | **3.27 (2.43; 4.39)**  **(p < 0.001)** | **3.24 (2.37; 4.44)**  **(p < 0.001)** |
|  | Highest | **Reference** | **Reference** | **Reference** |
| Family class (Occupation)  (n = 5481) | Semi-skilled/ Unskilled manual | **8.68 (7.18; 10.50)**  **(p < 0.001)** | **8.87 (6.75; 11.67)**  **(p < 0.001)** | **8.39 (6.44; 10.92)**  **(p < 0.001)** |
|  | Other non-manual/ Skilled manual | **4.15 (3.67; 4.69)**  **(p < 0.001)** | **3.81 (3.21; 4.52)**  **(p < 0.001)** | **4.64 (3.74; 5.33)**  **(p < 0.001)** |
|  | Professional managers | **Reference** | **Reference** | **Reference** |
| Medical card status  (n = 6039) | Yes, full card | **18.16 (15.63; 21.09)**  **(p < 0.001)** | **18.12 (14.71; 22.32)**  **(p < 0.001)** | **18.07 (14.56; 22.42)**  **(p < 0.001)** |
|  | Yes, doctor only card | **3.35 (2.38; 4.72)**  **(p < 0.001)** | **2.42 (1.48; 3.96)**  **(p < 0.001)** | **4.49 (2.75; 7.34)**  **(p < 0.001)** |
|  | Not covered | **Reference** | **Reference** | **Reference** |

Table S21 Association between SES components and private house ownership.

|  |  | **Total** | **Males** | **Females** |
| --- | --- | --- | --- | --- |
|  | **House ownership** | **Renter/Other** | **Renter/Other** | **Renter/Other** |
|  |  | **OR**  **(95% CI)**  ***(p-value)*** | **OR**  **(95% CI)**  ***(p-value)*** | **OR**  **(95% CI)**  ***(p-value)*** |
| Primary care giver’s highest level of education  (n = 6039) | None or primary | **8.10 (5.87; 11.18)**  **(p < 0.001)** | **7.52 (4.73; 11.96)**  **(p < 0.001)** | **8.52 (5.44; 13.36)**  **(p < 0.001)** |
|  | Secondary | **2.05 (1.69; 2.49)**  **(p < 0.001)** | **1.98 (1.50; 2.60)**  **(p < 0.001)** | **2.09 (1.60; 2.74)**  **(p < 0.001)** |
|  | Tertiary | **Reference** | **Reference** | **Reference** |
| Family income – quintiles  (n = 5586) | Lowest | **9.00 (6.68; 12.14)**  **(p < 0.001)** | **7.50 (4.95; 11.35)**  **(p < 0.001)** | **10.67 (6.92; 16.45)**  **(p < 0.001)** |
|  | 2^nd^ | **7.31 (5.41; 9.89)**  **(p < 0.001)** | **6.62 (4.36; 10.05)**  **(p < 0.001)** | **8.04 (5.19; 12.46)**  **(p < 0.001)** |
|  | 3^rd^ | **4.87 (3.58; 6.62)**  **(p < 0.001)** | **4.18 (2.71; 6.45)**  **(p < 0.001)** | **5.49 (3.53; 8.55)**  **(p < 0.001)** |
|  | 4^th^ | **1.59 (1.13; 2.25)**  **(p = 0.008)** | **1.61 (1.01; 2.57)**  **(p = 0.047)** | 1.58 (0.95; 2.64)  (p = 0.080) |
|  | Highest | **Reference** | **Reference** | **Reference** |
| Family class (Occupation)  (n = 5481) | Semi-skilled/ Unskilled manual | **7.58 (6.11; 9.41)**  **(p < 0.001)** | **7.28 (5.30; 9.99)**  **(p < 0.001)** | **7.57 (5.63; 10.17)**  **(p < 0.001)** |
|  | Other non-manual/ Skilled manual | **3.28 (2.71; 3.96)**  **(p < 0.001)** | **3.27 (2.48; 4.30)**  **(p < 0.001)** | **3.20 (2.46; 4.17)**  **(p < 0.001)** |
|  | Professional managers | **Reference** | **Reference** | **Reference** |
| Medical card status  (n = 6039) | Yes, full card | **9.95 (8.52; 11.61)**  **(p < 0.001)** | **10.34 (8.25; 12.95)**  **(p < 0.001)** | **9.49 (7.67; 11.74)**  **(p < 0.001)** |
|  | Yes, doctor only card | **5.14 (3.47; 7.63)**  **(p < 0.001)** | **2.32 (1.09; 4.91)**  **(p = 0.028)** | **7.95 (4.85; 13.04)**  **(p < 0.001)** |
|  | Not covered | **Reference** | **Reference** | **Reference** |

Table S22 Association between SES components and family composition.

|  |  | **Total** | **Males** | **Females** |
| --- | --- | --- | --- | --- |
|  | **Family structure** | **Single parent family** | **Single parent family** | **Single parent family** |
|  |  | **OR**  **(95% CI)**  ***(p-value)*** | **OR**  **(95% CI)**  ***(p-value)*** | **OR**  **(95% CI)**  ***(p-value)*** |
| Primary care giver’s highest level of education  (n = 6039) | None or primary | **3.34 (2.42; 4.61)**  **(p < 0.001)** | **3.59 (2.25; 5.70)**  **(p < 0.001)** | **3.07 (1.97; 4.80)**  **(p < 0.001)** |
|  | Secondary | **1.38 (1.16; 1.64)**  **(p < 0.001)** | **1.44 (1.13; 1.85)**  **(p = 0.004)** | **1.31 (1.02; 1.67)**  **(p = 0.032)** |
|  | Tertiary | **Reference** | **Reference** | **Reference** |
| Family income – quintiles  (n = 5586) | Lowest | **3.12 (2.44; 3.98)**  **(p < 0.001)** | **2.82 (2.01; 3.96)**  **(p < 0.001)** | **3.44 (2.41; 4.92)**  **(p < 0.001)** |
|  | 2^nd^ | **3.16 (2.47; 4.04)**  **(p < 0.001)** | **2.74 (1.95; 13.86)**  **(p < 0.001)** | **3.63 (2.54; 5.19)**  **(p < 0.001)** |
|  | 3^rd^ | **2.70 (2.10; 3.46)**  **(p < 0.001)** | **2.30 (1.62; 3.27)**  **(p < 0.001)** | **3.10 (2.17; 4.44)**  **(p < 0.001)** |
|  | 4^th^ | **1.40 (1.07; 1.83)**  **(p = 0.015)** | **1.61 (1.13; 2.29)**  **(p = 0.009)** | 1.12 (0.74; 1.71)  (p = 0.586) |
|  | Highest | **Reference** | **Reference** | **Reference** |
| Family class (Occupation)  (n = 5481) | Semi-skilled/ Unskilled manual | **4.14 (3.33; 5.16)**  **(p < 0.001)** | **4.11 (2.98; 5.67)**  **(p < 0.001)** | **4.05 (2.99; 5.47)**  **(p < 0.001)** |
|  | Other non-manual/ Skilled manual | **2.32 (1.93; 2.80)**  **(p < 0.001)** | **2.72 (2.09; 3.53)**  **(p < 0.001)** | **1.96 (1.50; 2.56)**  **(p < 0.001)** |
|  | Professional managers | **Reference** | **Reference** | **Reference** |
| Medical card status  (n = 6039) | Yes, full card | **6.40 (5.53; 7.40)**  **(p < 0.001)** | **6.63 (5.39; 8.15)**  **(p < 0.001)** | **6.14 (5.00; 7.52)**  **(p < 0.001)** |
|  | Yes, doctor only card | **2.61 (1.69; 4.02)**  **(p < 0.001)** | **2.02 (1.01; 4.07)**  **(p = 0.048)** | **3.08 (1.76; 5.40)**  **(p < 0.001)** |
|  | Not covered | **Reference** | **Reference** | **Reference** |

Table S23 Association between SES components and job stress.

|  |  | **Total** | **Males** | **Females** |
| --- | --- | --- | --- | --- |
|  | **Job stress (YES)** | **Stressed** | **Stressed** | **Stressed** |
|  |  | **OR**  **(95% CI)**  ***(p-value)*** | **OR**  **(95% CI)**  ***(p-value)*** | **OR**  **(95% CI)**  ***(p-value)*** |
| Primary care giver’s highest level of education  (n = 6039) | None or primary | **2.69 (1.76; 4.12)**  **(p < 0.001)** | **2.44 (1.35; 4.43)**  **(p = 0.003)** | **2.92 (1.58; 5.39)**  **(p < 0.001)** |
|  | Secondary | **0.86 (0.75; 0.99)**  **(p = 0.034)** | 0.85 (0.71; 1.03)  (p = 0.096) | 0.86 (0.71; 1.06)  (p = 0.162) |
|  | Tertiary | **Reference** | **Reference** | **Reference** |
| Family income – quintiles  (n = 5586) | Lowest | 0.89 (0.74; 1.07)  (p = 0.207) | 0.87 (0.67; 1.12)  (p = 0.280) | 0.90 (0.69; 1.19)  (p = 0.465) |
|  | 2^nd^ | **0.79 (0.66; 0.96)**  **(p = 0.016)** | **0.75 (0.58; 0.98)**  **(p = 0.032)** | 0.83 (0.63; 1.10)  (p = 0.194) |
|  | 3^rd^ | **0.78 (0.65; 0.94)**  **(p = 0.009)** | **0.72 (0.55; 0.93)**  **(p = 0.013)** | 0.83 (0.63; 1.08)  (p = 0.164) |
|  | 4^th^ | 0.83 (0.69; 1.00)  (p = 0.048) | 0.88 (0.68; 1.13)  (p = 0.313) | 0.77 (0.58; 1.02)  (p = 0.070) |
|  | Highest | **Reference** | **Reference** | **Reference** |
| Family class (Occupation)  (n = 5481) | Semi-skilled/ Unskilled manual | 0.85 (0.71; 1.02)  (p = 0.078) | 0.78 (0.60; 1.00)  (p = 0.051) | 0.89 (0.69; 1.14)  (p = 0.357) |
|  | Other non-manual/ Skilled manual | 0.94 (0.83; 1.07)  (p = 0.360) | 1.01 (0.85; 1.21)  (p = 0.866) | 0.85 (0.71; 1.02)  (p = 0.088) |
|  | Professional managers | **Reference** | **Reference** | **Reference** |
| Medical card status  (n = 6039) | Yes, full card | 0.95 (0.85; 1.07)  (p = 0.419) | 0.98 (0.83; 1.15)  (p = 0.791) | 0.92 (0.78; 1.09)  (p = 0.325) |
|  | Yes, doctor only card | 0.81 (0.57; 1.16)  (p = 0.261) | **0.46 (0.28; 0.75)**  **(p = 0.002)** | 1.56 (0.88; 2.78)  (p = 0.128) |
|  | Not covered | **Reference** | **Reference** | **Reference** |

Table S24 Association between SES components and depression status

|  |  | **Total** | **Males** | **Females** |
| --- | --- | --- | --- | --- |
|  | **Depression status** | **Depressed** | **Depressed** | **Depressed** |
|  |  | **OR**  **(95% CI)**  ***(p-value)*** | **OR**  **(95% CI)**  ***(p-value)*** | **OR**  **(95% CI)**  ***(p-value)*** |
| Primary care giver’s highest level of education  (n = 6039) | None or primary | **2.37 (1.59; 3.53)**  **(p < 0.001)** | **2.95 (1.76; 4.95)**  **(p < 0.001)** | 1.85 (0.97; 3.54)  (p = 0.062) |
|  | Secondary | **1.32 (1.07; 1.63)**  **(p = 0.011)** | 1.12 (0.85; 1.48)  (p = 0.422) | **1.64 (1.17; 2.30)**  **(p = 0.004)** |
|  | Tertiary | **Reference** | **Reference** | **Reference** |
| Family income – quintiles  (n = 5586) | Lowest | **3.00 (2.22; 4.06)**  **(p < 0.001)** | **2.22 (1.50; 3.29)**  **(p < 0.001)** | **4.61 (2.80; 7.61)**  **(p < 0.001)** |
|  | 2^nd^ | **2.61 (1.92; 3.55)**  **(p < 0.001)** | **2.54 (1.73; 3.74)**  **(p < 0.001)** | **2.91 (1.73; 4.91)**  **(p < 0.001)** |
|  | 3^rd^ | **2.46 (1.80; 3.35)**  **(p < 0.001)** | **1.73 (1.14; 2.61)**  **(p = 0.009)** | **3.91 (2.36; 6.48)**  **(p < 0.001)** |
|  | 4^th^ | 1.12 (0.79; 1.59)  (p = 0.516) | 0.95 (0.61; 1.47)  (p = 0.810) | 1.46 (0.82; 2.62)  (p = 0.199) |
|  | Highest | **Reference** | **Reference** | **Reference** |
| Family class (Occupation)  (n = 5481) | Semi-skilled/ Unskilled manual | **2.11 (1.63; 2.74)**  **(p < 0.001)** | **2.58 (1.83; 3.65)**  **(p < 0.001)** | **1.77 (1.19; 2.62)**  **(p < 0.001)** |
|  | Other non-manual/ Skilled manual | **1.99 (1.63; 2.43)**  **(p < 0.001)** | **1.91 (1.46; 2.50)**  **(p < 0.001)** | **2.12 (1.57; 2.85)**  **(p < 0.001)** |
|  | Professional managers | **Reference** | **Reference** | **Reference** |
| Medical card status  (n = 6039) | Yes, full card | **2.71 (2.30; 3.20)**  **(p < 0.001)** | **2.61 (2.08; 3.27)**  **(p < 0.001)** | **2.88 (2.26; 3.67)**  **(p < 0.001)** |
|  | Yes, doctor only card | 1.55 (0.90; 2.66)  (p = 0.110) | 1.71 (0.82; 3.57)  (p = 0.149) | 1.42 (0.64; 3.16)  (p = 0.389) |
|  | Not covered | **Reference** | **Reference** | **Reference** |

Table S25 Association between SES components and parental stress scale.

|  |  | **Total** |  | **Males** |  | **Females** |  |
| --- | --- | --- | --- | --- | --- | --- | --- |
|  |  | **Mean (SD)** | **P value** | **Mean (SD)** | **P value** | **Mean (SD)** | **P value** |
| Primary care giver’s highest level of education  (n = 5957  M = 3045  F = 2929) | None or primary | 11.41 (5.34) | < 0.001 | 11.67 (5.97) | < 0.001 | 11.16 (4.68) | < 0.001 |
|  | Secondary | 10.23 (4.18) |  | 10.26 (4.24) |  | 10.21 (4.12) |  |
|  | Tertiary | 10.33 (3.90) |  | 10.52 (4.17) |  | 10.11 (3.55) |  |
| Family income – quintiles  (n = 5549  M = 2721  F = 2721) | Lowest | 10.03 (3.93) | < 0.001 | 9.93 (3.76) | 0.019 | 10.12 (4.10) | < 0.001 |
|  | 2^nd^ | 10.76 (4.64) |  | 10.82 (4.75) |  | 10.70 (4.53) |  |
|  | 3^rd^ | 10.39 (4.01) |  | 10.36 (4.54) |  | 10.41 (3.83) |  |
|  | 4^th^ | 10.20 (4.18) |  | 10.38 (4.54) |  | 9.98 (3.65) |  |
|  | Highest | 10.12 (4.09) |  | 10.41 (4.26) |  | 9.78 (3.87 |  |
| Family class (Occupation)  (n = 5429  M = 2800  F = 2629) | Semi-skilled/ Unskilled manual | 10.68 (4.72) | < 0.001 | 11.08 (4.89) | < 0.001 | 10.35 (4.55) | 0.264 |
|  | Other non-manual/ Skilled manual | 10.24 (3.96) |  | 10.32 (4.03) |  | 10.17 (3.89) |  |
|  | Professional managers | 10.03 (3.98) |  | 10.05 (4.04) |  | 10.00 (3.89) |  |
| Medical card status  (n = 5974  M = 3045  F = 2929) | Yes, full card | 10.93 (4.55) | < 0.001 | 10.98 (4.74) | < 0.001 | 10.89 (4.37) | < 0.001 |
|  | Yes, doctor only card | 9.52 (3.24) |  | 8.9 (2.49) |  | 10.06 (3.72) |  |
|  | Not covered | 9.98 (3.94) |  | 10.10 (4.07) |  | 9.83 (3.79) |  |

Table S26 Association between PCGs’ highest educational level and young males’ self-reported oral health (self-rated oral health) adjusted for covariates (wave 1), behavioural factors, material factors and psychosocial factors (logistic regression odds ratios (95% CI) for self-rated sub-optimal oral health). Model 2: Model 1 + behavioural factors; Model 3: Model 1 + material factors; Model 4: Model 1 + Psychosocial factors and Model 5: Model 1 + behavioural factors + material factors + Psychosocial factors. All models were adjusted for the ‘area of residence’, the ‘main language spoken at home’ and ‘PCGs’ country of birth’. OR: Odds Ratio; NA (not applicable) refers to a variable that was excluded from the model using stepwise backward selection.

|  |  | **Model 1 (Crude)** | **Model 2** | **Model 3** | **Model 4** | **Model 5** |
| --- | --- | --- | --- | --- | --- | --- |
|  |  | **OR (95% CI)** | **OR (95% CI)**  **[% change]** | **OR (95% CI)**  **[% change]** | **OR (95% CI)**  **[% change]** | **OR (95% CI)**  **[% change]** |
| **PCG highest level of education** | None or primary | **2.31 (1.29; 4.13)** | **2.02 (1.10; 3.68) [22.14]** | 0.95 (0.46; 1.97) [100.00] | 1.57 (0.78; 3.15)  [56.49] | 1.16 (0.56; 2.39)  [87.79] |
|  | Secondary | 1.33 (0.98; 1.81) | 1.14 (0.83; 1.58)  [57.58] | 1.00 (0.72; 1.39)  [100.00] | 1.29 (0.94; 1.78)  [12.12] | 0.92 (0.65; 1.31)  [100.00] |
|  | Tertiary (Reference) | **1** | 1 | 1 | 1 | 1 |
| ***Behavioural factors*** | **Toothbrushing frequency** |  |  |  |  |  |
|  | Less than once /Rarely/Never |  | **4.69 (3.28; 6.69)** |  |  | **4.67 (3.23; 6.81)** |
|  | Once |  | **2.01 (1.53; 2.65)** |  |  | **2.05 (1.53; 2.75)** |
|  | Twice/More than twice (Reference) |  | 1 |  |  | 1 |
|  | **Dentist visit behaviour** |  |  |  |  |  |
|  | Problem/Never |  | **1.91 (1.40; 2.60)** |  |  | **1.67 (1.19; 2.34)** |
|  | Occasional visit |  | 0.75 (0.52; 1.07) |  |  | 0.73 (0.50; 1.07) |
|  | At least a year (Reference) |  | 1 |  |  | 1 |
|  | **Sugary drinks intake** |  |  |  |  |  |
|  | More than once a day |  | 1.16 (0.83; 1.62) |  |  | NA |
|  | Once a day |  | 1.06 (0.79; 1.41) |  |  | NA |
|  | Not at all (Reference) |  | 1 |  |  | 1 |
|  | **Sugary foods intake** |  |  |  |  |  |
|  | More than once a day |  | 1.34 (0.96; 1.89) |  |  | **1.58 (1.11; 2.23)** |
|  | Once a day |  | 0.71 (0.53; 0.95) |  |  | 0.84 (0.61; 1.16) |

Table S26 Continued….

| ***Behavioural factors*** | **Sugary foods intake** | **OR (95% CI)** | **OR (95% CI)**  **[% change]** | **OR (95% CI)**  **[% change]** | **OR (95% CI)**  **[% change]** | **OR (95% CI)**  **[% change]** |
| --- | --- | --- | --- | --- | --- | --- |
|  | Not at all (Reference) |  | 1 |  |  | 1 |
| ***Material factors*** | **Financial difficulties** |  |  |  |  |  |
|  | With difficulty |  | 1.04 (0.79; 1.37) |  |  | NA |
|  | No difficulty (Reference) |  | 1 |  |  |  |
|  | **DEIS school** |  |  |  |  |  |
|  | Yes |  | 1.03 (0.75; 1.42) |  |  | NA |
|  | No (Reference) |  | 1 |  |  |  |
|  | **Homeownership** |  |  |  |  |  |
|  | Renters/Rent free /Others |  | 1.23 (0.88; 1.72) |  |  | NA |
|  | Owners (Reference) |  | 1 |  |  |  |
|  | **Private medical insurance** |  |  |  |  |  |
|  | No |  | **1.84 (1.37; 2.46)** |  |  | **1.57 (1.19; 2.09)** |
|  | Yes (Reference) |  | 1 |  |  | 1 |
| ***Psychosocial factors*** | **PCG parental stressor scale score** |  |  |  | **1.04 (1.02; 1.07)** | 1.03 (1.00; 1.06) |
|  | **PCG depression status** |  |  |  | 1 | 1 |
|  | Depressed |  |  |  | 0.88 (0.60; 1.30) | 0.69 (0.45; 1.07) |
|  | Not depressed (Reference) |  |  |  | 1 | 1 |
|  | **Family structure** |  |  |  |  |  |
|  | Single parent family |  |  |  | 1.25 (0.92; 1.70) | NA |
|  | Couple family (Reference) |  |  |  | 1 |  |
|  | **Job stress** |  |  |  |  |  |
|  | Stressed |  |  |  | 1.01 (0.78; 1.32) | NA |
|  | Not stressed (Reference) |  |  |  | 1 |  |

Bold = significant ORs (95% CI does not include 1).

Table S27 Association between household/family income and young males’ self-reported oral health (self-rated oral health) adjusted for covariates (wave 1), behavioural factors, material factors and psychosocial factors (logistic regression odds ratios (95% CI) for self-rated sub-optimal oral health). Model 2: Model 1 + behavioural factors; Model 3: Model 1 + material factors; Model 4: Model 1 + Psychosocial factors and Model 5: Model 1 + behavioural factors + material factors + Psychosocial factors. All models were adjusted for the ‘area of residence’, the ‘main language spoken at home’ and ‘PCGs’ country of birth’. OR: Odds Ratio; NA (not applicable) refers to a variable that was excluded from the model using stepwise backward selection.

|  |  | **Model 1 (Crude)** | **Model 2** | **Model 3** | **Model 4** | **Model 5** |
| --- | --- | --- | --- | --- | --- | --- |
|  |  | **OR (95% CI)**  **[% change]** | **OR (95% CI)**  **[% change]** | **OR (95% CI)**  **[% change]** | **OR (95% CI)**  **[% change]** | **OR (95% CI)**  **[% change]** |
| **Household/Family**  **Income quintiles** | Lowest | **1.72 (1.16; 2.56)** | 1.22 (0.80; 1.86) [69.44] | 1.02 (0.64; 1.65) [97.22] | **1.68 (1.11; 2.53) [5.56]** | 0.80 (0.49; 1.28) [100.00] |
|  | 2^nd^ | **1.91 (1.29; 2.82)** | 1.46 (0.97; 2.20) [49.45] | 1.18 (0.74; 1.88) [80.22] | **1.65 (1.10; 2.50)** [28.57] | 0.90 (0.56; 1.43) [100.00] |
|  | 3^rd^ | 1.25 (0.82; 1.91) | 0.94 (0.60; 1.47) [100.00] | 0.89 (0.55; 1.43) [100.00] | 1.27 (0.82; 1.97) [-8.00] | 0.67 (0.41; 1.09) [100.00] |
|  | 4^th^ | 1.08 (0.71; 1.63) | 0.98 (0.64; 1.50) [100.00] | 0.85 (0.55; 1.33) [100.00] | 0.97 (0.63; 1.50) [100.00] | 0.75 (0.47; 1.19) [100.00] |
|  | Highest (Reference) | 1 | 1 | 1 | 1 | 1 |
| ***Behavioural factors*** | **Toothbrushing frequency** |  |  |  |  |  |
|  | Less than once/Rarely/  Never |  | **4.57 (3.18; 6.57)** |  |  | **4.72 (3.22; 6.90)** |
|  | Once |  | **2.05 (1.54; 2.72)** |  |  | **2.09 (1.55; 2.81)** |
|  | Twice/More than twice (Reference) |  | 1 |  |  | 1 |
|  | **Dentist visit behaviour** |  |  |  |  |  |
|  | Problem/Never |  | **1.72 (1.25; 2.36)** |  |  | **1.48 (1.05; 2.08)** |
|  | Occasional visit |  | **0.64 (0.43; 0.93)** |  |  | **0.61 (0.41; 0.92)** |
|  | At least a year (Reference) |  | 1 |  |  | 1 |
|  | **Sugary drinks intake** |  |  |  |  |  |
|  | More than once a day |  | 1.22 (0.87; 1.72) |  |  | NA |
|  | Once a day |  | 1.13 (0.84; 1.52) |  |  | NA |
|  | Not at all (Reference) |  | 1 |  |  |  |
|  | **Sugary foods intake** |  |  |  |  |  |
|  | More than once a day |  | 1.31 (0.92; 1.85) |  |  | **1.62 (1.13; 2.31)** |

Table S27 Continued….

| ***Behavioural factors*** | **Sugary foods intake** | **OR (95% CI)**  **[% change]** | **OR (95% CI)**  **[% change]** | **OR (95% CI)**  **[% change]** | **OR (95% CI)**  **[% change]** | **OR (95% CI)**  **[% change]** |
| --- | --- | --- | --- | --- | --- | --- |
|  | Once a day |  | 0.73 (0.54; 1.00) |  |  | 0.89 (0.64; 1.23) |
|  | Not at all (Reference) |  | 1 |  |  |  |
| **Material factors** | **Financial difficulties** |  |  |  |  |  |
|  | With difficulty |  |  | 1.00 (0.74; 1.35) |  | NA |
|  | No difficulty (Reference) |  |  | 1 |  |  |
|  | **DEIS school** |  |  |  |  |  |
|  | Yes |  |  | 0.97 (0.70; 1.35) |  | NA |
|  | No (Reference) |  |  | 1 |  |  |
|  | **Homeownership** |  |  |  |  |  |
|  | Renters/Rent free/Others |  |  | 1.22 (0.87; 1.71) |  | NA |
|  | Owners (Reference) |  |  | 1 |  |  |
|  | **Private medical insurance** |  |  |  |  |  |
|  | No |  |  | **1.80 (1.31; 2.47)** |  | **1.67 (1.22; 2.29)** |
|  | Yes (Reference) |  |  | 1 |  | 1 |
| **Psychosocial factors** | **PCG Parental Stressor scale score** |  |  |  | **1.04 (1.01; 1.07)** | 1.03 (1.00; 1.06) |
|  | **PCG depression status** |  |  |  | 1 | 1 |
|  | Depressed |  |  |  | 0.82 (0.55; 1.22) | NA |
|  | Not depressed (Reference) |  |  |  | 1 |  |
|  | **Family structure** |  |  |  |  |  |
|  | Single parent family |  |  |  | 1.19 (0.87; 1.62) | NA |
|  | Couple family (Reference) |  |  |  | 1 |  |
|  | **Job stress** |  |  |  |  |  |
|  | Stressed |  |  |  | 1.07 (0.82; 1.40) | NA |
|  | Not stressed (Reference) |  |  |  | 1 |  |

Bold = significant ORs (95% CI does not include 1).

Table S28 Association between PCGs’ highest educational level and young females’ parent-reported oral health (number of teeth with dental fillings) adjusted for covariates (wave 1), behavioural factors, material factors and psychosocial factors (logistic regression odds ratios (95% CI) for having two teeth with dental fillings). Model 2: Model 1 + behavioural factors; Model 3: Model 1 + material factors; Model 4: Model 1 + Psychosocial factors and Model 5: Model 1 + behavioural factors + material factors + Psychosocial factors. All models were adjusted for the ‘area of residence’, the ‘main language spoken at home’ and ‘PCGs’ country of birth’. OR: Odds Ratio; NA (not applicable) refers to a variable that was excluded from the model using stepwise backward selection.

|  |  | **Model 1** | **Model 2** | **Model 3** | **Model 4** | **Model 5** |
| --- | --- | --- | --- | --- | --- | --- |
|  |  | **OR (95% CI)** | **OR (95% CI)**  **[% change]** | **OR (95% CI)**  **[% change]** | **OR (95% CI)**  **[% change]** | **OR (95% CI)**  **[% change]** |
| **PCG highest level of education** | None or primary | **3.34 (1.99; 5.60)** | **3.41 (2.01; 5.78)**  [-2.99] | **2.92 (1.63; 5.24)**  [17.95] | **3.28 (1.87; 5.76)**  [2.56] | **3.88 (2.18; 6.89)**  [-23.08] |
|  | Secondary | **1.47 (1.12; 1.93)** | **1.48 (1.12; 1.95)**  [-2.13] | **1.52 (1.13; 2.04)**  [-10.6] | **1.49 (1.13; 1.97)**  [-4.26] | **1.68 (1.25; 2.25)**  [-44.68] |
|  | Tertiary (Reference) | 1 | 1 | 1 | 1 | 1 |
| **Behavioural factors** | **Toothbrushing frequency** |  |  |  |  |  |
|  | Less than once/Rarely/Never |  | 1.27 (0.72; 2.23) |  |  | 1.28 (0.69; 2.36) |
|  | Once |  | 0.97 (0.72; 1.30) |  |  | 0.89 (0.65; 1.22) |
|  | Twice/More than twice (Reference) |  | 1 |  |  | 1 |
|  | **Dentist visit behaviour** |  |  |  |  |  |
|  | Problem/Never |  | **0.51 (0.37; 0.72)** |  |  | **0.49 (0.34; 0.70)** |
|  | Occasional visit |  | 0.90 (0.69; 1.17) |  |  | 0.87 (0.66; 1.14) |
|  | At least a year (Reference) |  | 1 |  |  | 1 |
|  | **Sugary drinks intake** |  |  |  |  |  |
|  | More than once a day |  | 1.09 (0.80; 1.47) |  |  | NA |
|  | Once a day |  | **1.51 (1.19; 1.92)** |  |  | NA |
|  | Not at all (Reference) |  | 1 |  |  |  |
|  | **Sugary foods intake** |  |  |  |  |  |
|  | More than once a day |  | 0.98 (0.72; 1.35) |  |  | 0.86 (0.62; 1.19) |
|  | Once a day |  | 1.26 (0.99; 1.59) |  |  | 1.23 (0.96; 1.57) |
|  | Not at all (Reference) |  | 1 |  |  | 1 |
| **Material factors** | **Financial difficulties** |  |  |  |  |  |
|  | With difficulty |  |  | 1.04 (0.82; 1.31) |  | 1.09 (0.87; 1.37) |
|  | No difficulty (Reference) |  |  | 1 |  | 1 |

Table S28 Continued…..

|  |  | **OR (95% CI)** | **OR (95% CI)**  **[% change]** | **OR (95% CI)**  **[% change]** | **OR (95% CI)**  **[% change]** | **OR (95% CI)**  **[% change]** |
| --- | --- | --- | --- | --- | --- | --- |
| **Material factors** | **DEIS school** |  |  |  |  |  |
|  | Yes |  |  | 0.96 (0.72; 1.26) |  | NA |
|  | No (Reference) |  |  | 1 |  |  |
|  | **Homeownership** |  |  |  |  |  |
|  | Renters/Rent free/Others |  |  | 1.27 (0.96; 1.68) |  | NA |
|  | Owners (Reference) |  |  | 1 |  |  |
|  | **Private medical insurance** |  |  |  |  |  |
|  | No |  |  | 1.04 (0.82; 1.33) |  |  |
|  | Yes (Reference) |  |  | 1 |  |  |
| **Psychosocial factors** | **PCGs’ mean parental stressor scale score** |  |  |  | **1.04 (1.01; 1.06)** | **1.05 (1.02; 1.08)** |
|  | **PCG depression status** |  |  |  |  |  |
|  | Depressed |  |  |  | 0.86 (0.60; 1.21) | NA |
|  | Not depressed (Reference) |  |  |  | 1 |  |
|  | **Family structure** |  |  |  |  |  |
|  | Single parent family |  |  |  | 1.14 (0.87; 1.49) | NA |
|  | Couple family (Reference) |  |  |  | 1 |  |
|  | **Job stress** |  |  |  |  |  |
|  | Stressed |  |  |  | 0.95 (0.75; 1.19) | 0.91 (0.72; 1.15) |
|  | Not stressed (Reference) |  |  |  | 1 | 1 |

Table S29 Association between PCGs’ highest educational level and young females’ parent-reported oral health (number of teeth with dental fillings) adjusted for covariates (wave 1), behavioural factors, material factors and psychosocial factors (logistic regression odds ratios (95% CI) for having three or more teeth with dental fillings). ). Model 2: Model 1 + behavioural factors; Model 3: Model 1 + material factors; Model 4: Model 1 + Psychosocial factors and Model 5: Model 1 + behavioural factors + material factors + Psychosocial factors. All models were adjusted for the ‘area of residence’, the ‘main language spoken at home’ and ‘PCGs’ country of birth’. OR: Odds Ratio; NA (not applicable) refers to a variable that was excluded from the model using stepwise backward selection.

|  |  | **Model 1** | **Model 2** | **Model 3** | **Model 4** | **Model 5** |
| --- | --- | --- | --- | --- | --- | --- |
|  |  | **OR (95% CI)** | **OR (95% CI)**  **[% change]** | **OR (95% CI)**  **[% change]** | **OR (95% CI)**  **[% change]** | **OR (95% CI)**  **[% change]** |
| **PCGs’ highest level of education** | None/primary | **1.91 (1.07; 3.43)** | **1.87 (1.03; 3.40)**  [4.40] | 1.81 (0.97; 3.38)  [10.99] | **2.11 (1.14; 3.89)**  [-21.98] | **1.93 (1.03; 3.60)**  [-2.20] |
|  | Secondary | 1.28 (0.98; 1.67) | 1.23 (0.94; 1.62)  [17.86] | 1.15 (0.86; 1.53)  [46.43] | 1.22 (0.93; 1.61)  [21.43] | 1.21 (0.91; 1.61)  [25.00] |
|  | Tertiary (Reference) | 1 | 1 | 1 | 1 | 1 |
| **Behavioural factors** | **Toothbrushing frequency** |  |  |  |  |  |
|  | Less than once/Rarely/Never |  | **2.43 (1.49; 3.98)** |  |  | **2.32 (1.35; 4.00)** |
|  | Once |  | **1.54 (1.18; 2.02)** |  |  | **1.45 (1.09; 1.95)** |
|  | Twice/More than twice (Reference) |  | 1 |  |  | 1 |
|  | **Dentist visit behaviour** |  |  |  |  |  |
|  | Problem/Never |  | **0.56 (0.40; 0.79)** |  |  | **0.56 (0.39; 0.80)** |
|  | Occasional visit |  | 0.83 (0.64; 1.09) |  |  | 0.75 (0.56; 1.01) |
|  | At least a year (Reference) |  | 1 |  |  | 1 |
|  | **Sugary drinks intake** |  |  |  |  |  |
|  | More than once a day |  | 1.10 (0.82; 1.48) |  |  | NA |
|  | Once a day |  | 1.13 (0.87; 1.46) |  |  | NA |
|  | Not at all (Reference) |  | 1 |  |  |  |
|  | **Sugary foods intake** |  |  |  |  |  |
|  | More than once a day |  | **1.59 (1.18; 2.14)** |  |  | **1.56 (1.14; 2.14)** |
|  | Once a day |  | 1.20 (0.94; 1.54) |  |  | 1.25 (0.96; 1.63) |
|  | Not at all (Reference) |  | 1 |  |  | 1 |
| **Material factors** | **Financial difficulties** |  |  |  |  |  |
|  | With difficulty |  |  | **1.45 (1.14; 1.85)** |  | **1.46 (1.15; 1.86)** |
|  | No difficulty (Reference) |  |  | 1 |  | 1 |

Table S29 Continued……

|  |  | **OR (95% CI)** | **OR (95% CI)**  **[% change]** | **OR (95% CI)**  **[% change]** | **OR (95% CI)**  **[% change]** | **OR (95% CI)**  **[% change]** |
| --- | --- | --- | --- | --- | --- | --- |
| **Material factors** | **DEIS school** |  |  |  |  |  |
|  | Yes |  |  | 0.81 (0.60; 1.09) |  | NA |
|  | No (Reference) |  |  | 1 |  |  |
|  | **Homeownership** |  |  |  |  |  |
|  | Renters/Rent free/Others |  |  | 1.30 (0.98; 1.73) |  | NA |
|  | Owners (Reference) |  |  | 1 |  |  |
|  | **Private medical insurance** |  |  |  |  |  |
|  | No |  |  | 1.06 (0.82; 1.36) |  | NA |
|  | Yes (Reference) |  |  | 1 |  |  |
| **Psychosocial factors** | **PCG mean parental Stressor scale score** |  |  |  | 1.00 (0.97; 1.03) | 1.00 (0.97; 1.03) |
|  | **PCG depression status** |  |  |  |  |  |
|  | Depressed |  |  |  | 1.40 (1.00; 1.95) | NA |
|  | Not depressed (Reference) |  |  |  |  |  |
|  | **Family structure** |  |  |  |  |  |
|  | Single parent family |  |  |  | 1.14 (0.86; 1.49) | NA |
|  | Couple family (Reference) |  |  |  | 1 |  |
|  | **Job stress** |  |  |  |  |  |
|  | Stressed |  |  |  | 0.92 (0.73; 1.16) | 0.91 (0.71; 1.16) |
|  | Not stressed (Reference) |  |  |  | 1 |  |

Table S30 Association between household/family income and young persons’ parent-reported oral health (number of teeth with dental fillings) adjusted for covariates (wave 1), behavioural factors, material factors and psychosocial factors (logistic regression odds ratios (95% CI) for having two teeth with dental fillings). Model 2: Model 1 + behavioural factors; Model 3: Model 1 + material factors; Model 4: Model 1 + Psychosocial factors and Model 5: Model 1 + behavioural factors + material factors + Psychosocial factors. All models were adjusted for the ‘area of residence’, the ‘main language spoken at home’ and ‘PCGs’ country of birth’. OR: Odds Ratio; NA (not applicable) refers to a variable that was excluded from the model using stepwise backward selection.

|  |  | **Model 1** | **Model 2** | **Model 3** | **Model 4** | **Model 5** |
| --- | --- | --- | --- | --- | --- | --- |
|  |  | **OR (95% CI)** | **OR (95% CI)**  **[% change]** | **OR (95% CI)**  **[% change]** | **OR (95% CI)**  **[% change]** | **OR (95% CI)**  **[% change]** |
| **Family income in quintiles** | **Males** |  |  |  |  |  |
|  | Lowest | **1.82 (1.30; 2.55)** | **1.83 (1.29; 2.58)**  [-1.22] | **1.60 (1.07; 2.38)**  [26.83] | **1.58 (1.12; 2.24)**  [29.27] | **1.65 (1.12; 2.42)**  [20.73] |
|  | 2^nd^ | 1.13 (0.80, 1.60) | 1.13 (0.79; 1.62)  [0.00] | 1.00 (0.66; 1.49)  [100.00] | 0.94 (0.65; 1.36)  [100.00] | 0.97 (0.65; 1.45)  [100.00] |
|  | 3^rd^ | 1.34 (0.94; 1.90) | 1.26 (0.88; 1.80)  [23.53] | 1.18 (0.80; 1.74)  [47.06] | 1.17 (0.82; 1.68)  [50.00] | 1.12 (0.77; 1.63)  [64.71] |
|  | 4^th^ | 1.25 (0.89; 1.76) | 1.27 (0.90; 1.79)  [-8.00] | 1.17 (0.82; 1.66)  [32.00] | 1.12 (0.79; 1.59)  [52.00] | 1.13 (0.79; 1.62)  [48.00] |
|  | Highest (Reference) | 1 | 1 | 1 | 1 | 1 |
|  | **Females** |  |  |  |  |  |
|  | Lowest | **1.78 (1.26; 2.52)** | **1.88 (1.32; 2.69)**  **[-12.82]** | **1.87 (1.23; 2.84)**  **[-11.54]** | **1.76 (1.22; 2.53)**  **[2.56]** | **2.16 (1.45; 3.23)**  **[-48.72]** |
|  | 2^nd^ | **1.64 (1.17; 2.31)** | **1.69 (1.19; 2.40)**  **[-7.81]** | **1.73 (1.16; 2.59)**  **[-14.06]** | **1.63 (1.14; 2.33)**  **[1.56]** | **1.96 (1.32; 2.90)**  **[-50.00]** |
|  | 3^rd^ | **1.62 (1.16; 2.29)** | **1.70 (1.20; 2.42)**  **[-12.90]** | **1.79 (1.23; 2.62)**  **[-27.42]** | **1.60 (1.12; 2.27)**  **[3.23]** | **1.97 (1.35; 2.87)**  **[-56.45]** |
|  | 4^th^ | 0.96 (0.66; 1.39) | 0.97 (0.66; 1.41)  [25.00] | 1.04 (0.70; 1.54)  [100.00] | 0.95 (0.65; 1.40)  [-25.00] | 1.07 (0.71; 1.59)  [100.00] |
|  | Highest (Reference) | 1 | 1 | 1 | 1 | 1 |

Table S30 Continued…..

|  | | **OR (95% CI)** | **OR (95% CI)**  **[% change]** | **OR (95% CI)**  **[% change]** | **OR (95% CI)**  **[% change]** | **OR (95% CI)**  **[% change]** |
| --- | --- | --- | --- | --- | --- | --- |
| **Behavioural factors** | |  |  |  |  |  |
| **Daily Toothbrushing**  **Dentist visit behaviour**  **Sugary drinks intake**  **Sugary foods intake** | **Males** |  |  |  |  |  |
|  | Less than once/Rarely/Never |  | 0.89 (0.58; 1.37) |  |  | 0.81 (0.51; 1.27) |
|  | Once |  | **1.36 (1.07; 1.73)** |  |  | **1.31 (1.02; 1.68)** |
|  | Twice/More than twice (Reference) |  | 1 |  |  | 1 |
|  | **Females** |  |  |  |  |  |
|  | Less than once/Rarely/Never |  | 1.29 (0.73; 2.29) |  |  | 1.23 (0.66; 2.28) |
|  | Once |  | 0.89 (0.66; 1.21) |  |  | 0.82 (0.59; 1.14) |
|  | Twice/More than twice (Reference) |  | 1 |  |  | 1 |
|  | **Males** |  |  |  |  |  |
|  | Problem/Never |  | **0.71 (0.51; 0.98)** |  |  | **0.68 (0.48; 0.96)** |
|  | Occasional visit |  | 0.81 (0.61; 1.07) |  |  | 0.80 (0.59; 1.07) |
|  | At least a year (Reference) |  | 1 |  |  | 1 |
|  | **Females** |  |  |  |  |  |
|  | Problem/Never |  | **0.56 (0.40; 0.80)** |  |  | **0.53 (0.36; 0.76)** |
|  | Occasional visit |  | 0.88 (0.67; 1.15) |  |  | 0.84 (0.63; 1.11) |
|  | At least a year (Reference) |  | 1 |  |  | 1 |
|  | **Males** |  |  |  |  |  |
|  | More than once a day |  | 0.93 (0.69; 1.27) |  |  | NA |
|  | Once a day |  | 1.23 (0.97; 1.57) |  |  | NA |
|  | Not at all (Reference) |  | 1 |  |  |  |
|  | **Females** |  |  |  |  |  |
|  | More than once a day |  | 0.96 (0.70; 1.32) |  |  | 0.84 (0.59; 1.19) |
|  | Once a day |  | **1.50 (1.17; 1.92)** |  |  | **1.43 (1.10; 1.86)** |
|  | Not at all (Reference) |  | 1 |  |  | 1 |
|  | **Males** |  |  |  |  |  |
|  | More than once a day |  | **1.42 (1.04; 1.93)** |  |  | NA |
|  | Once a day |  | 0.95 (0.74; 1.23) |  |  | NA |
|  | Not at all (Reference) |  | 1 |  |  |  |

Table S30 Continued…..

| **Behavioural factors** |  | **OR (95% CI)** | **OR (95% CI)**  **[% change]** | **OR (95% CI)**  **[% change]** | **OR (95% CI)**  **[% change]** | **OR (95% CI)**  **[% change]** |
| --- | --- | --- | --- | --- | --- | --- |
| **Sugary foods intake** | **Females** |  |  |  |  |  |
|  | More than once a day |  | 1.01 (0.73; 1.40) |  |  | 0.90 (0.64; 1.28) |
|  | Once a day |  | **1.27 (1.00; 1.63)** |  |  | 1.22 (0.95; 1.58) |
|  | Not at all (Reference) |  | 1 |  |  | 1 |
| **Material factors** | |  |  |  |  |  |
| **Financial difficulties**  **DEIS school**  **Homeownership**  **Private medical insurance** | **Males** |  |  |  |  |  |
|  | With difficulty |  |  | 0.79 (0.62; 1.02) |  | 0.78 (0.61; 1.01) |
|  | No difficulty (Reference) |  |  | 1 |  | 1 |
|  | **Females** |  |  |  |  |  |
|  | With difficulty |  |  | 0.91 (0.71; 1.17) |  | 0.89 (0.69; 1.16) |
|  | No difficulty (Reference) |  |  | 1 |  | 1 |
|  | **Males** |  |  |  |  |  |
|  | Yes |  |  | **1.31 (1.00; 1.73)** |  | 1.25 (0.94; 1.66) |
|  | No (Reference) |  |  | 1 |  | 1 |
|  | **Females** |  |  |  |  |  |
|  | Yes |  |  | 0.95 (0.71; 1.26) |  | NA |
|  | No (Reference) |  |  | 1 |  |  |
|  | **Males** |  |  |  |  |  |
|  | Renters/Rent free/Others |  |  | 1.33 (0.98; 1.81) |  | 1.11 (0.80; 1.53) |
|  | Owners (Reference) |  |  | 1 |  |  |
|  | **Females** |  |  |  |  |  |
|  | Renters/Rent free/Others |  |  | 1.15 (0.87; 1.54) |  | NA |
|  | Owners (Reference) |  |  | 1 |  |  |
|  | **Males** |  |  |  |  |  |
|  | No |  |  | 1.08 (0.83; 1.41) |  | NA |
|  | Yes (Reference) |  |  | 1 |  |  |
|  | **Females** |  |  |  |  |  |
|  | No |  |  | 0.97 (0.75; 1.26) |  | NA |
|  | Yes (Reference) |  |  | 1 |  |  |

Table S30 Continued…..

|  | | **OR (95% CI)** | **OR (95% CI)**  **[% change]** | **OR (95% CI)**  **[% change]** | **OR (95% CI)**  **[% change]** | **OR (95% CI)**  **[% change]** |
| --- | --- | --- | --- | --- | --- | --- |
| **Psychosocial factors** | |  |  |  |  |  |
| **PCG Parental Stressor scale score**  **PCG depression status**  **Family structure**  **Job stress** | **Males** |  |  |  | 1.01 (0.98; 1.04) | NA |
|  | **Females** |  |  |  | 1.03 (1.00; 1.06) | **1.04 (1.01; 1.07)** |
|  | **Males** |  |  |  |  |  |
|  | Depressed |  |  |  | 0.93 (0.66; 1.31) | NA |
|  | Not depressed (Reference) |  |  |  | 1 |  |
|  | **Females** |  |  |  |  |  |
|  | Depressed |  |  |  | 0.90 (0.63; 1.29) | NA |
|  | Not depressed (Reference) |  |  |  | 1 |  |
|  | **Males** |  |  |  |  |  |
|  | Single parent family |  |  |  | **1.83 (1.40; 2.40)** | **1.88 (1.39; 2.52)** |
|  | Couple family (Reference) |  |  |  | 1 | 1 |
|  | **Females** |  |  |  |  |  |
|  | Single parent family |  |  |  | 1.04 (0.79; 1.37) | NA |
|  | Couple family (Reference) |  |  |  | 1 |  |
|  | **Males** |  |  |  |  |  |
|  | Stressed |  |  |  | 0.86 (0.68; 1.08) | NA |
|  | Not stressed (Reference) |  |  |  | 1 |  |
|  | **Females** |  |  |  |  |  |
|  | Stressed |  |  |  | 1.03 (0.81; 1.30) | 0.98 (0.77; 1.25) |
|  | Not stressed (Reference) |  |  |  | 1 | 1 |

Table S31 Association between household/family income and young persons’ parent-reported oral health (number of teeth with dental fillings) adjusted for covariates (wave 1), behavioural factors, material factors and psychosocial factors (logistic regression odds ratios (95% CI) for having three or more teeth with dental fillings). Model 2: Model 1 + behavioural factors; Model 3: Model 1 + material factors; Model 4: Model 1 + Psychosocial factors and Model 5: Model 1 + behavioural factors + material factors + Psychosocial factors. All models were adjusted for the ‘area of residence’, the ‘main language spoken at home’ and ‘PCGs’ country of birth’. OR: Odds Ratio; NA (not applicable) refers to a variable that was excluded from the model using stepwise backward selection.

|  |  | **Model 1** | **Model 2** | **Model 3** | **Model 4** | **Model 5** |
| --- | --- | --- | --- | --- | --- | --- |
|  |  | **OR (95% CI)** | **OR (95% CI)**  **[% change]** | **OR (95% CI)**  **[% change]** | **OR (95% CI)**  **[% change]** | **OR (95% CI)**  **[% change]** |
| **Family income in quintiles** | **Males** |  |  |  |  |  |
|  | Lowest | **1.58 (1.11; 2.25)** | **1.60 (1.11; 2.30)**  **[-3.45]** | 1.26 (0.83; 1.92)  [55.17] | **1.52 (1.05; 2.21)**  **[10.34]** | 1.25 (0.83; 1.88)  [56.90] |
|  | 2^nd^ | 1.04 (0.72; 1.50) | 1.07 (0.73; 1.56)  [-75.00] | 0.88 (0.57; 1.34)  [100.00] | 1.00 (0.68; 1.47)  [100.00] | 0.89 (0.58; 1.35)  [100.00] |
|  | 3^rd^ | 1.31 (0.91; 1.87) | 1.32 (0.91; 1.90)  [-3.23] | 1.22 (0.82; 1.82)  [29.03] | 1.29 (0.88; 1.88)  [6.45] | 1.18 (0.80; 1.75)  [41.94] |
|  | 4^th^ | 1.37 (0.97; 1.94) | 1.28 (0.89; 1.82)  [24.32] | 1.32 (0.91; 1.90)  [13.51] | 1.42 (0.99; 2.03)  [-13.51] | 1.16 (0.80; 1.69)  [56.76] |
|  | Highest (Reference) | 1 | 1 | 1 | 1 | 1 |
|  | **Females** |  |  |  |  |  |
|  | Lowest | **1.89 (1.34; 2.66)** | **1.70 (1.20; 2.43)**  **[21.35]** | 1.40 (0.93; 2.13)  [55.06] | **1.66 (1.15; 2.39)**  **[25.84]** | 1.50 (1.00; 2.23)  [43.82] |
|  | 2^nd^ | 0.97 (0.68; 1.41) | 0.96 (0.66; 1.40)  [-33.33] | 0.76 (0.49; 1.17)  [100.00] | 0.93 (0.63; 1.37)  [100.00] | 0.84 (0.55; 1.29)  [100.00] |
|  | 3^rd^ | **1.42 (1.01; 2.01)** | **1.46 (1.02; 2.08)**  **[-9.52]** | 1.24 (0.85; 1.82)  [42.86] | 1.42 (0.99; 2.03)  [0.00] | **1.50 (1.02; 2.20)**  **[-19.05]** |
|  | 4^th^ | 1.09 (0.76; 1.56) | 1.04 (0.72; 1.51)  [55.56] | 0.94 (0.64; 1.38)  [100.00] | 1.00 (0.69; 1.47)  [100.00] | 0.96 (0.64; 1.42)  [100.00] |
|  | Highest (Reference) | 1 | 1 | 1 | 1 | 1 |

Table S31 Continued…..

| **Behavioural factors** |  | **OR (95% CI)** | **OR (95% CI)**  **[% change]** | **OR (95% CI)**  **[% change]** | **OR (95% CI)**  **[% change]** | **OR (95% CI)**  **[% change]** |
| --- | --- | --- | --- | --- | --- | --- |
| **Daily Toothbrushing frequency**  **Dentist visit behaviour**  **Sugary drinks intake**  **Sugary foods intake** | **Males** |  |  |  |  |  |
|  | Less than once/Rarely/Never |  | 1.29 (0.85; 1.94) |  |  | 1.33 (0.87; 2.02) |
|  | Once |  | **1.34 (1.04; 1.72)** |  |  | **1.31 (1.01; 1.70)** |
|  | Twice/More than twice (Reference) |  |  |  |  |  |
|  | **Females** |  |  |  |  |  |
|  | Less than once/Rarely/Never |  | **2.55 (1.54; 4.21)** |  |  | **2.23 (1.28; 3.87)** |
|  | Once |  | **1.45 (1.09; 1.92)** |  |  | **1.41 (1.04; 1.91)** |
|  | Twice/More than twice (Reference) |  |  |  |  |  |
|  | **Males** |  |  |  |  |  |
|  | Problem/Never |  | **0.64 (0.45; 0.90)** |  |  | **0.65 (0.45; 0.92)** |
|  | Occasional visit |  | 0.79 (0.59; 1.06) |  |  | 0.81 (0.60; 1.09) |
|  | At least a year (Reference) |  |  |  |  |  |
|  | **Females** |  |  |  |  |  |
|  | Problem/Never |  | **0.63 (0.45; 0.90)** |  |  | **0.64 (0.44; 0.93)** |
|  | Occasional visit |  | 0.83 (0.62; 1.09) |  |  | 0.75 (0.55; 1.01) |
|  | At least a year (Reference) |  |  |  |  |  |
|  | **Males** |  |  |  |  |  |
|  | More than once a day |  | 1.07 (0.78; 1.47) |  |  | NA |
|  | Once a day |  | **1.30 (1.01; 1.67)** |  |  | NA |
|  | Not at all (Reference) |  |  |  |  |  |
|  | **Females** |  |  |  |  |  |
|  | More than once a day |  | 1.11 (0.82; 1.51) |  |  | 1.05 (0.76; 1.47) |
|  | Once a day |  | 1.06 (0.81; 1.39) |  |  | 1.00 (0.75; 1.33) |
|  | Not at all (Reference) |  |  |  |  |  |
|  | **Males** |  |  |  |  |  |
|  | More than once a day |  | 1.07 (0.76; 1.49) |  |  | NA |
|  | Once a day |  | 0.95 (0.73; 1.23) |  |  | NA |
|  | Not at all (Reference) |  |  |  |  |  |

Table S31 Continued…..

| **Behavioural factors** |  | **OR (95% CI)** | **OR (95% CI)**  **[% change]** | **OR (95% CI)**  **[% change]** | **OR (95% CI)**  **[% change]** | **OR (95% CI)**  **[% change]** |
| --- | --- | --- | --- | --- | --- | --- |
| **Sugary foods intake** | **Females** |  |  |  |  |  |
|  | More than once a day |  | **1.55 (1.14; 2.12)** |  |  | **1.47 (1.05; 2.05)** |
|  | Once a day |  | 1.22 (0.94; 1.58) |  |  | 1.23 (0.93; 1.61) |
|  | Not at all (Reference) |  |  |  |  |  |
| **Material factors**  **Financial difficulties**  **DEIS school**  **Homeownership**  **Private medical insurance** | **Males** |  |  |  |  |  |
|  | With difficulty |  |  | 1.22 (0.94; 1.58) |  | 1.21 (0.93; 1.58) |
|  | No difficulty (Reference) |  |  |  |  |  |
|  | **Females** |  |  |  |  |  |
|  | With difficulty |  |  | **1.39 (1.07; 1.81)** |  | **1.36 (1.03; 1.79)** |
|  | No difficulty (Reference) |  |  |  |  |  |
|  | **Males** |  |  |  |  |  |
|  | Yes |  |  | 1.22 (0.91; 1.62) |  | 1.22 (0.90; 1.64) |
|  | No (Reference) |  |  |  |  |  |
|  | **Females** |  |  |  |  |  |
|  | Yes |  |  | 0.87 (0.64; 1.17) |  | NA |
|  | No (Reference) |  |  |  |  |  |
|  | **Males** |  |  |  |  |  |
|  | Renters/Rent free/Others |  |  | 1.30 (0.94; 1.80) |  | 1.01 (0.71; 1.43) |
|  | Owners (Reference) |  |  |  |  |  |
|  | **Females** |  |  |  |  |  |
|  | Renters/Rent free/Others |  |  | 1.25 (0.93; 1.68) |  | NA |
|  | Owners (Reference) |  |  |  |  |  |
|  | **Males** |  |  |  |  |  |
|  | No |  |  | 1.04 (0.79; 1.36) |  | NA |
|  | Yes (Reference) |  |  |  |  |  |
|  | **Females** |  |  |  |  |  |
|  | No |  |  | 1.08 (0.83; 1.42) |  | NA |
|  | Yes (Reference) |  |  |  |  |  |

Table S31 Continued…..

| **Psychosocial factors** |  | **OR (95% CI)** | **OR (95% CI)**  **[% change]** | **OR (95% CI)**  **[% change]** | **OR (95% CI)**  **[% change]** | **OR (95% CI)**  **[% change]** |
| --- | --- | --- | --- | --- | --- | --- |
| **PCG Parental Stressor scale score**  **PCG depression status**  **Family structure**  **Job stress** | **Males** |  |  |  | 1.04 (1.02; 1.07) | NA |
|  | **Females** |  |  |  | 1.00 (0.97; 1.03) | 1.00 (0.96; 1.03) |
|  | **Males** |  |  |  |  |  |
|  | Depressed |  |  |  | 1.07 (0.76; 1.50) | NA |
|  | Not depressed (Reference) |  |  |  |  |  |
|  | **Females** |  |  |  |  |  |
|  | Depressed |  |  |  | 1.38 (0.97; 1.95) | NA |
|  | Not depressed (Reference) |  |  |  |  |  |
|  | **Males** |  |  |  |  |  |
|  | Single parent family |  |  |  | **1.47 (1.10; 1.97)** | 1.31 (0.94; 1.81) |
|  | Couple family (Reference) |  |  |  |  |  |
|  | **Females** |  |  |  |  |  |
|  | Single parent family |  |  |  | 1.06 (0.79; 1.42) | NA |
|  | Couple family (Reference) |  |  |  |  |  |
|  | **Males** |  |  |  |  |  |
|  | Stressed |  |  |  | **0.75 (0.60; 0.95)** | NA |
|  | Not stressed (Reference) |  |  |  |  |  |
|  | **Females** |  |  |  |  |  |
|  | Stressed |  |  |  | 1.03 (0.81; 1.32) | 1.00 (0.78; 1.29) |
|  | Not stressed (Reference) |  |  |  |  |  |

Table S32 Association between family occupational class and young females’ parent-reported oral health (number of teeth with dental fillings) adjusted for covariates (wave 1), behavioural factors, material factors and psychosocial factors (logistic regression odds ratios (95% CI) for having two teeth with dental fillings). Model 2: Model 1 + behavioural factors; Model 3: Model 1 + material factors; Model 4: Model 1 + Psychosocial factors and Model 5: Model 1 + behavioural factors + material factors + Psychosocial factors. All models were adjusted for the ‘area of residence’, the ‘main language spoken at home’ and ‘PCGs’ country of birth’. OR: Odds Ratio; NA (not applicable) refers to a variable that was excluded from the model using stepwise backward selection.

|  |  | **Model 1** | **Model 2** | **Model 3** | **Model 4** | **Model 5** |
| --- | --- | --- | --- | --- | --- | --- |
|  |  | **OR (95% CI)** | **OR (95% CI)**  **[% change]** | **OR (95% CI)**  **[% change]** | **OR (95% CI)**  **[% change]** | **OR (95% CI)**  **[% change]** |
| **Family occupational class** | Semi-skilled/ Unskilled manual | **1.74 (1.29; 2.36)** | **1.77 (1.30; 2.41)**  **[-4.05]** | **1.51 (1.07; 2.12)**  **[31.08]** | **1.65 (1.21; 2.26)**  **[12.16]** | **1.56 (1.12; 2.18)**  **[24.32]** |
|  | Other non-manual/ Skilled manual | 1.19 (0.94; 1.50) | 1.17 (0.92; 1.49)  [10.53] | 1.14 (0.88; 1.49)  [26.32] | 1.17 (0.91; 1.49)  [10.53] | 1.17 (0.90; 1.52)  [10.53] |
|  | Professional managers (Reference) | 1 | 1 | 1 | 1 | 1 |
| **Behaviour factors** | **Toothbrushing frequency** |  |  |  |  |  |
|  | Less than once/Rarely/Never |  | 1.25 (0.64; 2.42) |  |  | 1.02 (0.51; 2.07) |
|  | Once |  | 0.92 (0.68; 1.25) |  |  | 0.87 (0.62; 1.20) |
|  | Twice/More than twice (Reference) |  | 1 |  |  | 1 |
|  | **Dentist visit behaviour** |  |  |  |  |  |
|  | Problem/Never |  | **0.66 (0.46; 0.94)** |  |  | **0.57 (0.39; 0.84)** |
|  | Occasional visit |  | 0.85 (0.65; 1.12) |  |  | 0.80 (0.60; 1.08) |
|  | At least a year (Reference) |  | 1 |  |  | 1 |
|  | **Sugary drinks intake** |  |  |  |  |  |
|  | More than once a day |  | 1.24 (0.89; 1.72) |  |  | 1.07 (0.75; 1.53) |
|  | Once a day |  | **1.60 (1.25; 2.06)** |  |  | **1.47 (1.13; 1.92)** |
|  | Not at all (Reference) |  | 1 |  |  | 1 |
|  | **Sugary foods intake** |  |  |  |  |  |
|  | More than once a day |  | 0.93 (0.67; 1.30) |  |  | 0.80 (0.56; 1.15) |
|  | Once a day |  | 1.18 (0.92; 1.51) |  |  | 1.08 (0.83; 1.40) |
|  | Not at all (Reference) |  | 1 |  |  | 1 |

Table S32 Continued…..

|  |  | **OR (95% CI)** | **OR (95% CI)**  **[% change]** | **OR (95% CI)**  **[% change]** | **OR (95% CI)**  **[% change]** | **OR (95% CI)**  **[% change]** |
| --- | --- | --- | --- | --- | --- | --- |
| **Material factors** | **Financial difficulties** |  |  |  |  |  |
|  | With difficulty |  |  | 1.20 (0.94; 1.52) |  | 1.18 (0.93; 1.51) |
|  | No difficulty (Reference) |  |  | 1 |  | 1 |
|  | **DEIS school** |  |  |  |  |  |
|  | Yes |  |  | 1.06 (0.79; 1.42) |  | NA |
|  | No (Reference) |  |  | 1 |  |  |
|  | **Homeownership** |  |  |  |  |  |
|  | Renters/Rent free/Others |  |  | **1.61 (1.18; 2.19)** |  | **1.59 (1.16; 2.16)** |
|  | Owners (Reference) |  |  |  |  |  |
|  | **Private medical insurance** |  |  |  |  |  |
|  | No |  |  | 0.95 (0.73; 1.23) |  | NA |
|  | Yes (Reference) |  |  | 1 |  |  |
| **Psychosocial factors** | **PCG mean parental stressor scale score** |  |  |  | 1.02 (1.00; 1.05) | 1.03 (1.00; 1.06) |
|  | **PCG depression status** |  |  |  | 1 | 1 |
|  | Depressed |  |  |  | 1.04 (0.71; 1.52) | NA |
|  | Not depressed (Reference) |  |  |  | 1 |  |
|  | **Family structure** |  |  |  |  |  |
|  | Single parent family |  |  |  | **1.45 (1.07; 1.98)** | NA |
|  | Couple family (Reference) |  |  |  | 1 |  |
|  | **PCG job stress** |  |  |  |  |  |
|  | Stressed |  |  |  | 1.01 (0.79; 1.28) | NA |
|  | Not stressed (Reference) |  |  |  | 1 |  |

Table S33 Association between family occupational class and young females’ parent-reported oral health (number of teeth with dental fillings) adjusted for covariates (wave 1), behavioural factors, material factors and psychosocial factors (logistic regression odds ratios (95% CI) for having three teeth with dental fillings). Model 2: Model 1 + behavioural factors; Model 3: Model 1 + material factors; Model 4: Model 1 + Psychosocial factors and Model 5: Model 1 + behavioural factors + material factors + Psychosocial factors. All models were adjusted for the ‘area of residence’, the ‘main language spoken at home’ and ‘PCGs’ country of birth’. OR: Odds Ratio; NA (not applicable) refers to a variable that was excluded from the model using stepwise backward selection.

|  |  | **Model 1** | **Model 2** | **Model 3** | **Model 4** | **Model 5** |
| --- | --- | --- | --- | --- | --- | --- |
|  |  | **OR (95% CI)** | **OR (95% CI)**  **[% change]** | **OR (95% CI)**  **[% change]** | **OR (95% CI)**  **[% change]** | **OR (95% CI)**  **[% change]** |
| **Family occupational class** | Semi-skilled/ Unskilled manual | **1.70 (1.24; 2.33)** | **1.61 (1.16; 2.24)**  **[**12.86] | 1.35 (0.94; 1.95)  [50.00] | **1.56 (1.11; 2.19)**  **[20.00]** | 1.29 (0.90; 1.86)  [58.57] |
|  | Other non-manual/ Skilled manual | **1.39 (1.10; 1.77)** | **1.31 (1.02; 1.67)**  [20.51] | 1.29 (0.99; 1.70)  [25.64] | **1.32 (1.03; 1.70)**  **[17.95]** | 1.19 (0.91; 1.55)  [51.28] |
|  | Professional managers (Reference) | 1 | 1 | 1 | 1 | 1 |
| **Behavioural factors** | **Toothbrushing frequency** |  |  |  |  |  |
|  | Less than once/Rarely/Never |  | **2.83 (1.61; 4.97)** |  |  | **2.43 (1.33; 4.43)** |
|  | Once |  | **1.39 (1.04; 1.86)** |  |  | **1.39 (1.02; 1.90)** |
|  | Twice/More than twice (Reference) |  |  |  |  |  |
|  | **Dentist visit behaviour** |  |  |  |  |  |
|  | Problem/Never |  | **0.67 (0.47; 0.97)** |  |  | **0.67 (0.45; 0.99)** |
|  | Occasional visit |  | 0.85 (0.64; 1.13) |  |  | 0.78 (0.58; 1.06) |
|  | At least a year (Reference) |  |  |  |  |  |
|  | **Sugary drinks intake** |  |  |  |  |  |
|  | More than once a day |  | 1.21 (0.88; 1.67) |  |  | 1.07 (0.75; 1.52) |
|  | Once a day |  | 1.08 (0.82; 1.42) |  |  | 0.97 (0.72; 1.30) |
|  | Not at all (Reference) |  |  |  |  |  |
|  | **Sugary foods intake** |  |  |  |  |  |
|  | More than once a day |  | **1.55 (1.12; 2.15)** |  |  | **1.48 (1.04; 2.11)** |
|  | Once a day |  | 1.25 (0.96; 1.62) |  |  | 1.23 (0.93; 1.63) |
|  | Not at all (Reference) |  |  |  |  |  |

Table S33 Continued….

|  |  | **OR (95% CI)** | **OR (95% CI)**  **[% change]** | **OR (95% CI)**  **[% change]** | **OR (95% CI)**  **[% change]** | **OR (95% CI)**  **[% change]** |
| --- | --- | --- | --- | --- | --- | --- |
| **Material factors** | **Financial difficulties** |  |  |  |  |  |
|  | With difficulty |  |  | **1.58 (1.22; 2.03)** |  | **1.54 (1.19; 1.99)** |
|  | No difficulty (Reference) |  |  |  |  |  |
|  | **DEIS school** |  |  |  |  |  |
|  | Yes |  |  | 0.84 (0.61; 1.15) |  | NA |
|  | No (Reference) |  |  |  |  |  |
|  | **Homeownership** |  |  |  |  |  |
|  | Renters/Rent free/Others |  |  | 1.21 (0.87; 1.69) |  | 1.23 (0.88; 1.73) |
|  | Owners (Reference) |  |  |  |  |  |
|  | **Private medical insurance** |  |  |  |  |  |
|  | No |  |  | 0.99 (0.76; 1.29) |  | NA |
|  | Yes (Reference) |  |  |  |  |  |
| **Psychosocial factors** | **PCG mean parental stressor scale score** |  |  |  | 0.99 (0.96; 1.02) | 0.98 (0.95; 1.01) |
|  | **PCG depression status** |  |  |  |  |  |
|  | Depressed |  |  |  | **1.47 (1.01; 2.15)** | NA |
|  | Not depressed (Reference) |  |  |  |  |  |
|  | **Family structure** |  |  |  |  |  |
|  | Single parent family |  |  |  | 0.87 (0.60; 1.26) | NA |
|  | Couple family (Reference) |  |  |  |  |  |
|  | **PCG job stress** |  |  |  |  |  |
|  | Stressed |  |  |  | 1.06 (0.82; 1.37) | NA |
|  | Not stressed (Reference) |  |  |  |  |  |

Table S34 Association between family medical card status and young persons’ parent-reported oral health (number of teeth with dental fillings) adjusted for covariates (wave 1), behavioural factors, material factors and psychosocial factors (logistic regression odds ratios (95% CI) for having two teeth with dental fillings). Model 2: Model 1 + behavioural factors; Model 3: Model 1 + material factors; Model 4: Model 1 + Psychosocial factors and Model 5: Model 1 + behavioural factors + material factors + Psychosocial factors. All models were adjusted for the ‘area of residence’, the ‘main language spoken at home’ and ‘PCGs’ country of birth’. OR: Odds Ratio; NA (not applicable) refers to a variable that was excluded from the model using stepwise backward selection.

|  |  | **Model 1** | **Model 2** | **Model 3** | **Model 4** | **Model 5** |
| --- | --- | --- | --- | --- | --- | --- |
|  |  | **OR (95% CI)** | **OR (95% CI)**  **[% change]** | **OR (95% CI)**  **[% change]** | **OR (95% CI)**  **[% change]** | **OR (95% CI)**  **[% change]** |
| **Study participants’ medical card status** | **Males** |  |  |  |  |  |
|  | Yes, full card | **1.65 (1.34; 2.05)** | **1.66 (1.34; 2.07)**  **[-1.54]** | **1.72 (1.29; 2.30)**  **[-10.77]** | **1.41 (1.11; 1.80)**  **[36.92]** | **1.47 (1.13; 1.93)**  **[27.69]** |
|  | Yes, doctor only card | 0.60 (0.26; 1.40) | 0.61 (0.26; 1.42)  [2.50] | 0.61 (0.26; 1.43)  [2.50] | 0.54 (0.23; 1.28)  [-15.00] | 0.60 (0.25; 1.40)  [0.00] |
|  | Not covered (Reference) | 1 | 1 | 1 | 1 | 1 |
|  | **Females** |  |  |  |  |  |
|  | Yes, full card | **1.41 (1.14; 1.74)** | **1.49 (1.20; 1.86)**  **[-19.51]** | 1.26 (0.95; 1.67)  [36.59] | **1.41 (1.11; 1.78)**  **[0.00]** | **1.57 (1.23; 1.99)**  **[-39.02]** |
|  | Yes, doctor only card | 1.43 (0.75; 2.73) | 1.55 (0.80; 2.98)  [-27.91] | 1.26 (0.64; 2.51)  [39.53] | 1.37 (0.70; 2.68)  [13.95] | 1.57 (0.79; 3.11)  [-32.56] |
|  | Not covered (Reference) | 1 | 1 | 1 | 1 | 1 |
| **Behavioural factors**  **Toothbrushing frequency** | **Males** |  |  |  |  |  |
|  | Less than once/Rarely/Never |  | 0.88 (0.58; 1.33) |  |  | 0.75 (0.48; 1.18) |
|  | Once |  | **1.30 (1.03; 1.65)** |  |  | 1.22 (0.95; 1.56) |
|  | Twice/More than twice (Reference) |  |  |  |  |  |
|  | **Females** |  |  |  |  |  |
|  | Less than once/Rarely/Never |  | 1.21 (0.68; 2.13) |  |  | 1.26 (0.68; 2.32) |
|  | Once |  | 0.96 (0.71; 1.28) |  |  | 0.89 (0.65; 1.21) |
|  | Twice/More than twice (Reference) |  |  |  |  |  |

Table S34 Continued…..

| **Behavioural factors** |  | **OR (95% CI)** | **OR (95% CI)**  **[% change]** | **OR (95% CI)**  **[% change]** | **OR (95% CI)**  **[% change]** | **OR (95% CI)**  **[% change]** |
| --- | --- | --- | --- | --- | --- | --- |
| **Dentist visit behaviour**  **Sugary drinks intake**  **Sugary foods intake** | **Males** |  |  |  |  |  |
|  | Problem/Never |  | **0.65 (0.47; 0.90)** |  |  | **0.65 (0.46; 0.92)** |
|  | Occasional visit |  | 0.83 (0.63; 1.09) |  |  | 0.81 (0.61; 1.08) |
|  | At least a year (Reference) |  |  |  |  |  |
|  | **Females** |  |  |  |  |  |
|  | Problem/Never |  | **0.50 (0.36; 0.71)** |  |  | **0.48 (0.33; 0.68)** |
|  | Occasional visit |  | 0.89 (0.69; 1.16) |  |  | 0.84 (0.64; 1.11) |
|  | At least a year (Reference) |  |  |  |  |  |
|  | **Males** |  |  |  |  |  |
|  | More than once a day |  | 0.93 (0.69; 1.25) |  |  | 0.94 (0.70; 1.28) |
|  | Once a day |  | 1.21 (0.96; 1.54) |  |  | 1.19 (0.93; 1.53) |
|  | Not at all (Reference) |  |  |  |  |  |
|  | **Females** |  |  |  |  |  |
|  | More than once a day |  | 1.06 (0.74; 1.44) |  |  | NA |
|  | Once a day |  | **1.52 (1.19; 1.93)** |  |  | NA |
|  | Not at all (Reference) |  |  |  |  |  |
|  | **Males** |  |  |  |  |  |
|  | More than once a day |  | **1.57 (1.16; 2.11)** |  |  | **1.59 (1.16; 2.17)** |
|  | Once a day |  | 0.93 (0.73; 1.20) |  |  | 0.96 (0.74; 1.25) |
|  | Not at all (Reference) |  |  |  |  |  |
|  | **Females** |  |  |  |  |  |
|  | More than once a day |  | 0.99 (0.72; 1.35) |  |  | 0.88 (0.64; 1.22) |
|  | Once a day |  | **1.28 (1.02; 1.62)** |  |  | 1.26 (0.98; 1.60) |
|  | Not at all (Reference) |  |  |  |  |  |
| **Material factors**  **Financial difficulties** | **Males** |  |  |  |  |  |
|  | With difficulty |  |  | **0.76 (0.60; 0.96)** |  | 0.79 (0.63; 1.00) |
|  | No difficulty (Reference) |  |  |  |  |  |
|  | **Females** |  |  |  |  |  |
|  | With difficulty |  |  | 1.03 (0.82; 1.31) |  | 1.03 (0.81; 1.30) |
|  | No difficulty (Reference) |  |  |  |  |  |

Table S34 Continued….

| **Material factors** |  | **OR (95% CI)** | **OR (95% CI)**  **[% change]** | **OR (95% CI)**  **[% change]** | **OR (95% CI)**  **[% change]** | **OR (95% CI)**  **[% change]** |
| --- | --- | --- | --- | --- | --- | --- |
| **DEIS school**  **Homeownership**  **Private medical insurance** | **Males** |  |  |  |  |  |
|  | Yes |  |  | 1.26 (0.96; 1.66) |  | NA |
|  | No (Reference) |  |  |  |  |  |
|  | **Females** |  |  |  |  |  |
|  | Yes |  |  | 1.02 (0.77; 1.34) |  | NA |
|  | No (Reference) |  |  |  |  |  |
|  | **Males** |  |  |  |  |  |
|  | Renters/Rent free/Others |  |  | 1.21 (0.88; 1.65) |  | 1.06 (0.77; 1.46) |
|  | Owners (Reference) |  |  |  |  |  |
|  | **Females** |  |  |  |  |  |
|  | Renters/Rent free/Others |  |  | 1.22 (0.92; 1.64) |  | NA |
|  | Owners (Reference) |  |  |  |  |  |
|  | **Males** |  |  |  |  |  |
|  | No |  |  | 0.87 (0.66; 1.14) |  | NA |
|  | Yes (Reference) |  |  |  |  |  |
|  | **Females** |  |  |  |  |  |
|  | No |  |  | 1.05 (0.81; 1.36) |  | NA |
|  | Yes (Reference) |  |  |  |  |  |
| **Psychosocial factors**  **PCG Parental Stressor scale score**  **PCG depression status** | **Males** |  |  |  | 1.00 (0.98; 1.03) | NA |
|  | **Females** |  |  |  | **1.03 (1.01; 1.06)** | **1.04 (1.01; 1.07)** |
|  | **Males** |  |  |  |  |  |
|  | Depressed |  |  |  | 0.89 (0.64; 1.25) | NA |
|  | Not depressed (Reference) |  |  |  |  |  |
|  | **Females** |  |  |  |  |  |
|  | Depressed |  |  |  | 0.83 (0.58; 1.18) | NA |
|  | Not depressed (Reference) |  |  |  |  |  |

Table S34 Continued…..

| **Psychosocial factors** |  | **OR (95% CI)** | **OR (95% CI)**  **[% change]** | **OR (95% CI)**  **[% change]** | **OR (95% CI)**  **[% change]** | **OR (95% CI)**  **[% change]** |
| --- | --- | --- | --- | --- | --- | --- |
| **Family structure**  **Job stress** | **Males** |  |  |  |  |  |
|  | Single parent family |  |  |  | **1.65 (1.24; 2.18)** | **1.73 (1.28; 2.34)** |
|  | Couple family (Reference) |  |  |  |  |  |
|  | **Females** |  |  |  |  |  |
|  | Single parent family |  |  |  | 1.04 (0.78; 1.37) | NA |
|  | Couple family (Reference) |  |  |  |  |  |
|  | **Males** |  |  |  |  |  |
|  | Stressed |  |  |  | 0.82 (0.66; 1.03) | NA |
|  | Not stressed (Reference) |  |  |  |  |  |
|  | **Females** |  |  |  |  |  |
|  | Stressed |  |  |  | 0.96 (0.77; 1.21) | 0.93 (0.74; 1.18) |
|  | Not stressed (Reference) |  |  |  |  |  |

Table S35 Association between family medical card status and young persons’ parent-reported oral health (number of teeth with dental fillings) adjusted for covariates (wave 1), behavioural factors, material factors and psychosocial factors (logistic regression odds ratios (95% CI) for having three or more teeth with dental fillings). Model 2: Model 1 + behavioural factors; Model 3: Model 1 + material factors; Model 4: Model 1 + Psychosocial factors and Model 5: Model 1 + behavioural factors + material factors + Psychosocial factors. All models were adjusted for the ‘area of residence’, the ‘main language spoken at home’ and ‘PCGs’ country of birth’. OR: Odds Ratio; NA (not applicable) refers to a variable that was excluded from the model using stepwise backward selection.

|  |  | **Model 1** | **Model 2** | **Model 3** | **Model 4** | **Model 5** |
| --- | --- | --- | --- | --- | --- | --- |
|  |  | **OR (95% CI)** | **OR (95% CI)**  **[% change]** | **OR (95% CI)**  **[% change]** | **OR (95% CI)**  **[% change]** | **OR (95% CI)**  **[% change]** |
| **Study participants’ medical card status** | **Males** |  |  |  |  |  |
|  | Yes, full card | **1.44 (1.16; 1.79)** | **1.43 (1.14; 1.80)**  **[2.27]** | **1.39 (1.04; 1.85)**  **[11.36]** | **1.34 (1.05; 1.71)**  **[22.73]** | 1.30 (1.00; 1.70)  [31.82] |
|  | Yes, doctor only card | 0.48 (0.19; 1.21) | 0.51 (0.20; 1.28)  [5.77] | 0.44 (0.17; 1.10)  [-7.69] | 0.46 (0.18; 1.16)  [-3.85] | 0.47 (0.18; 1.17)  [-1.92] |
|  | Not covered (Reference) | 1 | 1 | 1 | 1 | 1 |
|  | **Females** |  |  |  |  |  |
|  | Yes, full card | **1.62 (1.31; 2.00)** | **1.59 (1.27; 1.97)**  **[4.84]** | 1.31 (0.98; 1.75)  [50.00] | **1.47 (1.16; 1.87)**  **[24.19]** | **1.41 (1.10; 1.81)**  **[33.87]** |
|  | Yes, doctor only card | 1.26 (0.62; 2.54) | 1.35 (0.66; 0.75)  [-34.62] | 1.12 (0.53; 2.33)  [53.85] | 0.87 (0.38; 1.99)  [100.00] | 0.84 (0.36; 1.98)  [100.00] |
|  | Not covered (Reference) | 1 | 1 | 1 | 1 | 1 |
| **Behavioural factors**  **Daily Toothbrushing** | **Males** |  |  |  |  |  |
|  | Less than once/Rarely/Never |  | 1.22 (0.82; 1.82) |  |  | 1.25 (0.83; 1.89) |
|  | Once |  | **1.39 (1.10; 1.77)** |  |  | **1.31 (1.02; 1.68)** |
|  | Twice/More than twice (Reference) |  |  |  |  |  |
|  | **Females** |  |  |  |  |  |
|  | Less than once/Rarely/Never |  | **2.29 (1.40; 3.76)** |  |  | **2.20 (1.27; 3.80)** |
|  | Once |  | **1.47 (1.13; 1.95)** |  |  | **1.42 (1.06; 1.91)** |
|  | Twice/More than twice (Reference) |  |  |  |  |  |

Table S35 Continued…..

| **Behaviour factors** |  | **OR (95% CI)** | **OR (95% CI)**  **[% change]** | **OR (95% CI)**  **[% change]** | **OR (95% CI)**  **[% change]** | **OR (95% CI)**  **[% change]** |
| --- | --- | --- | --- | --- | --- | --- |
| **Dentist visit behaviour**  **Sugary drinks intake**  **Sugary foods intake** | **Males** |  |  |  |  |  |
|  | Problem/Never |  | **0.59 (0.42; 0.83)** |  |  | **0.59 (0.42; 0.84)** |
|  | Occasional visit |  | 0.82 (0.62; 1.08) |  |  | 0.78 (0.58; 1.04) |
|  | At least a year (Reference) |  |  |  |  |  |
|  | **Females** |  |  |  |  |  |
|  | Problem/Never |  | **0.53 (0.37; 0.74)** |  |  | **0.55 (0.38; 0.78)** |
|  | Occasional visit |  | 0.82 (0.63; 1.08) |  |  | 0.75 (0.56; 1.00) |
|  | At least a year (Reference) |  |  |  |  |  |
|  | **Males** |  |  |  |  |  |
|  | More than once a day |  | 0.96 (0.70; 1.30) |  |  | 0.81 (0.58; 1.12) |
|  | Once a day |  | **1.33 (1.05; 1.69)** |  |  | **1.36 (1.06; 1.74)** |
|  | Not at all (Reference) |  |  |  |  |  |
|  | **Females** |  |  |  |  |  |
|  | More than once a day |  | 1.02 (0.75; 1.37) |  |  | NA |
|  | Once a day |  | 1.10 (0.85; 1.42) |  |  | NA |
|  | Not at all (Reference) |  |  |  |  |  |
|  | **Males** |  |  |  |  |  |
|  | More than once a day |  | 1.13 (0.82; 1.55) |  |  | 1.11 (0.80; 1.54) |
|  | Once a day |  | 0.87 (0.68; 1.12) |  |  | 0.85 (0.66; 1.10) |
|  | Not at all (Reference) |  |  |  |  |  |
|  | **Females** |  |  |  |  |  |
|  | More than once a day |  | **1.59 (1.18; 2.14)** |  |  | **1.56 (1.14; 2.14)** |
|  | Once a day |  | 1.22 (0.95; 1.57) |  |  | 1.27 (0.97; 1.65) |
|  | Not at all (Reference) |  |  |  |  |  |
| **Material factors**  **Financial difficulties** | **Males** |  |  |  |  |  |
|  | With difficulty |  |  | 1.22 (0.96; 1.55) |  | 1.24 (0.98; 1.58) |
|  | No difficulty (Reference) |  |  |  |  |  |
|  | **Females** |  |  |  |  |  |
|  | With difficulty |  |  | **1.41 (1.10; 1.80)** |  | **1.36 (1.06; 1.75)** |
|  | No difficulty (Reference) |  |  |  |  |  |

Table S35 Continued…..

| **Material factors** |  | **OR (95% CI)** | **OR (95% CI)**  **[% change]** | **OR (95% CI)**  **[% change]** | **OR (95% CI)**  **[% change]** | **OR (95% CI)**  **[% change]** |
| --- | --- | --- | --- | --- | --- | --- |
| **DEIS school**  **Homeownership**  **Private medical insurance** | **Males** |  |  |  |  |  |
|  | Yes |  |  | 1.18 (0.90; 1.56) |  | NA |
|  | No (Reference) |  |  |  |  |  |
|  | **Females** |  |  |  |  |  |
|  | Yes |  |  | 0.82 (0.61; 1.10) |  | NA |
|  | No (Reference) |  |  |  |  |  |
|  | **Males** |  |  |  |  |  |
|  | Renters/Rent free/Others |  |  | 1.20 (0.87; 1.65) |  | 0.98 (0.70; 1.37) |
|  | Owners (Reference) |  |  |  |  |  |
|  | **Females** |  |  |  |  |  |
|  | Renters/Rent free/Others |  |  | 1.22 (0.91; 1.64) |  | NA |
|  | Owners (Reference) |  |  |  |  |  |
|  | **Males** |  |  |  |  |  |
|  | No |  |  | 0.84 (0.64; 1.10) |  | NA |
|  | Yes (Reference) |  |  |  |  |  |
|  | **Females** |  |  |  |  |  |
|  | No |  |  | 0.99 (0.76; 1.30) |  | NA |
|  | Yes (Reference) |  |  |  |  |  |
| **Psychosocial factors**  **PCG Parental Stressor scale score**  **PCG depression status** | **Males** |  |  |  | **1.04 (1.02; 1.07)** | NA |
|  | **Females** |  |  |  | 1.00 (0.97; 1.03) | 0.99 (0.96; 1.02) |
|  | **Males** |  |  |  |  |  |
|  | Depressed |  |  |  | 1.01 (0.72; 1.40) | NA |
|  | Not depressed (Reference) |  |  |  |  |  |
|  | **Females** |  |  |  |  |  |
|  | Depressed |  |  |  | 1.33 (0.95; 1.85) | NA |
|  | Not depressed (Reference) |  |  |  |  |  |

Table S35 Continued…..

| **Psychosocial factors** |  | **OR (95% CI)** | **OR (95% CI)**  **[% change]** | **OR (95% CI)**  **[% change]** | **OR (95% CI)**  **[% change]** | **OR (95% CI)**  **[% change]** |
| --- | --- | --- | --- | --- | --- | --- |
| **Family structure**  **Job stress** | **Males** |  |  |  |  |  |
|  | Single parent family |  |  |  | 1.29 (0.96; 1.73) | 1.19 (0.86; 1.65) |
|  | Couple family (Reference) |  |  |  |  |  |
|  | **Females** |  |  |  |  |  |
|  | Single parent family |  |  |  | 1.00 (0.74; 1.33) | NA |
|  | Couple family (Reference) |  |  |  |  |  |
|  | **Males** |  |  |  |  |  |
|  | Stressed |  |  |  | **0.72 (0.58; 0.90)** | NA |
|  | Not stressed (Reference) |  |  |  |  |  |
|  | **Females** |  |  |  |  |  |
|  | Stressed |  |  |  | 0.93 (0.73; 1.17) | 0.92 (0.72; 1.17) |
|  | Not stressed (Reference) |  |  |  |  |  |
