# Supplementary material for: Socio‐Economic Differences in the Oral Health of Irish Adolescents: The Potential Role of Behavioural, Material and Psychosocial Factors
Source: Community Dent Oral Epidemiol. 2025 Dec 7;54(3):316–32. doi: 10.1111/cdoe.70043 (PMC13146151; doi:10.1111/cdoe.70043)
Supplement: Supplementary file 2 — Table S1: Association between PCGs' highest educational level/family income and young males' self‐reported oral health (self‐rated oral health) adjusted for covariates (Wave 1), behavioural factors, material factors and psychosocial factors (logistic regression odds ratios for self‐rated suboptimal oral health). Model 2: Model 1 + behavioural factors; Model 3: Model 1 + material factors; Model 4: Model 1 + psychosocial factors and Model 5: Model 1 + behavioural factors + material factors + psychosocial factors. All models were adjusted for the ‘area of residence’, the ‘main language spoken at home’ and ‘PCGs' country of birth’. CI, confidence intervals; direct effect, outcome ~ exposure + mediators + covariates; Indirect effect, total effect–direct effect; OR, odds ratio; PCOR, percentage change in odds ratio; Percentage mediated, indirect effect/total effect; total effect: outcome ~ exposure + covariates. Table S2: Association between PCGs' highest educational level/family income/family class/medical card status and young males'/females parent‐reported oral health adjusted for covariates (Wave 1), behavioural factors, material factors and psychosocial factors (logistic regression odds ratios for two teeth with dental fillings and three or more teeth with dental fillings outcomes). Model 2: Model 1 + behavioural factors; Model 3: Model 1 + material factors; Model 4: Model 1 + psychosocial factors; and Model 5: Model 1 + behavioural factors + material factors + psychosocial factors. All models were adjusted for the ‘area of residence’, the ‘main language spoken at home’ and ‘PCGs' country of birth’. CI, confidence intervals; Direct effect, outcome ~ exposure + mediators + covariates; Indirect effect, total effect–direct effect; OR, odds ratio; PCOR, percentage change in odds ratio; percentage mediated, indirect effect/total effect; total effect, Outcome ~ exposure + covariates. Table S3: Association between PCGs' highest educational level/family income and young male [file CDOE-54-316-s001.docx]

**Supplementary Mediation Analysis**

1. **Bootstrap mediation (**5,000 bootstrap iterations; 95% percentile confidence intervals)

Table S1 Association between PCGs’ highest educational level/ family income and young males’ self-reported oral health (self-rated oral health) adjusted for covariates (wave 1), behavioural factors, material factors and psychosocial factors (logistic regression odds ratios for self-rated sub-optimal oral health). Model 2: Model 1 + behavioural factors; Model 3: Model 1 + material factors; Model 4: Model 1 + Psychosocial factors and Model 5: Model 1 + behavioural factors + material factors + Psychosocial factors. All models were adjusted for the ‘area of residence’, the ‘main language spoken at home’ and ‘PCGs’ country of birth’. OR: Odds Ratio; PCOR: Percentage change in Odds Ratio, CI: Confidence intervals, Total effect: Outcome ~ exposure + covariates, Direct effect: Outcome ~ exposure + mediators + covariates, Indirect effect: Total effect – Direct effect, Percentage mediated = Indirect effect/Total effect.

| **SES indicator** | **Exposure level** | **Gender** | **Outcome comparison** | **Model** | **OR total** | **OR adjusted** | **PCOR**  **(95% CI)** | **Total effect** | **Direct effect** | **Indirect effect** | **% mediated** | **N** |
| --- | --- | --- | --- | --- | --- | --- | --- | --- | --- | --- | --- | --- |
| PCG educational level | None or primary | Male | Sub optimum oral health | Model 2 | 2.34 | 2.07 | 24.27  (-102.50 to 150.39) | 0.85 | 0.70 | 0.15 | 17.59 | 2902 |
| PCG educational level | Secondary | Male | Sub optimum oral health | Model 2 | 1.32 | 1.14 | 54.64  (-320.47 to 443.11) | 0.28 | 0.13 | 0.14 | 51.19 | 2902 |
| PCG educational level | None or primary | Male | Sub optimum oral health | Model 3 | 1.51 | 0.95 | 108.91  (-656.76 to 811.94) | 0.41 | -0.04 | 0.45 | 111.27 | 2737 |
| PCG educational level | Secondary | Male | Sub optimum oral health | Model 3 | 1.24 | 0.99 | 100.61 (-714.26 to 862.64) | 0.22 | -0.00 | 0.21 | 100.68 | 2737 |
| PCG educational level | None or primary | Male | Sub optimum oral health | Model 4 | 1.60 | 1.57 | 5.54  (-142.73 to 126.74) | 0.47 | 0.44 | 0.02 | 4.46 | 2763 |
| PCG educational level | Secondary | Male | Sub optimum oral health | Model 4 | 1.29 | 1.29 | 0.70  (-57.86 to 70.98) | 0.26 | 0.26 | 0.00 | 0.62 | 2763 |
| PCG educational level | None or primary | Male | Sub optimum oral health | Model 5 | 1.62 | 1.18 | 72.01  (-386.59 to 592.60) | 0.48 | 0.16 | 0.33 | 66.83 | 2637 |
| PCG educational level | Secondary | Male | Sub optimum oral health | Model 5 | 1.23 | 0.94 | 127.77  (-1135.88 to 1283.03) | 0.21 | -0.07 | 0.27 | 131.89 | 2637 |
| Family Income | Lowest | Male | Sub optimum oral health | Model 2 | 1.65 | 1.22 | 65.46 (-145.76 to 381.13) | 0.49 | 0.20 | 0.30 | 59.58 | 2705 |
| Family Income | 2nd | Male | Sub optimum oral health | Model 2 | 1.89 | 1.46 | **48.32 (19.04 to 173.90)** | 0.64 | 0.38 | 0.26 | 40.55 | 2705 |
| Family Income | 3rd | Male | Sub optimum oral health | Model 2 | 1.16 | 0.94 | 134.73 (-773.20 to 964.26) | 0.15 | -0.06 | 0.21 | 138.62 | 2705 |
| Family Income | 4th | Male | Sub optimum oral health | Model 2 | 1.08 | 0.98 | 120.36 (-525.94 to 527.25) | 0.08 | -0.02 | 0.10 | 121.40 | 2705 |
| Family Income | Lowest | Male | Sub optimum oral health | Model 3 | 1.57 | 1.02 | 96.00 (-366.92 to 573.75) | 0.46 | 0.02 | 0.44 | 94.98 | 2550 |
| Family Income | 2nd | Male | Sub optimum oral health | Model 3 | 1.72 | 1.18 | 74.47 (-3.15 to 324.68) | 0.54 | 0.16 | 0.38 | 70.00 | 2550 |
| Family Income | 3rd | Male | Sub optimum oral health | Model 3 | 1.21 | 0.89 | 152.75 (-906.12 to 1064.14) | 0.19 | -0.12 | 0.31 | 161.65 | 2550 |
| Family Income | 4th | Male | Sub optimum oral health | Model 3 | 0.99 | 0.85 | -1537.27 (-924.89 to 843.53) | -0.00 | -0.16 | 0.15 | -1662.29 | 2550 |
| Family Income | Lowest | Male | Sub optimum oral health | Model 4 | 1.67 | 1.68 | -1.14  (-55.86 to 43.25) | 0.51 | 0.51 | -0.00 | -0.89 | 2581 |
| Family Income | 2nd | Male | Sub optimum oral health | Model 4 | 1.71 | 1.65 | 7.43  (-34.16 to 59.35) | 0.53 | 0.50 | 0.03 | 5.85 | 2581 |
| Family Income | 3rd | Male | Sub optimum oral health | Model 4 | 1.30 | 1.27 | 8.74  (-174.60 to 148.80) | 0.26 | 0.24 | 0.02 | 7.77 | 2581 |
| Family Income | 4th | Male | Sub optimum oral health | Model 4 | 0.99 | 0.97 | -239.24  (-190.06 to 190.02) | -0.01 | -0.03 | 0.02 | -242.84 | 2581 |
| Family Income | Lowest | Male | Sub optimum oral health | Model 5 | 1.51 | 0.87 | 124.55  (-663.93 to 813.52) | 0.41 | -0.13 | 0.55 | 132.51 | 2467 |
| Family Income | 2nd | Male | Sub optimum oral health | Model 5 | 1.62 | 0.99 | 101.03  (-329.45 to 533.57) | 0.48 | -0.00 | 0.49 | 101.32 | 2467 |
| Family Income | 3rd | Male | Sub optimum oral health | Model 5 | 1.09 | 0.72 | 390.63  (-1944.07 to 1761.21) | 0.09 | -0.33 | 0.42 | 457.80 | 2467 |
| Family Income | 4th | Male | Sub optimum oral health | Model 5 | 0.95 | 0.77 | -403.29 (-1144.78 to 1154.23) | -0.05 | -0.26 | 0.22 | -459.06 | 2467 |

Table S2 Association between PCGs’ highest educational level/ family income/ family class/ medical card status and young males’/ females parent-reported oral health adjusted for covariates (wave 1), behavioural factors, material factors and psychosocial factors (logistic regression odds ratios for two teeth with dental fillings and three or more teeth with dental fillings outcomes. Model 2: Model 1 + behavioural factors; Model 3: Model 1 + material factors; Model 4: Model 1 + Psychosocial factors; and Model 5: Model 1 + behavioural factors + material factors + Psychosocial factors. All models were adjusted for the ‘area of residence’, the ‘main language spoken at home’ and ‘PCGs’ country of birth’. OR: Odds Ratio; PCOR: Percentage change in Odds Ratio, CI: Confidence intervals, Total effect: Outcome ~ exposure + covariates, Direct effect: Outcome ~ exposure + mediators + covariates, Indirect effect: Total effect – Direct effect, Percentage mediated = Indirect effect/Total effect.

| **SES Indicator** | **Exposure level** | **Gender** | **Outcome comparison** | **Model** | **OR total** | **OR adjusted** | **PCOR (95% CI)** | **Indirect effect** | **Direct effect** | **Total effect** | **Prop mediated** | **N** |
| --- | --- | --- | --- | --- | --- | --- | --- | --- | --- | --- | --- | --- |
| Family Income | Lowest | Male | 2_fillings_vs_none | Model 2 | 1.77 | 1.79 | -3.07  (-52.80 to 28.76) | -0.01 | 0.58 | 0.57 | -0.02 | 1873 |
| Family Income | 2nd | Male | 2_fillings_vs_none | Model 2 | 1.07 | 1.09 | -27.74  (-345.28 to 324.21) | -0.02 | 0.09 | 0.07 | -0.27 | 1873 |
| Family Income | 3rd | Male | 2_fillings_vs_none | Model 2 | 1.25 | 1.25 | -2.11  (-170.82 to 152.75) | 0.00 | 0.22 | 0.22 | -0.02 | 1873 |
| Family Income | 4th | Male | 2_fillings_vs_none | Model 2 | 1.21 | 1.25 | -20.95  (-186.37 to 174.59) | -0.04 | 0.23 | 0.19 | -0.19 | 1873 |
| Family Income | Lowest | Male | 2_fillings_vs_none | Model 3 | 1.64 | 1.57 | 10.05  (-122.26 to 146.63) | 0.04 | 0.45 | 0.49 | 0.08 | 1755 |
| Family Income | 2nd | Male | 2_fillings_vs_none | Model 3 | 1.05 | 0.96 | 168.88  (-793.19 to 672.73) | 0.09 | -0.04 | 0.05 | 1.72 | 1755 |
| Family Income | 3rd | Male | 2_fillings_vs_none | Model 3 | 1.23 | 1.18 | 22.35  (-308.03 to 341.14) | 0.04 | 0.17 | 0.21 | 0.21 | 1755 |
| Family Income | 4th | Male | 2_fillings_vs_none | Model 3 | 1.18 | 1.16 | 8.53  (-278.98 to 264.87) | 0.01 | 0.15 | 0.16 | 0.08 | 1755 |
| Family Income | Lowest | Male | 2_fillings_vs_none | Model 4 | 1.74 | 1.60 | 18.74  (-7.86 to 86.95) | 0.08 | 0.47 | 0.55 | 0.15 | 1783 |
| Family Income | 2nd | Male | 2_fillings_vs_none | Model 4 | 1.04 | 0.92 | 293.61  (-629.02 to 700.71) | 0.12 | -0.08 | 0.04 | 3.06 | 1783 |
| Family Income | 3rd | Male | 2_fillings_vs_none | Model 4 | 1.29 | 1.21 | 25.62  (-172.52 to 216.99) | 0.06 | 0.19 | 0.25 | 0.23 | 1783 |
| Family Income | 4th | Male | 2_fillings_vs_none | Model 4 | 1.17 | 1.13 | 21.76  (-198.97 to 193.87) | 0.03 | 0.13 | 0.16 | 0.20 | 1783 |
| Family Income | Lowest | Male | 2_fillings_vs_none | Model 5 | 1.63 | 1.67 | -6.77  (-190.41 to 110.74) | -0.03 | 0.51 | 0.49 | -0.05 | 1703 |
| Family Income | 2nd | Male | 2_fillings_vs_none | Model 5 | 0.94 | 0.87 | -110.25  (-709.12 to 902.80) | 0.07 | -0.14 | -0.06 | -1.18 | 1703 |
| Family Income | 3rd | Male | 2_fillings_vs_none | Model 5 | 1.18 | 1.13 | 26.72  (-407.19 to 421.86) | 0.04 | 0.12 | 0.17 | 0.25 | 1703 |
| Family Income | 4th | Male | 2_fillings_vs_none | Model 5 | 1.10 | 1.13 | -26.99  (-464.34 to 384.58) | -0.02 | 0.12 | 0.10 | -0.25 | 1703 |
| Family Income | Lowest | Male | 3plus_fillings_vs_none | Model 2 | 1.60 | 1.64 | -7.06  (-77.07 to 35.73) | -0.03 | 0.50 | 0.47 | -0.06 | 1839 |
| Family Income | 2nd | Male | 3plus_fillings_vs_none | Model 2 | 1.05 | 1.10 | -122.67  (-406.94 to 367.03) | -0.05 | 0.10 | 0.05 | -1.17 | 1839 |
| Family Income | 3rd | Male | 3plus_fillings_vs_none | Model 2 | 1.32 | 1.34 | -5.62  (-142.57 to 125.22) | -0.01 | 0.29 | 0.28 | -0.05 | 1839 |
| Family Income | 4th | Male | 3plus_fillings_vs_none | Model 2 | 1.26 | 1.31 | -19.88  (-231.86 to 193.04) | -0.04 | 0.27 | 0.23 | -0.17 | 1839 |
| Family Income | Lowest | Male | 3plus_fillings_vs_none | Model 3 | 1.60 | 1.28 | 54.00  (-47.67 to 257.80) | 0.23 | 0.25 | 0.47 | 0.48 | 1730 |
| Family Income | 2nd | Male | 3plus_fillings_vs_none | Model 3 | 1.11 | 0.89 | 198.85  (-1022.66 to 1119.12) | 0.22 | -0.12 | 0.11 | 2.11 | 1730 |
| Family Income | 3rd | Male | 3plus_fillings_vs_none | Model 3 | 1.41 | 1.24 | 40.64  (-216.51 to 367.00) | 0.13 | 0.22 | 0.34 | 0.37 | 1730 |
| Family Income | 4th | Male | 3plus_fillings_vs_none | Model 3 | 1.45 | 1.33 | 27.00 (  -124.19 to 176.88) | 0.09 | 0.28 | 0.37 | 0.24 | 1730 |
| Family Income | Lowest | Male | 3plus_fillings_vs_none | Model 4 | 1.64 | 1.52 | 18.90  (-15.43 to 99.04) | 0.08 | 0.42 | 0.49 | 0.16 | 1755 |
| Family Income | 2nd | Male | 3plus_fillings_vs_none | Model 4 | 1.11 | 0.99 | 110.52  (-727.03 to 727.46) | 0.12 | -0.01 | 0.11 | 1.11 | 1755 |
| Family Income | 3rd | Male | 3plus_fillings_vs_none | Model 4 | 1.39 | 1.30 | 25.03  (-144.63 to 187.20) | 0.07 | 0.26 | 0.33 | 0.22 | 1755 |
| Family Income | 4th | Male | 3plus_fillings_vs_none | Model 4 | 1.48 | 1.42 | 12.46  (-46.21 to 114.22) | 0.04 | 0.35 | 0.39 | 0.11 | 1755 |
| Family Income | Lowest | Male | 3plus_fillings_vs_none | Model 5 | 1.50 | 1.29 | 42.69  (-183.82 to 311.40) | 0.15 | 0.25 | 0.41 | 0.38 | 1672 |
| Family Income | 2nd | Male | 3plus_fillings_vs_none | Model 5 | 1.05 | 0.88 | 373.15  (-996.88 to 1295.17) | 0.18 | -0.13 | 0.04 | 3.98 | 1672 |
| Family Income | 3rd | Male | 3plus_fillings_vs_none | Model 5 | 1.34 | 1.19 | 43.35  (-287.42 to 475.96) | 0.12 | 0.18 | 0.30 | 0.40 | 1672 |
| Family Income | 4th | Male | 3plus_fillings_vs_none | Model 5 | 1.22 | 1.18 | 18.29  (-317.37 to 280.92) | 0.03 | 0.17 | 0.20 | 0.17 | 1672 |
| Medical Card | Yes, full card | Male | 2_fillings_vs_none | Model 2 | 1.63 | 1.64 | -2.49  (-31.16 to 18.46) | -0.01 | 0.50 | 0.49 | -0.02 | 2004 |
| Medical Card | Yes, doctor only card | Male | 2_fillings_vs_none | Model 2 | 0.61 | 0.58 | -6.66  (-137.89 to 135.30) | 0.04 | -0.54 | -0.49 | -0.09 | 2004 |
| Medical Card | Yes, full card | Male | 2_fillings_vs_none | Model 3 | 1.61 | 1.71 | -16.89  (-152.33 to 55.60) | -0.06 | 0.54 | 0.47 | -0.13 | 1875 |
| Medical Card | Yes, doctor only card | Male | 2_fillings_vs_none | Model 3 | 0.60 | 0.63 | 7.07  (-155.02 to 177.79) | -0.05 | -0.47 | -0.52 | 0.09 | 1875 |
| Medical Card | Yes, full card | Male | 2_fillings_vs_none | Model 4 | 1.64 | 1.43 | 31.71  (-7.21 to 95.80) | 0.13 | 0.36 | 0.49 | 0.27 | 1901 |
| Medical Card | Yes, doctor only card | Male | 2_fillings_vs_none | Model 4 | 0.60 | 0.60 | -0.65  (-73.31 to 91.35) | 0.00 | -0.51 | -0.51 | -0.01 | 1901 |
| Medical Card | Yes, full card | Male | 2_fillings_vs_none | Model 5 | 1.60 | 1.59 | 2.54  (-116.19 to 91.36) | 0.01 | 0.46 | 0.47 | 0.02 | 1812 |
| Medical Card | Yes, doctor only card | Male | 2_fillings_vs_none | Model 5 | 0.58 | 0.60 | 3.43  (-180.57 to 215.13) | -0.02 | -0.51 | -0.54 | 0.04 | 1812 |
| Medical Card | Yes, full card | Male | 3plus_fillings_vs_none | Model 2 | 1.41 | 1.45 | -9.90  (-73.13 to 25.43) | -0.03 | 0.37 | 0.34 | -0.08 | 1979 |
| Medical Card | Yes, doctor only card | Male | 3plus_fillings_vs_none | Model 2 | 0.50 | 0.50 | 0.30  (-58.75 to 65.89) | 0.00 | -0.69 | -0.70 | 0.00 | 1979 |
| Medical Card | Yes, full card | Male | 3plus_fillings_vs_none | Model 3 | 1.46 | 1.39 | 14.84  (-99.81 to 163.11) | 0.05 | 0.33 | 0.38 | 0.13 | 1861 |
| Medical Card | Yes, doctor only card | Male | 3plus_fillings_vs_none | Model 3 | 0.48 | 0.44 | -6.92  (-91.62 to 54.00) | 0.08 | -0.82 | -0.74 | -0.11 | 1861 |
| Medical Card | Yes, full card | Male | 3plus_fillings_vs_none | Model 4 | 1.51 | 1.38 | 25.65  (-19.84 to 115.20) | 0.09 | 0.32 | 0.41 | 0.22 | 1886 |
| Medical Card | Yes, doctor only card | Male | 3plus_fillings_vs_none | Model 4 | 0.48 | 0.48 | -0.75  (-45.14 to 46.41) | 0.01 | -0.74 | -0.74 | -0.01 | 1886 |
| Medical Card | Yes, full card | Male | 3plus_fillings_vs_none | Model 5 | 1.42 | 1.39 | 6.04  (-157.19 to 148.61) | 0.02 | 0.33 | 0.35 | 0.05 | 1791 |
| Medical Card | Yes, doctor only card | Male | 3plus_fillings_vs_none | Model 5 | 0.47 | 0.44 | -5.44  (-96.42 to 81.53) | 0.06 | -0.82 | -0.75 | -0.08 | 1791 |
| PCG Education | None or primary | Female | 2_fillings_vs_none | Model 2 | 3.26 | 3.39 | -5.66  (-75.96 to 22.51) | -0.04 | 1.22 | 1.18 | -0.03 | 2051 |
| PCG Education | Secondary | Female | 2_fillings_vs_none | Model 2 | 1.46 | 1.46 | 0.59  (-36.54 to 36.79) | 0.00 | 0.38 | 0.38 | 0.00 | 2051 |
| PCG Education | None or primary | Female | 2_fillings_vs_none | Model 3 | 3.19 | 2.85 | 15.61  (-43.09 to 81.00) | 0.11 | 1.05 | 1.16 | 0.10 | 1905 |
| PCG Education | Secondary | Female | 2_fillings_vs_none | Model 3 | 1.59 | 1.52 | 11.60  (-27.03 to 56.63) | 0.04 | 0.42 | 0.46 | 0.09 | 1905 |
| PCG Education | None or primary | Female | 2_fillings_vs_none | Model 4 | 3.26 | 3.10 | 7.29  (-25.15 to 40.46) | 0.05 | 1.13 | 1.18 | 0.04 | 1954 |
| PCG Education | Secondary | Female | 2_fillings_vs_none | Model 4 | 1.50 | 1.47 | 5.78  (-9.19 to 31.71) | 0.02 | 0.39 | 0.41 | 0.05 | 1954 |
| PCG Education | None or primary | Female | 2_fillings_vs_none | Model 5 | 3.46 | 3.19 | 10.88  (-86.40 to 67.83) | 0.08 | 1.16 | 1.24 | 0.06 | 1847 |
| PCG Education | Secondary | Female | 2_fillings_vs_none | Model 5 | 1.61 | 1.52 | 14.59  (-26.69 to 63.79) | 0.06 | 0.42 | 0.48 | 0.12 | 1847 |
| PCG Education | None or primary | Female | 3plus_fillings_vs_none | Model 2 | 1.92 | 2.02 | -10.21  (-193.27 to 148.69) | -0.05 | 0.70 | 0.65 | -0.07 | 2000 |
| PCG Education | Secondary | Female | 3plus_fillings_vs_none | Model 2 | 1.28 | 1.26 | 7.85  (-109.63 to 129.48) | 0.02 | 0.23 | 0.25 | 0.07 | 2000 |
| PCG Education | None or primary | Female | 3plus_fillings_vs_none | Model 3 | 2.16 | 1.81 | 30.04  (-176.67 to 278.09) | 0.18 | 0.59 | 0.77 | 0.23 | 1851 |
| PCG Education | Secondary | Female | 3plus_fillings_vs_none | Model 3 | 1.25 | 1.15 | 40.11  (-264.70 to 299.05) | 0.08 | 0.14 | 0.22 | 0.38 | 1851 |
| PCG Education | None or primary | Female | 3plus_fillings_vs_none | Model 4 | 2.21 | 2.12 | 7.49  (-92.70 to 114.99) | 0.04 | 0.75 | 0.79 | 0.05 | 1895 |
| PCG Education | Secondary | Female | 3plus_fillings_vs_none | Model 4 | 1.25 | 1.21 | 16.17  (-99.39 to 159.03) | 0.03 | 0.19 | 0.22 | 0.15 | 1895 |
| PCG Education | None or primary | Female | 3plus_fillings_vs_none | Model 5 | 2.25 | 1.92 | 26.51  (-249.52 to 248.49) | 0.16 | 0.65 | 0.81 | 0.20 | 1790 |
| PCG Education | Secondary | Female | 3plus_fillings_vs_none | Model 5 | 1.26 | 1.16 | 41.16  (-297.35 to 393.53) | 0.09 | 0.14 | 0.23 | 0.38 | 1790 |
| Family Income | Lowest | Female | 2_fillings_vs_none | Model 2 | 1.77 | 1.85 | -10.53  (-72.40 to 18.55) | -0.04 | 0.62 | 0.57 | -0.08 | 1897 |
| Family Income | 2nd | Female | 2_fillings_vs_none | Model 2 | 1.61 | 1.69 | -12.18  (-98.83 to 24.51) | -0.05 | 0.52 | 0.48 | -0.09 | 1897 |
| Family Income | 3rd | Female | 2_fillings_vs_none | Model 2 | 1.67 | 1.66 | 1.22  (-42.30 to 37.34) | 0.00 | 0.51 | 0.51 | 0.01 | 1897 |
| Family Income | 4th | Female | 2_fillings_vs_none | Model 2 | 0.96 | 0.97 | 30.70  (-252.01 to 196.79) | -0.01 | -0.03 | -0.04 | 0.31 | 1897 |
| Family Income | Lowest | Female | 2_fillings_vs_none | Model 3 | 1.82 | 1.89 | -8.24  (-121.39 to 67.96) | -0.04 | 0.64 | 0.60 | -0.06 | 1765 |
| Family Income | 2nd | Female | 2_fillings_vs_none | Model 3 | 1.69 | 1.76 | -9.28  (-142.61 to 70.62) | -0.04 | 0.56 | 0.53 | -0.07 | 1765 |
| Family Income | 3rd | Female | 2_fillings_vs_none | Model 3 | 1.77 | 1.82 | -6.79  (-102.15 to 48.65) | -0.03 | 0.60 | 0.57 | -0.05 | 1765 |
| Family Income | 4th | Female | 2_fillings_vs_none | Model 3 | 1.04 | 1.06 | -52.43  (-278.42 to 272.08) | -0.02 | 0.05 | 0.04 | -0.51 | 1765 |
| Family Income | Lowest | Female | 2_fillings_vs_none | Model 4 | 1.75 | 1.75 | 0.20  (-47.27 to 43.18) | 0.00 | 0.56 | 0.56 | 0.00 | 1807 |
| Family Income | 2nd | Female | 2_fillings_vs_none | Model 4 | 1.69 | 1.64 | 6.85  (-23.33 to 43.32) | 0.03 | 0.50 | 0.53 | 0.05 | 1807 |
| Family Income | 3rd | Female | 2_fillings_vs_none | Model 4 | 1.63 | 1.60 | 4.71  (-24.20 to 42.19) | 0.02 | 0.47 | 0.49 | 0.04 | 1807 |
| Family Income | 4th | Female | 2_fillings_vs_none | Model 4 | 0.97 | 0.96 | -25.85  (-118.28 to 122.01) | 0.01 | -0.04 | -0.03 | -0.26 | 1807 |
| Family Income | Lowest | Female | 2_fillings_vs_none | Model 5 | 1.86 | 2.00 | -16.49  (-151.67 to 56.28) | -0.07 | 0.69 | 0.62 | -0.12 | 1713 |
| Family Income | 2nd | Female | 2_fillings_vs_none | Model 5 | 1.76 | 1.93 | -21.84  (-172.68 to 48.53) | -0.09 | 0.66 | 0.57 | -0.16 | 1713 |
| Family Income | 3rd | Female | 2_fillings_vs_none | Model 5 | 1.82 | 1.85 | -4.23  (-99.75 to 50.45) | -0.02 | 0.62 | 0.60 | -0.03 | 1713 |
| Family Income | 4th | Female | 2_fillings_vs_none | Model 5 | 1.04 | 1.09 | -153.20  (-505.83 to 479.97) | -0.05 | 0.09 | 0.03 | -1.47 | 1713 |
| Family Income | Lowest | Female | 3plus_fillings_vs_none | Model 2 | 1.82 | 1.75 | 9.06  (-24.75 to 56.38) | 0.04 | 0.56 | 0.60 | 0.07 | 1845 |
| Family Income | 2nd | Female | 3plus_fillings_vs_none | Model 2 | 1.01 | 0.99 | 220.61  (-436.94 to 352.78) | 0.02 | -0.01 | 0.01 | 2.22 | 1845 |
| Family Income | 3rd | Female | 3plus_fillings_vs_none | Model 2 | 1.46 | 1.47 | -2.88  (-90.93 to 63.75) | -0.01 | 0.39 | 0.38 | -0.02 | 1845 |
| Family Income | 4th | Female | 3plus_fillings_vs_none | Model 2 | 1.07 | 1.06 | 15.48  (-249.76 to 236.61) | 0.01 | 0.06 | 0.07 | 0.15 | 1845 |
| Family Income | Lowest | Female | 3plus_fillings_vs_none | Model 3 | 1.85 | 1.39 | 54.68  (-11.51 to 185.03) | 0.29 | 0.33 | 0.62 | 0.47 | 1710 |
| Family Income | 2nd | Female | 3plus_fillings_vs_none | Model 3 | 0.99 | 0.77 | -2750.80  (-1437.45 to 1240.34) | 0.25 | -0.26 | -0.01 | -31.18 | 1710 |
| Family Income | 3rd | Female | 3plus_fillings_vs_none | Model 3 | 1.50 | 1.24 | 51.81  (-106.24 to 330.98) | 0.19 | 0.22 | 0.41 | 0.47 | 1710 |
| Family Income | 4th | Female | 3plus_fillings_vs_none | Model 3 | 1.00 | 0.94 | 23383.78  (-361.88 to 471.45) | 0.06 | -0.06 | 0.00 | 241.24 | 1710 |
| Family Income | Lowest | Female | 3plus_fillings_vs_none | Model 4 | 1.76 | 1.66 | 12.41  (-22.50 to 72.67) | 0.05 | 0.51 | 0.56 | 0.10 | 1748 |
| Family Income | 2nd | Female | 3plus_fillings_vs_none | Model 4 | 0.98 | 0.95 | -112.13  (-290.48 to 291.66) | 0.03 | -0.05 | -0.02 | -1.15 | 1748 |
| Family Income | 3rd | Female | 3plus_fillings_vs_none | Model 4 | 1.49 | 1.42 | 14.07  (-57.93 to 103.17) | 0.05 | 0.35 | 0.40 | 0.12 | 1748 |
| Family Income | 4th | Female | 3plus_fillings_vs_none | Model 4 | 1.01 | 1.00 | 59.06  (-159.58 to 131.19) | 0.01 | 0.00 | 0.01 | 0.59 | 1748 |
| Family Income | Lowest | Female | 3plus_fillings_vs_none | Model 5 | 1.85 | 1.35 | 58.24  (-12.39 to 216.61) | 0.31 | 0.30 | 0.61 | 0.51 | 1656 |
| Family Income | 2nd | Female | 3plus_fillings_vs_none | Model 5 | 1.01 | 0.83 | 2451.27  (-1109.27 to 1262.72) | 0.20 | -0.19 | 0.01 | 26.90 | 1656 |
| Family Income | 3rd | Female | 3plus_fillings_vs_none | Model 5 | 1.64 | 1.38 | 40.80  (-29.33 to 161.95) | 0.17 | 0.32 | 0.50 | 0.35 | 1656 |
| Family Income | 4th | Female | 3plus_fillings_vs_none | Model 5 | 1.01 | 0.98 | 259.16  (-395.73 to 412.83) | 0.03 | -0.02 | 0.01 | 2.62 | 1656 |
| Family Occupation | Semi-skilled/Unskilled | Female | 2_fillings_vs_none | Model 2 | 1.72 | 1.74 | -2.30  (-51.67 to 28.27) | -0.01 | 0.55 | 0.54 | -0.02 | 1946 |
| Family Occupation | Non manual/Skilled | Female | 2_fillings_vs_none | Model 2 | 1.17 | 1.17 | 2.06  (-197.58 to 172.33) | 0.00 | 0.15 | 0.16 | 0.02 | 1946 |
| Family Occupation | Semi-skilled/Unskilled | Female | 2_fillings_vs_none | Model 3 | 1.80 | 1.50 | 37.80  (-25.93 to 164.49) | 0.18 | 0.40 | 0.59 | 0.31 | 1808 |
| Family Occupation | Non manual/Skilled | Female | 2_fillings_vs_none | Model 3 | 1.26 | 1.14 | 44.34  (-302.51 to 378.36) | 0.09 | 0.13 | 0.23 | 0.42 | 1808 |
| Family Occupation | Semi-skilled/Unskilled | Female | 2_fillings_vs_none | Model 4 | 1.79 | 1.65 | 17.95  (-7.57 to 64.82) | 0.08 | 0.50 | 0.58 | 0.14 | 1852 |
| Family Occupation | Non manual/Skilled | Female | 2_fillings_vs_none | Model 4 | 1.21 | 1.16 | 25.79  (-183.80 to 228.48) | 0.05 | 0.15 | 0.19 | 0.24 | 1852 |
| Family Occupation | Semi-skilled/Unskilled | Female | 2_fillings_vs_none | Model 5 | 1.80 | 1.49 | 38.90  (-43.43 to 155.13) | 0.19 | 0.40 | 0.59 | 0.32 | 1751 |
| Family Occupation | Non manual/Skilled | Female | 2_fillings_vs_none | Model 5 | 1.25 | 1.14 | 42.96  (-237.42 to 403.89) | 0.09 | 0.13 | 0.22 | 0.40 | 1751 |
| Family Occupation | Semi-skilled/Unskilled | Female | 3plus_fillings_vs_none | Model 2 | 1.62 | 1.63 | -1.09  (-69.64 to 57.90) | 0.00 | 0.49 | 0.48 | -0.01 | 1892 |
| Family Occupation | Non manual/Skilled | Female | 3plus_fillings_vs_none | Model 2 | 1.39 | 1.32 | 18.06  (-17.33 to 97.85) | 0.05 | 0.28 | 0.33 | 0.16 | 1892 |
| Family Occupation | Semi-skilled/Unskilled | Female | 3plus_fillings_vs_none | Model 3 | 1.49 | 1.34 | 30.75  (-147.59 to 257.48) | 0.11 | 0.29 | 0.40 | 0.27 | 1755 |
| Family Occupation | Non manual/Skilled | Female | 3plus_fillings_vs_none | Model 3 | 1.41 | 1.28 | 31.77  (-43.21 to 198.12) | 0.10 | 0.25 | 0.35 | 0.28 | 1755 |
| Family Occupation | Semi-skilled/Unskilled | Female | 3plus_fillings_vs_none | Model 4 | 1.52 | 1.52 | -0.87  (-69.91 to 62.92) | 0.00 | 0.42 | 0.42 | -0.01 | 1792 |
| Family Occupation | Non manual/Skilled | Female | 3plus_fillings_vs_none | Model 4 | 1.35 | 1.32 | 7.62  (-41.72 to 70.60) | 0.02 | 0.28 | 0.30 | 0.07 | 1792 |
| Family Occupation | Semi-skilled/Unskilled | Female | 3plus_fillings_vs_none | Model 5 | 1.47 | 1.35 | 25.22  (-247.57 to 281.20) | 0.08 | 0.30 | 0.39 | 0.22 | 1694 |
| Family Occupation | Non manual/Skilled | Female | 3plus_fillings_vs_none | Model 5 | 1.37 | 1.17 | 53.47  (-90.41 to 314.41) | 0.16 | 0.16 | 0.31 | 0.50 | 1694 |
| Medical Card | Yes, full card | Female | 2_fillings_vs_none | Model 2 | 1.43 | 1.48 | -11.86  (-84.36 to 27.14) | -0.04 | 0.40 | 0.36 | -0.10 | 2050 |
| Medical Card | Yes, doctor only card | Female | 2_fillings_vs_none | Model 2 | 1.45 | 1.63 | -39.74  (-284.42 to 200.58) | -0.12 | 0.49 | 0.37 | -0.31 | 2050 |
| Medical Card | Yes, full card | Female | 2_fillings_vs_none | Model 3 | 1.43 | 1.28 | 35.45  (-81.58 to 195.42) | 0.11 | 0.24 | 0.36 | 0.32 | 1904 |
| Medical Card | Yes, doctor only card | Female | 2_fillings_vs_none | Model 3 | 1.41 | 1.27 | 34.47  (-250.47 to 290.39) | 0.11 | 0.24 | 0.35 | 0.31 | 1904 |
| Medical Card | Yes, full card | Female | 2_fillings_vs_none | Model 4 | 1.44 | 1.41 | 6.47  (-67.87 to 79.37) | 0.02 | 0.35 | 0.37 | 0.05 | 1953 |
| Medical Card | Yes, doctor only card | Female | 2_fillings_vs_none | Model 4 | 1.38 | 1.36 | 4.79  (-134.62 to 134.75) | 0.01 | 0.31 | 0.32 | 0.04 | 1953 |
| Medical Card | Yes, full card | Female | 2_fillings_vs_none | Model 5 | 1.51 | 1.43 | 15.08  (-126.55 to 127.37) | 0.05 | 0.36 | 0.41 | 0.13 | 1846 |
| Medical Card | Yes, doctor only card | Female | 2_fillings_vs_none | Model 5 | 1.45 | 1.51 | -13.74  (-404.30 to 265.23) | -0.04 | 0.41 | 0.37 | -0.11 | 1846 |
| Medical Card | Yes, full card | Female | 3plus_fillings_vs_none | Model 2 | 1.64 | 1.64 | 1.30  (-33.99 to 34.92) | 0.01 | 0.49 | 0.50 | 0.01 | 1999 |
| Medical Card | Yes, doctor only card | Female | 3plus_fillings_vs_none | Model 2 | 1.26 | 1.29 | -12.48  (-209.02 to 185.18) | -0.03 | 0.25 | 0.23 | -0.11 | 1999 |
| Medical Card | Yes, full card | Female | 3plus_fillings_vs_none | Model 3 | 1.54 | 1.35 | 35.56  (-59.97 to 158.32) | 0.13 | 0.30 | 0.43 | 0.31 | 1850 |
| Medical Card | Yes, doctor only card | Female | 3plus_fillings_vs_none | Model 3 | 1.27 | 1.11 | 58.62  (-309.05 to 374.96) | 0.13 | 0.11 | 0.24 | 0.56 | 1850 |
| Medical Card | Yes, full card | Female | 3plus_fillings_vs_none | Model 4 | 1.53 | 1.48 | 10.67  (-50.52 to 79.87) | 0.04 | 0.39 | 0.43 | 0.09 | 1894 |
| Medical Card | Yes, doctor only card | Female | 3plus_fillings_vs_none | Model 4 | 0.87 | 0.87 | 2.24  (-133.32 to 154.36) | 0.00 | -0.13 | -0.14 | 0.02 | 1894 |
| Medical Card | Yes, full card | Female | 3plus_fillings_vs_none | Model 5 | 1.58 | 1.35 | 40.68  (-60.98 to 165.24) | 0.16 | 0.30 | 0.46 | 0.35 | 1789 |
| Medical Card | Yes, doctor only card | Female | 3plus_fillings_vs_none | Model 5 | 0.84 | 0.75 | -51.44  (-404.55 to 397.25) | 0.11 | -0.28 | -0.18 | -0.59 | 1789 |

1. **Causal mediation analysis results (Imai, Keele and Tingley, 2010), g-computation approach, Quasi-Bayesian simulation (1,000 iterations)**

Table S3 Association between PCGs’ highest educational level/ family income and young males’ self-reported oral health (self-rated oral health) adjusted for covariates (wave 1), behavioural factors, material factors and psychosocial factors (logistic regression odds ratios for self-rated sub-optimal oral health) (Causal mediation approach, simulations = 1000). Model 2: Model 1 + behavioural factors; Model 3: Model 1 + material factors; Model 4: Model 1 + Psychosocial factors and Model 5: Model 1 + behavioural factors + material factors + Psychosocial factors. All models were adjusted for the ‘area of residence’, the ‘main language spoken at home’ and ‘PCGs’ country of birth’. OR: Odds Ratio; PCOR: Percentage change in Odds Ratio, CI: Confidence intervals, Total effect: Outcome ~ exposure + covariates, Direct effect: Outcome ~ exposure + mediators + covariates, Indirect effect: Total effect – Direct effect, Percentage mediated = Indirect effect/Total effect, NIE: Natural indirect effect, NDE: Natural direct effect.

| **Exposure**  **Level** | **Mediator**  **Set** | **N** | **OR**  **Total** | **OR**  **Adjusted** | **PCOR** | **Total**  **Effect** | **Direct**  **Effect** | **Indirect**  **Effect** | **Percentage**  **Mediated** | **95% CI NIE** | **95% CI NDE** |
| --- | --- | --- | --- | --- | --- | --- | --- | --- | --- | --- | --- |
| **PCG Higher educational level (Reference level: Tertiary)** | | | |  |  |  |  |  |  |  |  |
| **None or primary** | Model 2: Behavioural | 1055 | 1.094 | 1.076 | 18.59 | 0.0894 | 0.0734 | 0.0091 | 10.23 | -0.0092 to 0.0348 | -0.0392 to 0.2246 |
| **Secondary** | Model 2: Behavioural | 2863 | 1.022 | 1.014 | 35.26 | 0.0216 | 0.0141 | 0.0066 | 30.37 | 0.0026 to 0.0118 | -0.0155 to 0.2246 |
| **None or primary** | Model 3: Material | 995 | 1.039 | 1.042 | -8.1 | 0.0383 | 0.0414 | -0.0021 | -5.55 | -0.0155 to 0.0069 | -0.0643 to 0.0423 |
| **Secondary** | Model 3: Material | 2702 | 1.018 | 1.015 | 12.58 | 0.0176 | 0.0154 | 0.0019 | 10.76 | -0.0001 to 0.0049 | -0.0167 to 0.176 |
| **None or primary** | Model 4: Psychosocial | 1003 | 1.046 | 1.046 | -0.26 | 0.0446 | 0.0447 | -1E-04 | -0.17 | -0.0151 to 0.0127 | -0.0583 to 0.0455 |
| **Secondary** | Model 4: Psychosocial | 2731 | 1.022 | 1.021 | 3.28 | 0.0217 | 0.021 | 6E-04 | 2.66 | -0.0009 to 0.0026 | -0.0096 to 0.197 |
| **None or primary** | Model 3: All | 948 | 1.042 | 1.046 | -7.64 | 0.0416 | 0.0447 | -0.0021 | -5.04 | -0.0176 to 0.0083 | -0.0609 to 0.05 |
| **Secondary** | Model 3: All | 2605 | 1.017 | 1.013 | 24.73 | 0.0166 | 0.0125 | 0.0036 | 21.72 | 0.0007 to 0.0074 | -0.0181 to 0.1951 |
| **Family income quintile (Reference level: Highest)** | | | |  |  |  |  |  |  |  |  |
| **Lowest** | Model 2: Behavioural | 1118 | 1.044 | 1.028 | 34.94 | 0.0426 | 0.0279 | 0.0113 | 26.57 | 0.003 to 0.0222 | -0.0155 to 0.0774 |
| **2nd** | Model 2: Behavioural | 1170 | 1.069 | 1.06 | 13.01 | 0.0663 | 0.0579 | 0.0052 | 7.86 | -0.0024 to 0.0149 | 0.006 to 0.1102 |
| **3rd** | Model 2: Behavioural | 1202 | 1.01 | 1.006 | 35.64 | 0.0099 | 0.0064 | 0.0033 | 33.2 | -0.0019 to 0.0105 | -0.0363 to 0.0537 |
| **4th** | Model 2: Behavioural | 1402 | 1.007 | 1.004 | 45.33 | 0.0068 | 0.0037 | 0.0029 | 43.44 | -0.0005 to 0.0081 | -0.0317 to 0.0429 |
| **Lowest** | Model 3: Material | 1071 | 1.041 | 1.032 | 21.7 | 0.0404 | 0.0318 | 0.0065 | 16.11 | 0.0007 to 0.0147 | -0.0158 to 0.0844 |
| **2nd** | Model 3: Material | 1103 | 1.058 | 1.056 | 4.46 | 0.0567 | 0.0543 | 0.0016 | 2.77 | -0.0013 to 0.0054 | 0.0026 to 0.113 |
| **3rd** | Model 3: Material | 1128 | 1.012 | 1.006 | 53.77 | 0.0122 | 0.0057 | 0.0062 | 50.61 | -0.0005 to 0.015 | -0.0396 to 0.0553 |
| **4th** | Model 3: Material | 1315 | 0.999 | 1.001 | 253.7 | -7E-04 | 0.001 | -0.0017 | 250.7 | -0.0083 to 0.0013 | -0.0351 to 0.037 |
| **Lowest** | Model 4: Psychosocial | 1072 | 1.048 | 1.039 | 18.55 | 0.0466 | 0.0381 | 0.0061 | 13.03 | 0.0005 to 0.0141 | -0.0083 to 0.089 |
| **2nd** | Model 4: Psychosocial | 1114 | 1.058 | 1.054 | 6.69 | 0.056 | 0.0523 | 0.0023 | 4.19 | -0.0053 to 0.0114 | 0.0005 to 0.1028 |
| **3rd** | Model 4: Psychosocial | 1144 | 1.022 | 1.022 | -0.65 | 0.0216 | 0.0217 | -1E-04 | -0.52 | -0.0059 to 0.005 | -0.0257 to 0.073 |
| **4th** | Model 4: Psychosocial | 1333 | 1.001 | 1.002 | -49.26 | 0.001 | 0.0015 | -5E-04 | -48.41 | -0.0033 to 0.0011 | -0.0371 to 0.0369 |
| **Lowest** | Model 3: All | 1028 | 1.038 | 1.031 | 16.76 | 0.037 | 0.0309 | 0.0047 | 12.63 | 0 to 0.0133 | -0.0196 to 0.0841 |
| **2nd** | Model 3: All | 1059 | 1.051 | 1.05 | 1.98 | 0.0502 | 0.0492 | 6E-04 | 1.28 | -0.0024 to 0.0039 | -0.0022 to 0.1077 |
| **3rd** | Model 3: All | 1088 | 1.006 | 1.004 | 41.17 | 0.006 | 0.0035 | 0.0024 | 39.62 | -0.0016 to 0.0078 | -0.042 to 0.0525 |
| **4th** | Model 3: All | 1266 | 0.997 | 0.996 | -42.73 | -0.003 | -0.0037 | 0.0011 | -44.57 | -0.0046 to 0.0059 | -0.0389 to 0.0362 |

Table S4 Association between PCGs’ highest educational level/ family income/ family class/ medical card status and young males’/ females parent-reported oral health adjusted for covariates (wave 1), behavioural factors, material factors and psychosocial factors (logistic regression odds ratios for two teeth with dental fillings and three or more teeth with dental fillings outcomes (Causal mediation approach, simulations = 1000). Model 2: Model 1 + behavioural factors; Model 3: Model 1 + material factors; Model 4: Model 1 + Psychosocial factors; and Model 5: Model 1 + behavioural factors + material factors + Psychosocial factors. All models were adjusted for the ‘area of residence’, the ‘main language spoken at home’ and ‘PCGs’ country of birth’. OR: Odds Ratio; PCOR: Percentage change in Odds Ratio, CI: Confidence intervals, Total effect: Outcome ~ exposure + covariates, Direct effect: Outcome ~ exposure + mediators + covariates, Indirect effect: Total effect – Direct effect, Percentage mediated = Indirect effect/Total effect.

| **Exposure** | **Exposure Level** | **Gender** | **Outcome Comparison** | **Model** | **N** | **OR Total** | **OR Adjusted** | **PCOR** | **Total Effect** | **Indirect Effect (95% CI)** | **Direct Effect (95% CI)** | **% Mediated** |
| --- | --- | --- | --- | --- | --- | --- | --- | --- | --- | --- | --- | --- |
| **Family Income** | Lowest | Male | 2_fillings_vs_none | Model 2: Behavioural | 694 | 1.079 | 1.075 | 5.25 | 0.0764 | 0.0034 (-0.0092, 0.0170) | 0.0725 (0.0087, 0.1383) | 3.67% |
| **Family Income** | 2nd | Male | 2_fillings_vs_none | Model 2: Behavioural | 729 | 0.991 | 0.989 | -23.57 | -0.0091 | 0.0023 (-0.0067, 0.0127) | -0.0112 (-0.0757, 0.0509) | -2.40% |
| **Family Income** | 3rd | Male | 2_fillings_vs_none | Model 2: Behavioural | 765 | 1.037 | 1.034 | 7.81 | 0.0362 | 0.0025 (-0.0014, 0.0093) | 0.0334 (-0.0290, 0.1018) | 3.88% |
| **Family Income** | 4th | Male | 2_fillings_vs_none | Model 2: Behavioural | 886 | 1.012 | 1.014 | -14.73 | 0.0122 | -0.0017 (-0.0070, 0.0017) | 0.0139 (-0.0451, 0.0768) | -1.18% |
| **Family Income** | Lowest | Male | 2_fillings_vs_none | Model 3: Material | 694 | 1.080 | 1.079 | 1.06 | 0.0770 | 0.0007 (-0.0067, 0.0080) | 0.0763 (0.0116, 0.1402) | 0.62% |
| **Family Income** | 2nd | Male | 2_fillings_vs_none | Model 3: Material | 729 | 0.991 | 0.991 | -1.81 | -0.0093 | 0.0002 (-0.0058, 0.0065) | -0.0095 (-0.0756, 0.0576) | -0.59% |
| **Family Income** | 3rd | Male | 2_fillings_vs_none | Model 3: Material | 765 | 1.035 | 1.027 | 20.99 | 0.0340 | 0.0065 (-0.0000, 0.0157) | 0.0270 (-0.0366, 0.0884) | 11.52% |
| **Family Income** | 4th | Male | 2_fillings_vs_none | Model 3: Material | 886 | 1.013 | 1.013 | -6.18 | 0.0126 | -0.0007 (-0.0047, 0.0023) | 0.0134 (-0.0504, 0.0764) | -0.46% |
| **Family Income** | Lowest | Male | 2_fillings_vs_none | Model 4: Psychosocial | 694 | 1.079 | 1.069 | 12.88 | 0.0759 | 0.0081 (0.0000, 0.0190) | 0.0665 (0.0048, 0.1293) | 9.81% |
| **Family Income** | 2nd | Male | 2_fillings_vs_none | Model 4: Psychosocial | 729 | 0.992 | 0.993 | 10.82 | -0.0083 | -0.0007 (-0.0151, 0.0140) | -0.0074 (-0.0760, 0.0583) | 1.14% |
| **Family Income** | 3rd | Male | 2_fillings_vs_none | Model 4: Psychosocial | 765 | 1.037 | 1.027 | 25.85 | 0.0361 | 0.0086 (0.0017, 0.0189) | 0.0269 (-0.0398, 0.0934) | 16.11% |
| **Family Income** | 4th | Male | 2_fillings_vs_none | Model 4: Psychosocial | 886 | 1.014 | 1.014 | -0.12 | 0.0143 | -0.0000 (-0.0023, 0.0024) | 0.0143 (-0.0464, 0.0760) | 0.00% |
| **Family Income** | Lowest | Male | 2_fillings_vs_none | Model 5: All Mediators | 694 | 1.081 | 1.084 | -4.41 | 0.0778 | -0.0027 (-0.0093, 0.0024) | 0.0811 (0.0162, 0.1464) | -3.06% |
| **Family Income** | 2nd | Male | 2_fillings_vs_none | Model 5: All Mediators | 729 | 0.992 | 0.992 | 2.64 | -0.0079 | -0.0002 (-0.0039, 0.0026) | -0.0077 (-0.0721, 0.0538) | 0.08% |
| **Family Income** | 3rd | Male | 2_fillings_vs_none | Model 5: All Mediators | 765 | 1.039 | 1.040 | -1.13 | 0.0387 | -0.0004 (-0.0046, 0.0032) | 0.0391 (-0.0201, 0.1019) | -0.22% |
| **Family Income** | 4th | Male | 2_fillings_vs_none | Model 5: All Mediators | 886 | 1.014 | 1.014 | -0.43 | 0.0136 | -0.0001 (-0.0023, 0.0026) | 0.0137 (-0.0470, 0.0754) | 0.03% |
| **Family Income** | Lowest | Male | 3plus_fillings_vs_none | Model 2: Behavioural | 674 | 1.070 | 1.092 | -31.72 | 0.0678 | -0.0158 (-0.0303, -0.0043) | 0.0884 (0.0214, 0.1554) | -21.76% |
| **Family Income** | 2nd | Male | 3plus_fillings_vs_none | Model 2: Behavioural | 711 | 1.007 | 1.012 | -75.46 | 0.0069 | -0.0048 (-0.0160, 0.0056) | 0.0120 (-0.0492, 0.0744) | -2.72% |
| **Family Income** | 3rd | Male | 3plus_fillings_vs_none | Model 2: Behavioural | 742 | 1.072 | 1.072 | 0.30 | 0.0693 | 0.0002 (-0.0066, 0.0073) | 0.0691 (0.0036, 0.1375) | 0.25% |
| **Family Income** | 4th | Male | 3plus_fillings_vs_none | Model 2: Behavioural | 847 | 1.030 | 1.029 | 4.41 | 0.0300 | 0.0012 (-0.0073, 0.0098) | 0.0287 (-0.0358, 0.0884) | 2.58% |
| **Family Income** | Lowest | Male | 3plus_fillings_vs_none | Model 3: Material | 674 | 1.072 | 1.074 | -4.02 | 0.0691 | -0.0021 (-0.0078, 0.0014) | 0.0718 (0.0102, 0.1309) | -2.34% |
| **Family Income** | 2nd | Male | 3plus_fillings_vs_none | Model 3: Material | 711 | 1.007 | 1.007 | -5.45 | 0.0070 | -0.0003 (-0.0059, 0.0051) | 0.0073 (-0.0591, 0.0656) | -0.12% |
| **Family Income** | 3rd | Male | 3plus_fillings_vs_none | Model 3: Material | 742 | 1.068 | 1.054 | 21.15 | 0.0662 | 0.0115 (0.0030, 0.0229) | 0.0526 (-0.0092, 0.1161) | 16.61% |
| **Family Income** | 4th | Male | 3plus_fillings_vs_none | Model 3: Material | 847 | 1.031 | 1.030 | 4.60 | 0.0310 | 0.0013 (-0.0033, 0.0068) | 0.0296 (-0.0315, 0.0874) | 1.88% |
| **Family Income** | Lowest | Male | 3plus_fillings_vs_none | Model 4: Psychosocial | 674 | 1.070 | 1.069 | 1.54 | 0.0675 | 0.0009 (-0.0073, 0.0097) | 0.0664 (0.0005, 0.1335) | 1.03% |
| **Family Income** | 2nd | Male | 3plus_fillings_vs_none | Model 4: Psychosocial | 711 | 1.008 | 1.022 | -185.31 | 0.0078 | -0.0132 (-0.0284, 0.0007) | 0.0222 (-0.0404, 0.0872) | -16.72% |
| **Family Income** | 3rd | Male | 3plus_fillings_vs_none | Model 4: Psychosocial | 742 | 1.070 | 1.060 | 14.46 | 0.0674 | 0.0079 (0.0012, 0.0170) | 0.0579 (-0.0099, 0.1190) | 10.52% |
| **Family Income** | 4th | Male | 3plus_fillings_vs_none | Model 4: Psychosocial | 847 | 1.031 | 1.031 | -0.84 | 0.0304 | -0.0002 (-0.0032, 0.0023) | 0.0307 (-0.0302, 0.0881) | -0.20% |
| **Family Income** | Lowest | Male | 3plus_fillings_vs_none | Model 5: All Mediators | 674 | 1.068 | 1.074 | -8.84 | 0.0661 | -0.0045 (-0.0121, 0.0012) | 0.0717 (0.0067, 0.1399) | -5.88% |
| **Family Income** | 2nd | Male | 3plus_fillings_vs_none | Model 5: All Mediators | 711 | 1.006 | 1.006 | 3.13 | 0.0061 | 0.0002 (-0.0024, 0.0034) | 0.0059 (-0.0605, 0.0687) | 0.10% |
| **Family Income** | 3rd | Male | 3plus_fillings_vs_none | Model 5: All Mediators | 742 | 1.070 | 1.069 | 0.89 | 0.0673 | 0.0005 (-0.0053, 0.0060) | 0.0667 (0.0003, 0.1302) | 0.42% |
| **Family Income** | 4th | Male | 3plus_fillings_vs_none | Model 5: All Mediators | 847 | 1.031 | 1.031 | -0.06 | 0.0307 | -0.0000 (-0.0025, 0.0022) | 0.0307 (-0.0266, 0.0928) | 0.00% |
| **Medical Card** | Yes, full card | Male | 2_fillings_vs_none | Model 2: Behavioural | 1767 | 1.094 | 1.098 | -4.78 | 0.0898 | -0.0032 (-0.0090, 0.0017) | 0.0939 (0.0479, 0.1403) | -3.48% |
| **Medical Card** | Yes, doctor only card | Male | 2_fillings_vs_none | Model 2: Behavioural | 1422 | 0.930 | 0.929 | -0.45 | -0.0728 | 0.0005 (-0.0069, 0.0083) | -0.0731 (-0.1598, 0.0556) | -0.27% |
| **Medical Card** | Yes, full card | Male | 2_fillings_vs_none | Model 3: Material | 1767 | 1.093 | 1.087 | 5.59 | 0.0885 | 0.0039 (-0.0066, 0.0143) | 0.0838 (0.0379, 0.1299) | 4.21% |
| **Medical Card** | Yes, doctor only card | Male | 2_fillings_vs_none | Model 3: Material | 1422 | 0.929 | 0.930 | 1.55 | -0.0741 | -0.0017 (-0.0111, 0.0070) | -0.0729 (-0.1621, 0.0458) | 1.25% |
| **Medical Card** | Yes, full card | Male | 2_fillings_vs_none | Model 4: Psychosocial | 1767 | 1.093 | 1.079 | 14.84 | 0.0885 | 0.0106 (-0.0034, 0.0244) | 0.0759 (0.0315, 0.1203) | 11.91% |
| **Medical Card** | Yes, doctor only card | Male | 2_fillings_vs_none | Model 4: Psychosocial | 1422 | 0.927 | 0.926 | -0.81 | -0.0762 | 0.0009 (-0.0035, 0.0069) | -0.0769 (-0.1600, 0.0458) | -0.37% |
| **Medical Card** | Yes, full card | Male | 2_fillings_vs_none | Model 5: All Mediators | 1767 | 1.092 | 1.093 | -0.24 | 0.0884 | -0.0002 (-0.0021, 0.0014) | 0.0886 (0.0433, 0.1338) | -0.06% |
| **Medical Card** | Yes, doctor only card | Male | 2_fillings_vs_none | Model 5: All Mediators | 1422 | 0.928 | 0.928 | -0.24 | -0.0744 | 0.0003 (-0.0040, 0.0053) | -0.0746 (-0.1594, 0.0430) | -0.12% |
| **Medical Card** | Yes, full card | Male | 3plus_fillings_vs_none | Model 2: Behavioural | 1747 | 1.067 | 1.068 | -1.61 | 0.0649 | -0.0008 (-0.0068, 0.0050) | 0.0659 (0.0220, 0.1099) | -1.04% |
| **Medical Card** | Yes, doctor only card | Male | 3plus_fillings_vs_none | Model 2: Behavioural | 1407 | 0.913 | 0.912 | -0.72 | -0.0911 | 0.0011 (-0.0056, 0.0091) | -0.0918 (-0.1680, 0.0265) | -0.62% |
| **Medical Card** | Yes, full card | Male | 3plus_fillings_vs_none | Model 3: Material | 1747 | 1.067 | 1.076 | -12.97 | 0.0653 | -0.0066 (-0.0167, 0.0041) | 0.0734 (0.0286, 0.1175) | -9.87% |
| **Medical Card** | Yes, doctor only card | Male | 3plus_fillings_vs_none | Model 3: Material | 1407 | 0.910 | 0.908 | -3.03 | -0.0939 | 0.0050 (-0.0045, 0.0171) | -0.0969 (-0.1703, 0.0167) | -4.05% |
| **Medical Card** | Yes, full card | Male | 3plus_fillings_vs_none | Model 4: Psychosocial | 1747 | 1.068 | 1.061 | 9.80 | 0.0659 | 0.0054 (-0.0061, 0.0179) | 0.0597 (0.0152, 0.1055) | 7.97% |
| **Medical Card** | Yes, doctor only card | Male | 3plus_fillings_vs_none | Model 4: Psychosocial | 1407 | 0.913 | 0.914 | 1.29 | -0.0911 | -0.0019 (-0.0084, 0.0028) | -0.0899 (-0.1642, 0.0284) | 1.10% |
| **Medical Card** | Yes, full card | Male | 3plus_fillings_vs_none | Model 5: All Mediators | 1747 | 1.066 | 1.069 | -3.28 | 0.0642 | -0.0017 (-0.0048, 0.0005) | 0.0663 (0.0218, 0.1098) | -2.41% |
| **Medical Card** | Yes, doctor only card | Male | 3plus_fillings_vs_none | Model 5: All Mediators | 1407 | 0.912 | 0.912 | 0.16 | -0.0921 | -0.0002 (-0.0058, 0.0057) | -0.0919 (-0.1683, 0.0297) | 0.17% |
| **PCG Education** | None or primary | Female | 2_fillings_vs_none | Model 2: Behavioural | 627 | 1.318 | 1.322 | -1.28 | 0.2759 | -0.0016 (-0.0298, 0.0280) | 0.2790 (0.1463, 0.4150) | -0.64% |
| **PCG Education** | Secondary | Female | 2_fillings_vs_none | Model 2: Behavioural | 1825 | 1.090 | 1.090 | -0.08 | 0.0864 | -0.0000 (-0.0055, 0.0052) | 0.0865 (0.0323, 0.1328) | -0.10% |
| **PCG Education** | None or primary | Female | 2_fillings_vs_none | Model 3: Material | 627 | 1.316 | 1.324 | -2.56 | 0.2746 | -0.0039 (-0.0189, 0.0107) | 0.2807 (0.1489, 0.4259) | -1.33% |
| **PCG Education** | Secondary | Female | 2_fillings_vs_none | Model 3: Material | 1825 | 1.091 | 1.090 | 0.54 | 0.0867 | 0.0004 (-0.0011, 0.0022) | 0.0862 (0.0365, 0.1343) | 0.23% |
| **PCG Education** | None or primary | Female | 2_fillings_vs_none | Model 4: Psychosocial | 627 | 1.310 | 1.353 | -14.04 | 0.2700 | -0.0202 (-0.0524, 0.0115) | 0.3027 (0.1698, 0.4383) | -7.64% |
| **PCG Education** | Secondary | Female | 2_fillings_vs_none | Model 4: Psychosocial | 1825 | 1.091 | 1.087 | 4.16 | 0.0867 | 0.0028 (-0.0000, 0.0067) | 0.0833 (0.0318, 0.1299) | 3.03% |
| **PCG Education** | None or primary | Female | 2_fillings_vs_none | Model 5: All Mediators | 627 | 1.310 | 1.309 | 0.38 | 0.2703 | 0.0006 (-0.0059, 0.0081) | 0.2694 (0.1425, 0.4036) | 0.07% |
| **PCG Education** | Secondary | Female | 2_fillings_vs_none | Model 5: All Mediators | 1825 | 1.091 | 1.092 | -0.93 | 0.0868 | -0.0006 (-0.0033, 0.0012) | 0.0876 (0.0367, 0.1395) | -0.42% |
| **PCG Education** | None or primary | Female | 3plus_fillings_vs_none | Model 2: Behavioural | 604 | 1.194 | 1.173 | 10.78 | 0.1775 | 0.0138 (-0.0095, 0.0436) | 0.1598 (0.0292, 0.3058) | 7.39% |
| **PCG Education** | Secondary | Female | 3plus_fillings_vs_none | Model 2: Behavioural | 1771 | 1.041 | 1.036 | 12.43 | 0.0401 | 0.0045 (0.0001, 0.0104) | 0.0352 (-0.0193, 0.0845) | 8.66% |
| **PCG Education** | None or primary | Female | 3plus_fillings_vs_none | Model 3: Material | 604 | 1.194 | 1.196 | -1.29 | 0.1772 | -0.0015 (-0.0095, 0.0050) | 0.1793 (0.0488, 0.3111) | -0.46% |
| **PCG Education** | Secondary | Female | 3plus_fillings_vs_none | Model 3: Material | 1771 | 1.040 | 1.039 | 2.37 | 0.0389 | 0.0008 (-0.0012, 0.0040) | 0.0380 (-0.0165, 0.0877) | 1.25% |
| **PCG Education** | None or primary | Female | 3plus_fillings_vs_none | Model 4: Psychosocial | 604 | 1.192 | 1.229 | -19.14 | 0.1757 | -0.0207 (-0.0485, 0.0040) | 0.2061 (0.0625, 0.3501) | -11.41% |
| **PCG Education** | Secondary | Female | 3plus_fillings_vs_none | Model 4: Psychosocial | 1771 | 1.039 | 1.034 | 11.31 | 0.0379 | 0.0039 (0.0003, 0.0091) | 0.0337 (-0.0171, 0.0831) | 8.07% |
| **PCG Education** | None or primary | Female | 3plus_fillings_vs_none | Model 5: All Mediators | 604 | 1.189 | 1.191 | -0.79 | 0.1733 | -0.0009 (-0.0110, 0.0077) | 0.1746 (0.0449, 0.3111) | -0.24% |
| **PCG Education** | Secondary | Female | 3plus_fillings_vs_none | Model 5: All Mediators | 1771 | 1.041 | 1.041 | 0.45 | 0.0403 | 0.0002 (-0.0013, 0.0021) | 0.0401 (-0.0093, 0.0908) | 0.14% |
| **Family Income** | Lowest | Female | 2_fillings_vs_none | Model 2: Behavioural | 702 | 1.129 | 1.137 | -5.86 | 0.1217 | -0.0049 (-0.0199, 0.0090) | 0.1284 (0.0509, 0.2003) | -3.86% |
| **Family Income** | 2nd | Female | 2_fillings_vs_none | Model 2: Behavioural | 734 | 1.119 | 1.122 | -2.49 | 0.1122 | -0.0019 (-0.0154, 0.0129) | 0.1149 (0.0461, 0.1823) | -1.72% |
| **Family Income** | 3rd | Female | 2_fillings_vs_none | Model 2: Behavioural | 799 | 1.123 | 1.116 | 5.39 | 0.1161 | 0.0048 (-0.0105, 0.0199) | 0.1102 (0.0391, 0.1772) | 3.86% |
| **Family Income** | 4th | Female | 2_fillings_vs_none | Model 2: Behavioural | 840 | 0.996 | 0.998 | 55.88 | -0.0040 | -0.0022 (-0.0148, 0.0091) | -0.0018 (-0.0691, 0.0659) | -0.46% |
| **Family Income** | Lowest | Female | 2_fillings_vs_none | Model 3: Material | 702 | 1.127 | 1.131 | -2.78 | 0.1199 | -0.0023 (-0.0120, 0.0071) | 0.1230 (0.0540, 0.1954) | -1.64% |
| **Family Income** | 2nd | Female | 2_fillings_vs_none | Model 3: Material | 734 | 1.118 | 1.108 | 8.45 | 0.1112 | 0.0072 (-0.0029, 0.0184) | 0.1023 (0.0372, 0.1656) | 6.10% |
| **Family Income** | 3rd | Female | 2_fillings_vs_none | Model 3: Material | 799 | 1.121 | 1.110 | 9.57 | 0.1145 | 0.0083 (0.0010, 0.0186) | 0.1041 (0.0313, 0.1785) | 6.88% |
| **Family Income** | 4th | Female | 2_fillings_vs_none | Model 3: Material | 840 | 0.997 | 0.997 | 1.88 | -0.0031 | -0.0001 (-0.0039, 0.0036) | -0.0031 (-0.0649, 0.0647) | -0.09% |
| **Family Income** | Lowest | Female | 2_fillings_vs_none | Model 4: Psychosocial | 702 | 1.127 | 1.128 | -0.32 | 0.1197 | -0.0001 (-0.0195, 0.0198) | 0.1201 (0.0422, 0.1962) | 0.10% |
| **Family Income** | 2nd | Female | 2_fillings_vs_none | Model 4: Psychosocial | 734 | 1.118 | 1.106 | 10.05 | 0.1117 | 0.0086 (-0.0044, 0.0239) | 0.1011 (0.0292, 0.1727) | 7.15% |
| **Family Income** | 3rd | Female | 2_fillings_vs_none | Model 4: Psychosocial | 799 | 1.121 | 1.110 | 9.03 | 0.1141 | 0.0079 (-0.0042, 0.0219) | 0.1043 (0.0359, 0.1749) | 6.75% |
| **Family Income** | 4th | Female | 2_fillings_vs_none | Model 4: Psychosocial | 840 | 0.993 | 0.993 | -3.19 | -0.0066 | 0.0002 (-0.0032, 0.0042) | -0.0068 (-0.0730, 0.0646) | 0.01% |
| **Family Income** | Lowest | Female | 2_fillings_vs_none | Model 5: All Mediators | 702 | 1.127 | 1.128 | -0.12 | 0.1199 | -0.0001 (-0.0033, 0.0026) | 0.1201 (0.0499, 0.1889) | -0.01% |
| **Family Income** | 2nd | Female | 2_fillings_vs_none | Model 5: All Mediators | 734 | 1.120 | 1.121 | -0.89 | 0.1131 | -0.0007 (-0.0078, 0.0059) | 0.1140 (0.0430, 0.1785) | -0.59% |
| **Family Income** | 3rd | Female | 2_fillings_vs_none | Model 5: All Mediators | 799 | 1.119 | 1.116 | 2.22 | 0.1125 | 0.0019 (-0.0027, 0.0077) | 0.1101 (0.0426, 0.1789) | 1.36% |
| **Family Income** | 4th | Female | 2_fillings_vs_none | Model 5: All Mediators | 840 | 0.998 | 1.002 | 233.82 | -0.0019 | -0.0043 (-0.0148, 0.0044) | 0.0025 (-0.0709, 0.0736) | 0.62% |
| **Family Income** | Lowest | Female | 3plus_fillings_vs_none | Model 2: Behavioural | 694 | 1.127 | 1.122 | 4.33 | 0.1198 | 0.0040 (-0.0101, 0.0204) | 0.1149 (0.0423, 0.1887) | 3.28% |
| **Family Income** | 2nd | Female | 3plus_fillings_vs_none | Model 2: Behavioural | 698 | 1.006 | 0.985 | 334.51 | 0.0063 | 0.0224 (0.0041, 0.0429) | -0.0149 (-0.0823, 0.0559) | 27.68% |
| **Family Income** | 3rd | Female | 3plus_fillings_vs_none | Model 2: Behavioural | 786 | 1.089 | 1.087 | 1.78 | 0.0848 | 0.0013 (-0.0110, 0.0152) | 0.0834 (0.0084, 0.1543) | 1.29% |
| **Family Income** | 4th | Female | 3plus_fillings_vs_none | Model 2: Behavioural | 825 | 1.000 | 1.001 | -530.42 | 0.0002 | -0.0010 (-0.0121, 0.0105) | 0.0013 (-0.0687, 0.0726) | 0.20% |
| **Family Income** | Lowest | Female | 3plus_fillings_vs_none | Model 3: Material | 694 | 1.131 | 1.126 | 3.62 | 0.1228 | 0.0034 (-0.0100, 0.0180) | 0.1186 (0.0483, 0.1938) | 2.67% |
| **Family Income** | 2nd | Female | 3plus_fillings_vs_none | Model 3: Material | 698 | 1.007 | 1.010 | -35.37 | 0.0072 | -0.0024 (-0.0137, 0.0074) | 0.0097 (-0.0611, 0.0769) | -0.81% |
| **Family Income** | 3rd | Female | 3plus_fillings_vs_none | Model 3: Material | 786 | 1.090 | 1.084 | 6.97 | 0.0860 | 0.0048 (-0.0025, 0.0148) | 0.0802 (0.0065, 0.1530) | 4.91% |
| **Family Income** | 4th | Female | 3plus_fillings_vs_none | Model 3: Material | 825 | 0.998 | 0.997 | -55.59 | -0.0020 | 0.0011 (-0.0045, 0.0083) | -0.0031 (-0.0749, 0.0635) | -0.38% |
| **Family Income** | Lowest | Female | 3plus_fillings_vs_none | Model 4: Psychosocial | 694 | 1.128 | 1.139 | -9.04 | 0.1201 | -0.0075 (-0.0248, 0.0090) | 0.1303 (0.0588, 0.2066) | -6.07% |
| **Family Income** | 2nd | Female | 3plus_fillings_vs_none | Model 4: Psychosocial | 698 | 1.006 | 0.998 | 125.02 | 0.0063 | 0.0080 (-0.0063, 0.0236) | -0.0016 (-0.0677, 0.0664) | 5.05% |
| **Family Income** | 3rd | Female | 3plus_fillings_vs_none | Model 4: Psychosocial | 786 | 1.089 | 1.073 | 18.15 | 0.0852 | 0.0128 (-0.0003, 0.0287) | 0.0703 (0.0020, 0.1436) | 14.71% |
| **Family Income** | 4th | Female | 3plus_fillings_vs_none | Model 4: Psychosocial | 825 | 1.001 | 0.997 | 501.34 | 0.0006 | 0.0033 (-0.0022, 0.0105) | -0.0026 (-0.0688, 0.0625) | 0.29% |
| **Family Income** | Lowest | Female | 3plus_fillings_vs_none | Model 5: All Mediators | 694 | 1.128 | 1.124 | 3.13 | 0.1205 | 0.0028 (-0.0017, 0.0097) | 0.1169 (0.0404, 0.1912) | 1.88% |
| **Family Income** | 2nd | Female | 3plus_fillings_vs_none | Model 5: All Mediators | 698 | 1.006 | 1.004 | 32.95 | 0.0059 | 0.0019 (-0.0049, 0.0100) | 0.0039 (-0.0619, 0.0689) | 0.54% |
| **Family Income** | 3rd | Female | 3plus_fillings_vs_none | Model 5: All Mediators | 786 | 1.088 | 1.089 | -1.10 | 0.0844 | -0.0007 (-0.0050, 0.0026) | 0.0852 (0.0183, 0.1511) | -0.39% |
| **Family Income** | 4th | Female | 3plus_fillings_vs_none | Model 5: All Mediators | 825 | 1.001 | 1.006 | -733.59 | 0.0007 | -0.0051 (-0.0148, 0.0021) | 0.0060 (-0.0637, 0.0719) | 1.32% |
| **Family Occupation** | Semi-skilled/Unskilled | Female | 2_fillings_vs_none | Model 2: Behavioural | 1199 | 1.134 | 1.138 | -3.01 | 0.1261 | -0.0028 (-0.0131, 0.0070) | 0.1297 (0.0627, 0.1944) | -2.08% |
| **Family Occupation** | Non manual/Skilled | Female | 2_fillings_vs_none | Model 2: Behavioural | 1596 | 1.044 | 1.042 | 3.60 | 0.0430 | 0.0014 (-0.0056, 0.0082) | 0.0415 (-0.0028, 0.0915) | 3.12% |
| **Family Occupation** | Semi-skilled/Unskilled | Female | 2_fillings_vs_none | Model 3: Material | 1199 | 1.135 | 1.127 | 5.92 | 0.1266 | 0.0058 (-0.0038, 0.0169) | 0.1195 (0.0526, 0.1919) | 4.60% |
| **Family Occupation** | Non manual/Skilled | Female | 2_fillings_vs_none | Model 3: Material | 1596 | 1.042 | 1.043 | -0.46 | 0.0415 | -0.0002 (-0.0028, 0.0024) | 0.0416 (-0.0053, 0.0884) | -0.19% |
| **Family Occupation** | Semi-skilled/Unskilled | Female | 2_fillings_vs_none | Model 4: Psychosocial | 1199 | 1.133 | 1.107 | 19.55 | 0.1247 | 0.0193 (0.0058, 0.0348) | 0.1015 (0.0395, 0.1662) | 15.43% |
| **Family Occupation** | Non manual/Skilled | Female | 2_fillings_vs_none | Model 4: Psychosocial | 1596 | 1.044 | 1.037 | 14.64 | 0.0429 | 0.0057 (-0.0010, 0.0130) | 0.0368 (-0.0110, 0.0835) | 12.15% |
| **Family Occupation** | Semi-skilled/Unskilled | Female | 2_fillings_vs_none | Model 5: All Mediators | 1199 | 1.132 | 1.134 | -1.96 | 0.1236 | -0.0018 (-0.0065, 0.0012) | 0.1259 (0.0604, 0.1927) | -1.15% |
| **Family Occupation** | Non manual/Skilled | Female | 2_fillings_vs_none | Model 5: All Mediators | 1596 | 1.042 | 1.043 | -1.14 | 0.0414 | -0.0004 (-0.0025, 0.0010) | 0.0418 (-0.0048, 0.0884) | -0.43% |
| **Family Occupation** | Semi-skilled/Unskilled | Female | 3plus_fillings_vs_none | Model 2: Behavioural | 1155 | 1.076 | 1.075 | 1.39 | 0.0732 | 0.0008 (-0.0052, 0.0080) | 0.0722 (0.0078, 0.1430) | 0.92% |
| **Family Occupation** | Non manual/Skilled | Female | 3plus_fillings_vs_none | Model 2: Behavioural | 1544 | 1.059 | 1.049 | 16.83 | 0.0571 | 0.0083 (0.0022, 0.0158) | 0.0477 (0.0013, 0.0945) | 14.14% |
| **Family Occupation** | Semi-skilled/Unskilled | Female | 3plus_fillings_vs_none | Model 3: Material | 1155 | 1.074 | 1.080 | -7.97 | 0.0712 | -0.0045 (-0.0143, 0.0048) | 0.0766 (0.0097, 0.1442) | -5.88% |
| **Family Occupation** | Non manual/Skilled | Female | 3plus_fillings_vs_none | Model 3: Material | 1544 | 1.059 | 1.060 | -2.20 | 0.0572 | -0.0010 (-0.0050, 0.0026) | 0.0584 (0.0080, 0.1070) | -1.55% |
| **Family Occupation** | Semi-skilled/Unskilled | Female | 3plus_fillings_vs_none | Model 4: Psychosocial | 1155 | 1.075 | 1.070 | 5.69 | 0.0720 | 0.0034 (-0.0053, 0.0123) | 0.0681 (0.0027, 0.1339) | 4.37% |
| **Family Occupation** | Non manual/Skilled | Female | 3plus_fillings_vs_none | Model 4: Psychosocial | 1544 | 1.061 | 1.056 | 7.36 | 0.0591 | 0.0037 (-0.0023, 0.0108) | 0.0549 (0.0069, 0.1035) | 5.82% |
| **Family Occupation** | Semi-skilled/Unskilled | Female | 3plus_fillings_vs_none | Model 5: All Mediators | 1155 | 1.077 | 1.079 | -1.87 | 0.0746 | -0.0011 (-0.0051, 0.0016) | 0.0760 (0.0090, 0.1479) | -0.98% |
| **Family Occupation** | Non manual/Skilled | Female | 3plus_fillings_vs_none | Model 5: All Mediators | 1544 | 1.058 | 1.057 | 1.14 | 0.0565 | 0.0005 (-0.0013, 0.0032) | 0.0558 (0.0065, 0.1051) | 0.61% |
| **Medical Card** | Yes, full card | Female | 2_fillings_vs_none | Model 2: Behavioural | 1805 | 1.088 | 1.086 | 1.45 | 0.0841 | 0.0010 (-0.0067, 0.0094) | 0.0829 (0.0349, 0.1281) | 1.11% |
| **Medical Card** | Yes, doctor only card | Female | 2_fillings_vs_none | Model 2: Behavioural | 1434 | 1.075 | 1.071 | 5.26 | 0.0721 | 0.0033 (-0.0096, 0.0182) | 0.0685 (-0.0615, 0.2107) | 2.97% |
| **Medical Card** | Yes, full card | Female | 2_fillings_vs_none | Model 3: Material | 1805 | 1.088 | 1.087 | 1.58 | 0.0845 | 0.0012 (-0.0092, 0.0113) | 0.0832 (0.0376, 0.1302) | 1.30% |
| **Medical Card** | Yes, doctor only card | Female | 2_fillings_vs_none | Model 3: Material | 1434 | 1.076 | 1.088 | -16.06 | 0.0732 | -0.0095 (-0.0339, 0.0145) | 0.0845 (-0.0536, 0.2507) | -7.32% |
| **Medical Card** | Yes, full card | Female | 2_fillings_vs_none | Model 4: Psychosocial | 1805 | 1.087 | 1.081 | 6.97 | 0.0838 | 0.0050 (-0.0098, 0.0195) | 0.0782 (0.0263, 0.1311) | 5.86% |
| **Medical Card** | Yes, doctor only card | Female | 2_fillings_vs_none | Model 4: Psychosocial | 1434 | 1.081 | 1.062 | 23.02 | 0.0778 | 0.0159 (-0.0006, 0.0372) | 0.0604 (-0.0689, 0.2154) | 13.45% |
| **Medical Card** | Yes, full card | Female | 2_fillings_vs_none | Model 5: All Mediators | 1805 | 1.088 | 1.088 | -0.04 | 0.0842 | -0.0000 (-0.0012, 0.0011) | 0.0843 (0.0370, 0.1328) | -0.01% |
| **Medical Card** | Yes, doctor only card | Female | 2_fillings_vs_none | Model 5: All Mediators | 1434 | 1.082 | 1.092 | -12.07 | 0.0791 | -0.0078 (-0.0220, 0.0029) | 0.0883 (-0.0407, 0.2582) | -6.52% |
| **Medical Card** | Yes, full card | Female | 3plus_fillings_vs_none | Model 2: Behavioural | 1752 | 1.095 | 1.087 | 8.80 | 0.0906 | 0.0064 (-0.0001, 0.0141) | 0.0830 (0.0391, 0.1268) | 6.60% |
| **Medical Card** | Yes, doctor only card | Female | 3plus_fillings_vs_none | Model 2: Behavioural | 1388 | 0.971 | 0.965 | -22.36 | -0.0294 | 0.0078 (-0.0012, 0.0218) | -0.0361 (-0.1418, 0.1130) | -5.84% |
| **Medical Card** | Yes, full card | Female | 3plus_fillings_vs_none | Model 3: Material | 1752 | 1.096 | 1.091 | 5.33 | 0.0919 | 0.0039 (-0.0057, 0.0141) | 0.0872 (0.0423, 0.1343) | 4.19% |
| **Medical Card** | Yes, doctor only card | Female | 3plus_fillings_vs_none | Model 3: Material | 1388 | 0.975 | 0.983 | 33.65 | -0.0258 | -0.0094 (-0.0288, 0.0070) | -0.0170 (-0.1378, 0.1427) | 7.69% |
| **Medical Card** | Yes, full card | Female | 3plus_fillings_vs_none | Model 4: Psychosocial | 1752 | 1.095 | 1.090 | 5.08 | 0.0905 | 0.0038 (-0.0100, 0.0186) | 0.0861 (0.0395, 0.1365) | 4.03% |
| **Medical Card** | Yes, doctor only card | Female | 3plus_fillings_vs_none | Model 4: Psychosocial | 1388 | 0.975 | 0.967 | -33.11 | -0.0251 | 0.0098 (-0.0030, 0.0277) | -0.0336 (-0.1444, 0.1410) | -6.65% |
| **Medical Card** | Yes, full card | Female | 3plus_fillings_vs_none | Model 5: All Mediators | 1752 | 1.094 | 1.094 | 0.05 | 0.0899 | 0.0000 (-0.0016, 0.0017) | 0.0899 (0.0462, 0.1362) | 0.00% |
| **Medical Card** | Yes, doctor only card | Female | 3plus_fillings_vs_none | Model 5: All Mediators | 1388 | 0.975 | 0.975 | 3.07 | -0.0257 | -0.0009 (-0.0083, 0.0061) | -0.0249 (-0.1408, 0.1335) | 0.42% |

Table S5 Comparison of mediation estimates across three analytic approaches for potential mediation of behavioural/material/psychosocial factors in the relationship between self-reported oral health and PCG’s highest educational level and Family income quintiles. PCOR: Percentage change in Odds Ratio.

| **Exposure levels** | **Mediator Model** | **Baron & Kenny** | **Bootstrap** | **Causal Mediation** |
| --- | --- | --- | --- | --- |
| **Primary care giver highest education level (Reference: Tertiary)** |  | **PCOR** | **PCOR (95% CI)** | **PCOR** |
| None or Primary | Model 2: Behavioural | 22.1% | 24.3% (-103, 150) | 18.59% |
|  | Model 3: Material | 100.0% | 108.9% (-657, 812) | -8.0% |
|  | Model 4: Psychosocial | 56.5% | 5.5% (-143, 127) | -0.3% |
|  | Model 5: All Mediators | 87.8% | 72.0% (-387, 593) | -5.0% |
| Secondary | Model 2: Behavioural | 57.6% | 54.6% (-320, 443) | 35.3%* |
|  | Model 3: Material | 100.0% | 100.6% (-714, 863) | 12.6% |
|  | Model 4: Psychosocial | 12.1% | 0.7% (-58, 71) | 3.3% |
|  | Model 5: All Mediators | 100.0% | 127.8% (-1136, 1283) | 24.7% |
| **Family Income Quintiles (Reference: Highest)** |  |  |  |  |
| Lowest | Model 2: Behavioural | 69.4% | 65.5% (-146, 381) | 34.9% |
|  | Model 3: Material | 97.2% | 96.0% (-367, 574) | 21.7% |
|  | Model 4: Psychosocial | 5.6% | -1.1% (-56, 43) | 18.5% |
|  | Model 5: All Mediators | 100.0% | 124.6% (-664, 814) | 16.7% |
| 2nd Quintile | Model 2: Behavioural | 49.5% | 48.3% (19, 174)* | 13.0% |
|  | Model 3: Material | 80.2% | 74.5% (-3, 325) | 4.4% |
|  | Model 4: Psychosocial | 28.6% | 7.4% (-34, 59) | 6.6% |
|  | Model 5: All Mediators | 100.0% | 101.0% (-329, 534) | 1.9% |
| 3rd Quintile | Model 2: Behavioural | 100.0% | 134.7% (-773, 964) | 35.6% |
|  | Model 3: Material | 100.0% | 152.8% (-906, 1064) | 53.8% |
|  | Model 4: Psychosocial | -8.0% | 8.7% (-175, 149) | -0.6% |
|  | Model 5: All Mediators | 100.0% | 390.6% (-1944, 1761) | 41.2% |
| 4th Quintile | Model 2: Behavioural | 100.0% | 120.4% (-526, 527) | 45.0% |
|  | Model 3: Material | 100.0% | -1537.3% (-925, 844) | 253.7% |
|  | Model 4: Psychosocial | 100.0% | -239.2% (-190, 190) | -49.3% |
|  | Model 5: All Mediators | 100.0% | -403.3% (-1145, 1154) | -42.7% |

*Note:* PCOR = ((OR _Reference model_  - OR _Reference + more_ )/( OR _Reference model_  - 1)) X 100

*Where OR _Reference model_  = OR obtained in the first model, OR _Reference + more_ = OR obtained after adding additional variables in the reference model and PCOR = Percentage change in the magnitude of the first OR. Detailed confidence intervals for causal mediation natural indirect effects (NIE) are available in Table S6.*

Table S6 Comparison of mediation estimates across three analytic approaches for potential mediation of behavioural/material/psychosocial factors in the relationship between parent-reported oral health (Outcome: Two teeth with dental fillings) and PCG’s highest educational level/Family income quintiles/Family occupation/Medical card status among males and females. PCOR: Percentage change in Odds Ratio.

| **Exposure levels** | **Mediator Model** | **Baron & Kenny** | **Bootstrap** | **Causal Mediation** |
| --- | --- | --- | --- | --- |
| **Primary care giver highest education level (Reference: Tertiary)** |  | **PCOR (%)** | **PCOR (95% CI)** | **PCOR (%)** |
| **Females** |  |  |  |  |
| None or Primary | Model 2: Behavioural | -2.99 | -5.66 (-75.96 to 22.51) | -1.28 |
|  | Model 3: Material | 17.95 | 15.61 (-43.09 to 81.00) | -2.56 |
|  | Model 4: Psychosocial | 2.56 | 7.29 (-25.15 to 40.46) | -14.04 |
|  | Model 5: All Mediators | -23.08 | 10.88 (-86.40 to 67.83) | 0.38 |
| Secondary | Model 2: Behavioural | -2.13 | 0.59 (-36.54 to 36.79) | -0.08 |
|  | Model 3: Material | -10.64 | 11.60 (-27.03 to 56.63) | 0.54 |
|  | Model 4: Psychosocial | -4.26 | 5.78 (-9.19 to 31.71) | 4.16 |
|  | Model 5: All Mediators | -44.68 | 14.59 (-26.69 to 63.79) | -0.93 |
| **Family Income Quintiles (Reference: Highest)** |  |  |  |  |
| **Males** |  |  |  |  |
| Lowest | Model 2: Behavioural | -1.22 | -3.07 (-52.80 to 28.76) | 5.25 |
|  | Model 3: Material | 26.83 | 10.05 (-122.26 to 146.63) | 1.06 |
|  | Model 4: Psychosocial | 29.27 | 18.74 (-7.86 to 86.95) | 12.88 |
|  | Model 5: All Mediators | 20.73 | -6.77 (-190.41 to 110.74) | -4.41 |
| 2nd Quintile | Model 2: Behavioural | 0.00 | -27.74 (-345.28 to 324.21) | -23.57 |
|  | Model 3: Material | 100.00 | 168.88 (-793.19 to 672.73) | -1.81 |
|  | Model 4: Psychosocial | 100.00 | 293.61 (-629.02 to 700.71) | 10.82 |
|  | Model 5: All Mediators | 100.00 | -110.25 (-709.12 to 902.80) | 2.64 |
| 3rd Quintile | Model 2: Behavioural | 23.53 | -2.11 (-170.82 to 152.75) | 7.81 |
|  | Model 3: Material | 47.06 | 22.35 (-308.03 to 341.14) | 20.99 |
|  | Model 4: Psychosocial | 50.00 | 25.62 (-172.52 to 216.99) | 25.85 |
|  | Model 5: All Mediators | 64.71 | 26.72 (-407.19 to 421.86) | -1.13 |
| 4th Quintile | Model 2: Behavioural | -8.00 | -20.95 (-186.37 to 174.59) | -14.73 |
|  | Model 3: Material | 32.00 | 8.53 (-278.98 to 264.87) | -6.18 |
|  | Model 4: Psychosocial | 52.00 | 21.76 (-198.97 to 193.87) | -0.12 |
|  | Model 5: All Mediators | 48.00 | -26.99 (-464.34 to 384.58) | -0.43 |
| **Females** |  |  |  |  |
| Lowest | Model 2: Behavioural | -12.82 | -10.53 (-72.40 to 18.55) | -5.86 |
|  | Model 3: Material | -11.54 | -8.24 (-121.39 to 67.96) | -2.78 |
|  | Model 4: Psychosocial | 2.56 | 0.20 (-47.27 to 43.18) | -0.32 |
|  | Model 5: All Mediators | -48.72 | -16.49 (-151.67 to 56.28) | -0.12 |
| 2nd Quintile | Model 2: Behavioural | -7.81 | -12.18 (-98.83 to 24.51) | -2.49 |
|  | Model 3: Material | -14.06 | -9.28 (-142.61 to 70.62) | 8.45 |
|  | Model 4: Psychosocial | 1.56 | 6.85 (-23.33 to 43.32) | 10.05 |
|  | Model 5: All Mediators | -50.00 | -21.84 (-172.68 to 48.53) | -0.89 |
| 3rd Quintile | Model 2: Behavioural | -12.90 | 1.22 (-42.30 to 37.34) | 5.39 |
|  | Model 3: Material | -27.42 | -6.79 (-102.15 to 48.65) | 9.57 |
|  | Model 4: Psychosocial | 3.23 | 4.71 (-24.20 to 42.19) | 9.03 |
|  | Model 5: All Mediators | -56.45 | -4.23 (-99.75 to 50.45) | 2.22 |
| 4th Quintile | Model 2: Behavioural | 25.00 | 30.70 (-252.01 to 196.79) | 55.88 |
|  | Model 3: Material | 100.00 | -52.43 (-278.42 to 272.08) | 1.88 |
|  | Model 4: Psychosocial | 25.00 | -25.85 (-118.28 to 122.01) | -3.19 |
|  | Model 5: All Mediators | 100.00 | -153.20 (-505.83 to 479.97) | 233.82 |
| **Family occupation (Reference: Professional/Managerial technical)** |  |  |  |  |
| **Females** |  |  |  |  |
| Semi-skilled/Unskilled | Model 2: Behavioural | -4.05 | -2.30 (-51.67 to 28.27) | -3.01 |
|  | Model 3: Material | 31.08 | 37.80 (-25.93 to 164.49) | 5.92 |
|  | Model 4: Psychosocial | 12.16 | 17.95 (-7.57 to 64.82) | 19.55 |
|  | Model 5: All Mediators | 24.32 | 38.90 (-43.43 to 155.13) | -1.96 |
| Non manual/Skilled | Model 2: Behavioural | 10.53 | 2.06 (-197.58 to 172.33) | 3.60 |
|  | Model 3: Material | 26.32 | 44.34 (-302.51 to 378.36) | -0.46 |
|  | Model 4: Psychosocial | 10.53 | 25.79 (-183.80 to 228.48) | 14.64 |
|  | Model 5: All Mediators | 10.53 | 42.96 (-237.42 to 403.89) | -1.14 |
| **Medical card status (Reference: Not covered)** |  |  |  |  |
| **Males** |  |  |  |  |
| Yes, full card | Model 2: Behavioural | -1.54 | -2.49 (-31.16 to 18.46) | -4.78 |
|  | Model 3: Material | -10.77 | -16.89 (-152.33 to 55.60) | 5.59 |
|  | Model 4: Psychosocial | 36.92 | 31.71 (-7.21 to 95.80) | 14.84 |
|  | Model 5: All Mediators | 27.69 | 2.54 (-116.19 to 91.36) | -0.24 |
| Yes, doctor only card | Model 2: Behavioural | 2.50 | -6.66 (-137.89 to 135.30) | -0.45 |
|  | Model 3: Material | 2.50 | 7.07 (-155.02 to 177.79) | 1.55 |
|  | Model 4: Psychosocial | -15.00 | -0.65 (-73.31 to 91.35) | -0.81 |
|  | Model 5: All Mediators | 0.00 | 3.43 (-180.57 to 215.13) | -0.24 |
| **Females** |  |  |  |  |
| Yes, full card | Model 2: Behavioural | -19.51 | -11.86 (-84.36 to 27.14) | 1.45 |
|  | Model 3: Material | 36.59 | 35.45 (-81.58 to 195.42) | 1.58 |
|  | Model 4: Psychosocial | 0.00 | 6.47 (-67.87 to 79.37) | 6.97 |
|  | Model 5: All Mediators | -39.02 | 15.08 (-126.55 to 127.37) | -0.04 |
| Yes, doctor only card | Model 2: Behavioural | -27.91 | -39.74 (-284.42 to 200.58) | 5.26 |
|  | Model 3: Material | 39.53 | 34.47 (-250.47 to 290.39) | -16.06 |
|  | Model 4: Psychosocial | 13.95 | 4.79 (-134.62 to 134.75) | 23.02 |
|  | Model 5: All Mediators | -32.56 | -13.74 (-404.30 to 265.23) | -12.07 |

*Note:* PCOR = ((OR _Reference model_  - OR _Reference + more_ )/( OR _Reference model_  - 1)) X 100

*Where OR _Reference model_  = OR obtained in the first model, OR _Reference + more_ = OR obtained after adding additional variables in the reference model and PCOR = Percentage change in the magnitude of the first OR. Detailed confidence intervals for causal mediation natural indirect effects (NIE) are available in Table S7.*

Table S7 Comparison of mediation estimates across three analytic approaches for potential mediation of behavioural/material/psychosocial factors in the relationship between parent-reported oral health (Outcome: Three or more teeth with dental fillings) and PCG’s highest educational level/Family income quintiles/Family occupation/Medical card status among males and females. PCOR: Percentage change in Odds Ratio.

| **Exposure levels** | **Mediator Model** | **Baron & Kenny** | **Bootstrap** | **Causal Mediation** |
| --- | --- | --- | --- | --- |
| **Primary care giver highest education level (Reference: Tertiary)** |  | **PCOR (%)** | **PCOR (95% CI)** | **PCOR** |
| **Females** |  |  |  |  |
| None or Primary | Model 2: Behavioural | 4.40 | -10.21 (-193.27 to 148.69) | 10.78 |
|  | Model 3: Material | 10.99 | 30.04 (-176.67 to 278.09) | -1.29 |
|  | Model 4: Psychosocial | -21.98 | 7.49 (-92.70 to 114.99) | -19.14 |
|  | Model 5: All Mediators | -2.20 | 26.51 (-249.52 to 248.49) | -0.79 |
| Secondary | Model 2: Behavioural | 17.86 | 7.85 (-109.63 to 129.48) | 12.43 |
|  | Model 3: Material | 46.43 | 40.11 (-264.70 to 299.05) | 2.37 |
|  | Model 4: Psychosocial | 21.43 | 16.17 (-99.39 to 159.03) | 11.31 |
|  | Model 5: All Mediators | 25.00 | 41.16 (-297.35 to 393.53) | 0.45 |
| **Family Income Quintiles (Reference: Highest)** |  |  |  |  |
| **Males** |  |  |  |  |
| Lowest | Model 2: Behavioural | -3.45 | -7.06 (-77.07 to 35.73) | -31.72 |
|  | Model 3: Material | 55.17 | 54.00 (-47.67 to 257.80) | -4.02 |
|  | Model 4: Psychosocial | 10.34 | 18.90 (-15.43 to 99.04) | 1.54 |
|  | Model 5: All Mediators | 56.90 | 42.69 (-183.82 to 311.40) | -8.84 |
| 2nd Quintile | Model 2: Behavioural | -75.00 | -19.88 (-231.86 to 193.04) | -75.46 |
|  | Model 3: Material | 100.00 | 198.85 (-1022.66 to 1119.12) | -5.45 |
|  | Model 4: Psychosocial | 100.00 | 110.52 (-727.03 to 727.46) | -185.31 |
|  | Model 5: All Mediators | 100.00 | 373.15 (-996.88 to 1295.17) | 3.13 |
| 3rd Quintile | Model 2: Behavioural | -3.23 | -5.62 (-142.57 to 125.22) | 0.30 |
|  | Model 3: Material | 29.03 | 40.64 (-216.51 to 367.00) | 21.15 |
|  | Model 4: Psychosocial | 6.45 | 25.03 (-144.63 to 187.20) | 14.46 |
|  | Model 5: All Mediators | 41.94 | 43.35 (-287.42 to 475.96) | 0.89 |
| 4th Quintile | Model 2: Behavioural | 24.32 | -122.67 (-406.94 to 367.03) | 4.41 |
|  | Model 3: Material | 13.51 | 27.00 (-124.19 to 176.88) | 4.60 |
|  | Model 4: Psychosocial | -13.51 | 12.46 (-46.21 to 114.22) | -0.84 |
|  | Model 5: All Mediators | 56.76 | 18.29 (-317.37 to 280.92) | -0.06 |
| **Females** |  |  |  |  |
| Lowest | Model 2: Behavioural | 21.35 | 9.06 (-24.75 to 56.38) | 4.33 |
|  | Model 3: Material | 55.06 | 54.68 (-11.51 to 185.03) | 3.62 |
|  | Model 4: Psychosocial | 25.84 | 12.41 (-22.50 to 72.67) | -9.04 |
|  | Model 5: All Mediators | 43.82 | 58.24 (-12.39 to 216.61) | 3.13 |
| 2nd Quintile | Model 2: Behavioural | -33.33 | 220.61 (-436.94 to 352.78) | 334.51 |
|  | Model 3: Material | 100.00 | -2750.80 (-1437.45 to 1240.34) | -35.37 |
|  | Model 4: Psychosocial | 100.00 | -112.13 (-290.48 to 291.66) | 125.02 |
|  | Model 5: All Mediators | 100.00 | 2451.27 (-1109.27 to 1262.72) | 32.95 |
| 3rd Quintile | Model 2: Behavioural | -9.52 | -2.88 (-90.93 to 63.75) | 1.78 |
|  | Model 3: Material | 42.86 | 51.81 (-106.24 to 330.98) | 6.97 |
|  | Model 4: Psychosocial | 0.00 | 14.07 (-57.93 to 103.17) | 18.15 |
|  | Model 5: All Mediators | -19.05 | 40.80 (-29.33 to 161.95) | -1.10 |
| 4th Quintile | Model 2: Behavioural | 55.56 | 15.48 (-249.76 to 236.61) | -530.42 |
|  | Model 3: Material | 100.00 | 23383.78 (-361.88 to 471.45) | -55.59 |
|  | Model 4: Psychosocial | 100.00 | 59.06 (-159.58 to 131.19) | 501.34 |
|  | Model 5: All Mediators | -19.05 | 259.16 (-395.73 to 412.83) | -733.59 |
| **Family occupation (Reference: Professional/Managerial technical)** |  |  |  |  |
| **Females** |  |  |  |  |
| Semi-skilled/Unskilled | Model 2: Behavioural | 12.86 | -1.09 (-69.64 to 57.90) | 1.39 |
|  | Model 3: Material | 50.00 | 30.75 (-147.59 to 257.48) | -7.97 |
|  | Model 4: Psychosocial | 20.00 | -0.87 (-69.91 to 62.92) | 5.69 |
|  | Model 5: All Mediators | 58.57 | 25.22 (-247.57 to 281.20) | -1.87 |
| Non manual/Skilled | Model 2: Behavioural | 20.51 | 18.06 (-17.33 to 97.85) | 16.83 |
|  | Model 3: Material | 25.64 | 31.77 (-43.21 to 198.12) | -2.20 |
|  | Model 4: Psychosocial | 17.95 | 7.62 (-41.72 to 70.60) | 7.36 |
|  | Model 5: All Mediators | 51.28 | 53.47 (-90.41 to 314.41) | 1.14 |
| **Medical card status (Reference: Not covered)** |  |  |  |  |
| **Males** |  |  |  |  |
| Yes, full card | Model 2: Behavioural | 2.27 | -9.90 (-73.13 to 25.43) | -1.61 |
|  | Model 3: Material | 11.36 | 14.84 (-99.81 to 163.11) | -12.97 |
|  | Model 4: Psychosocial | 22.73 | 25.65 (-19.84 to 115.20) | 9.80 |
|  | Model 5: All Mediators | 31.82 | 6.04 (-157.19 to 148.61) | -3.28 |
| Yes, doctor only card | Model 2: Behavioural | 5.77 | 0.30 (-58.75 to 65.89) | -0.72 |
|  | Model 3: Material | -7.69 | -6.92 (-91.62 to 54.00) | -3.03 |
|  | Model 4: Psychosocial | -3.85 | -0.75 (-45.14 to 46.41) | 1.29 |
|  | Model 5: All Mediators | -1.92 | -5.44 (-96.42 to 81.53) | 0.16 |
| **Females** |  |  |  |  |
| Yes, full card | Model 2: Behavioural | 4.84 | 1.30 (-33.99 to 34.92) | 8.80 |
|  | Model 3: Material | 50.00 | 35.56 (-59.97 to 158.32) | 5.33 |
|  | Model 4: Psychosocial | 24.19 | 10.67 (-50.52 to 79.87) | 5.08 |
|  | Model 5: All Mediators | 33.87 | 40.68 (-60.98 to 165.24) | 0.05 |
| Yes, doctor only card | Model 2: Behavioural | -34.62 | -12.48 (-209.02 to 185.18) | -22.36 |
|  | Model 3: Material | 53.85 | 58.62 (-309.05 to 374.96) | 33.65 |
|  | Model 4: Psychosocial | 100.00 | 2.24 (-133.32 to 154.36) | -33.11 |
|  | Model 5: All Mediators | 100.00 | -51.44 (-404.55 to 397.25) | 3.07 |

*Note:* PCOR = ((OR _Reference model_  - OR _Reference + more_ )/( OR _Reference model_  - 1)) X 100

*Where OR _Reference model_  = OR obtained in the first model, OR _Reference + more_ = OR obtained after adding additional variables in the reference model and PCOR = Percentage change in the magnitude of the first OR. Detailed confidence intervals for causal mediation natural indirect effects (NIE) are available in Table S6.*

**Reference:**

Imai, K., Keele, L. and Tingley, D. (2010) 'A general approach to causal mediation analysis', *Psychol Methods,* 15(4), pp. 309-34.
